# Analysis of Immune Gene Expression Subtypes Reveals Osteosarcoma Immune Heterogeneity

**DOI:** 10.1155/2021/6649412

**Published:** 2021-03-01

**Authors:** Ben Wan, Renxian Wang, Jingjun Nie, Yuyang Sun, Bowen Zhang, Weifeng Liu, Dafu Chen

**Affiliations:** ^1^Laboratory of Bone Tissue Engineering, Beijing Laboratory of Biomedical Materials, Beijing Research Institute of Traumatology and Orthopaedics, Beijing JiShuiTan Hospital, Beijing 100035, China; ^2^Depatment of Orthopaedic Oncology Surgery, Beijing JiShuiTan Hospital, Peking University, Beijing 100035, China

## Abstract

**Background:**

Osteosarcoma (OS) patients have a poor response to immunotherapy due to the sheer complexity of the immune system and the nuances of the tumor-immune microenvironment. *Methodology*. To gain insights into the immune heterogeneity of OS, we identified robust clusters of patients based on the immune gene expression profiles of OS patients in the TARGET database and assessed their reproducibility in an independent cohort collected from the GEO database. The association of comprehensive molecular characterization with reproducible immune subtypes was accessed with ANOVA. Furthermore, we visualized the distribution of individual patients in a tree structure by the graph structure learning-based dimensionality reduction algorithm.

**Results:**

We found that 87 OS samples can be divided into 5 immune subtypes, and each of them was associated with distinct clinical outcomes. The immune subtypes also demonstrated widely different patterns in tumor genetic aberrations, tumor-infiltrating, immune cell composition, and cytokine profiles. The immune landscape of OS uncovered the significant intracluster heterogeneity within each subtype and depicted a continuous immune spectrum across patients.

**Conclusion:**

The established five immune subtypes in our study suggested immune heterogeneity in OS patients and may provide optimal individual immunotherapy for patients exhibiting OS.

## 1. Introduction

Osteosarcoma (OS) is the most common primary bone tumor mainly found in pediatric patients. It originates from mesenchymal stem cells, characterized by osteoid production [1]. Despite their rare incidence, OS has a high disability and mortality rate. The overall 5-year survival rate for patients with localized OS is about 65%, while it is around 30% for patients with metastatic or relapsed OS [2, 3]. The conventional therapeutic procedure for OS combines surgical resection and chemotherapy. Unfortunately, current standard treatment cannot further benefit patients with advanced OS due to chemotherapy resistance and early metastases [4].

Immunotherapy, as a new biological therapy, uses various strategies to enhance antitumor immunity [5]. Tumor speciﬁc immunotherapy such as *γδ* T cell-based fusion vaccine and chimeric antigen receptor- (CAR-) engineered T cells displayed outstanding antitumor performance in vitro and in vivo [6, 7]. Moreover, adjuvant immune therapies may prolong the survival of advanced OS patients who received tumor-infiltrating lymphocytes therapy [8]. However, recent clinical trial findings revealed progressed OS patients' poor response to immunomodulators such as PD-1 inhibitors, IL-2 [9–11]. These discrepancies may be partially due to their complex and dynamic tumor immune microenvironment where mesenchymal cells, tumor-infiltrating immune cells (TIICs), endothelial cells, extracellular matrix molecules, and inflammatory mediators interact with tumor cells. Although some immune-related gene signature has been recently revealed via bioinformatics analysis [12–14], no studies reported a comprehensive immune characterization specifically for OS.

In our study, we identified five robust OS immune subtypes based on the ConsensusClusterPlus algorithm and further validated their reproducibility in an independent Gene Expression Omnibus (GEO) dataset. The five immune subtypes were associated with distinct gene expression patterns, molecular and cellular characteristics, as well as clinical outcomes. Finally, our work characterized a complex immune landscape of OS and revealed the intracluster heterogeneity within the immune subtype.

## 2. Materials and Methods

### 2.1. Materials

The gene expression profile and clinical follow-up information data of the OS training dataset were downloaded from the Therapeutically Applicable Research to Generate Effective Treatments (https://ocg.cancer.gov/programs/target) initiative, which contains a total of 101 samples (Supplementary Table S1). For validation, we downloaded GSE30699 cohort data from GEO database (https://www.ncbi.nlm.nih.gov/geo/), which contains 107 samples.

### 2.2. Sources of Immune-Related Genes

Through the method of literature mining, the following types of genes were collected as immune-related genes for subsequent analysis: immune cell-specific genes derived from single-cell RNA-seq data, genes of costimulatory, and cosuppressive molecules, cytokines, and cytokine receptors [15]. The genes were involved in antigen processing and presentation, and other immune-related biological processes. A total of 1989 immune-related genes were collected (Supplementary Table S2).

### 2.3. Patient Selection and Data Preprocessing

The 87 OS samples were obtained after the following steps were performed on the RNA-seq data of the TARGET data. (1) Remove samples without clinical data; (2) remove genes whose expression level (FPKM) is equal to 0, above 50% of the samples; and (3) the expression profiles of immune-related genes were preserved and log conversion was performed in log2 (FPKM + 1). We obtained 76 OS samples from GEO datasets by deleting cell line and xenotransplant sample data, removing probes with empty gene detection values, mapping the chip probes to human genes, and retaining the expression profile of immune cell-related genes.

### 2.4. Identification of Immune Subtypes and Immune Gene Modules

ConsensusClusterPlus was used to identify robust clusters of patients [16]. Based on the expression data of 1989 immune-related genes, the immune subtypes (IS) and immune gene modules (GM) of the samples were obtained. Then, we used the in-group proportion (IGP) [17] and Pearson correlation among the centroid of the gene module scores to quantitatively measure the consistency of subtype identification in the training and validation cohorts (Supplementary Methods). The ANOVA algorithm was used to evaluate the association between immune subtypes and 64 immune-related molecular and cellular characteristics (Supplementary Methods).

### 2.5. Assessment of Clinical, Molecular, and Cellular Characteristics Related to Immune Subtypes

We used log-rank tests and multivariate Cox regression to assess the prognostic value of immune subtypes in training set samples with race, age, gender, and location as covariates, using overall survival (OS) and progression-free survival (PFS) as the endpoints. Then, in the validated set of samples, the correlation between immunological subtypes and various immune-related molecules and cell characteristics was evaluated by ANOVA.

### 2.6. Constructing the Immune Landscape

We performed a dimensionality reduction analysis using a graph learning-based method to reveal the intrinsic structure and visualize the distribution of individual patients due to the dynamic characteristics of the immune system (Supplementary Methods). Simply put, this analysis projects high-dimensional gene expression data into tree structures in low-dimensional spaces by retaining local geometric information [18]. This approach has previously been used to simulate cancer progression using large and single-cell gene expression data and to define the trajectory of cancer [19, 20]. In this study, we applied the analysis to immune gene expression profiling. This immune landscape reflects the relationship between patients in a nonlinear manifold, which may complement the discrete immune subtypes defined in linear Euclidean space. After defining the immune landscape, intracluster heterogeneity of immune subtypes was evaluated by ANOVA. The log-rank test was used to compare the survival difference of the IS2 subgroup.

### 2.7. Statistical Analysis

All statistical analyses and data visualization were performed in *R* version 3.6.3 (Supplementary Methods). To compare gene expression data from RNA-Seq expression, we calculated Spearman rank correlations of gene expression for all possible gene pairs across the samples. Samples with complete clinical data were included in survival analysis, and the log-rank test was performed for comparing Kaplan–Meier curves between subgroups. We used a one-way analysis of variance (ANOVA) to measure the statistical signiﬁcance of the calculated results. *P* value < 0.05 indicated statistical significance.

## 3. Results

### 3.1. Immune Subtypes and Gene Modules Construction

**Figure 1:**
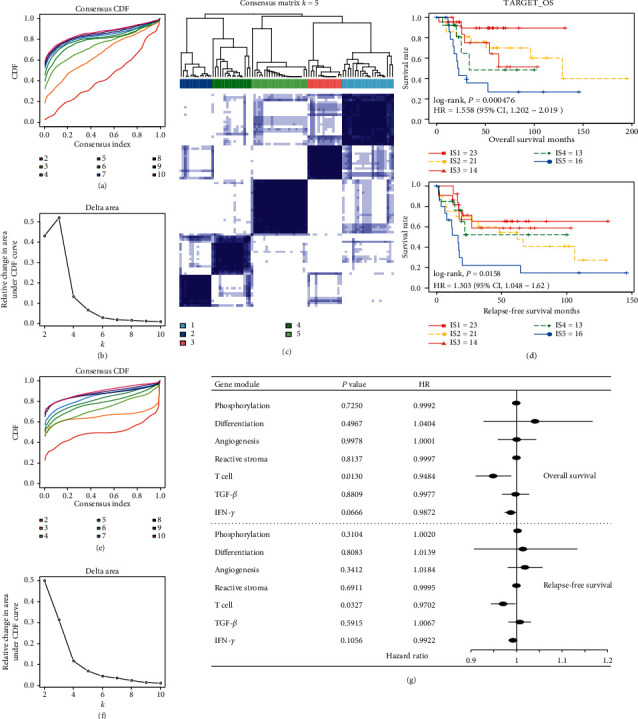
The immune subtypes and gene modules in OS. (a) The cumulative distribution function (CDF) curve of training sample; (b) CDF delta area curve of training sample. Delta area curve of consensus clustering indicates the relative change in area under the CDF curve for each category number *k* compared with *k* − 1. The horizontal axis represents the category number *k*, and the vertical axis represents the relative change in area under the CDF curve; (c) Sample clustering heat map when consensus *k* = 5; (d) Kaplan–Meier curves for five immune subtypes prognosis. (e). CDF curve of immune gene; (f) CDF delta area curve of immune gene; (g) Univariate Cox analysis results of gene modules.

We extracted the OS expression profile of immune-related genes from the TARGET database and got 1922 genes for subsequent analysis. The 87 OS samples are clustered through ConsensusClusterPlus, and the optimal number of clusters is determined according to the cumulative distribution function (CDF). The CDF delta area showed that when consensus index is five, the clustering result is relatively stable (Figures 1(a) and 1(b)). Finally, we choosed *k* = 5 to get five immune subtypes (Figure 1(c) and Supplementary Table S3). Kaplan–Meier curves revealed that there are significant prognostic differences between immune subtypes (OS: log-rank, *P* = 4.76*E* − 04; RFS: log-rank, *P* = 0.015; Figure 1(d)). The prognostic difference in the overall survival is independent of other clinical factors (race, age, gender, and location; Table 1), while there was no statistical difference in relapse-free survival, indicating that the immune subtype had a higher correlation with the overall survival of the OS patients. Overall, IS1 was associated with the best prognosis for both OS and PFS. In contrast, IS5 was the worst among all subtypes.

Similarly, we identified 7 immune-related gene modules (Figures 1(e) and 1(f), Supplementary Figure S1 and Table S4). We found that some gene modules are significantly related to the prognosis of OS (Figure 1(g)). Consistent with previous reports, our results showed high scores of T cell, and IFN-*γ* modules predict a good prognosis.

### 3.2. Functional and Robust Gene Modules

Gene modules appeared to be more closely clustered compared with immune subtypes. The functions of gene modules correspond to phosphorylation, reactive stroma, T cell, inflammation, differentiation, TGF-*β*, and IFN-*γ* (Supplementary Figure S2 and Table S5). Particularly, our gene module of reactive stroma was consistent with a previously proposed 25-gene stromal signature [21, 22], in which 18 genes were assigned to this module and 21 genes were included in our immune-related gene set. Moreover, gene module 5 was defined as TGF-*β* related due to its correlation with the TGF-*β* response score (Spearman *ρ* = 0.45; *P* = 1.45*E* − 05).

**Figure 2:**
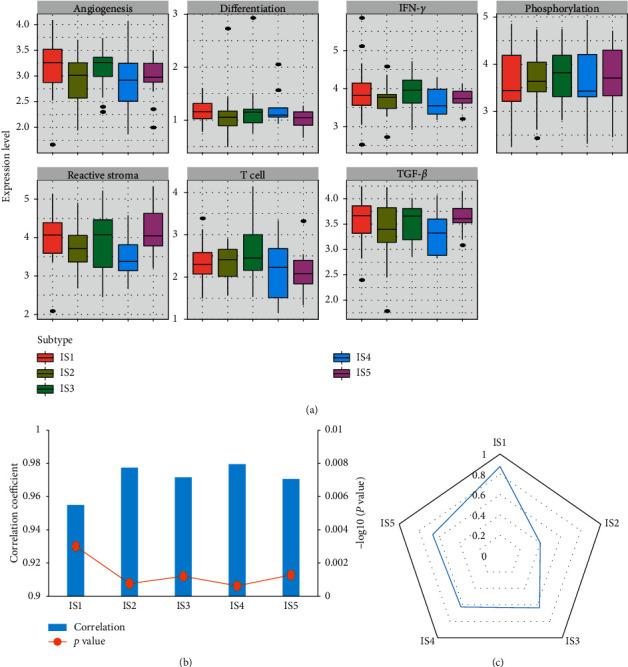
The association of gene modules with the immune subtypes. (a) The middle bar in each box represents the median level of corresponding gene modules in certain immune subtype. (b) Correlation of average gene module scores between immune subtypes of the discovery and the validation cohort. (c) IGP assesses the similarity and reproducibility of the proposed immune subtypes between discovery and validation cohorts.

To verify the immune subtypes identified from the TARGET database, we downloaded and analyzed a cohort of OS cases from the GEO database (*n* = 76), an independent OS dataset (accession number: GSE30699, Supplementary Figure S3). The expression patterns of gene modules were highly consistent between the training and validation cohorts with an average linear correlation of 0.97 (Figure 2(b)). At the individual patient level, there was a moderate to good agreement between the two cohorts (IGP from IS1 to IS5: 0.878, 0.4, 0.636, 0.625, and 0.667; Figure 2(c)).

### 3.3. Molecular and Cellular Characteristics of the Immune Subtypes

**Figure 3:**
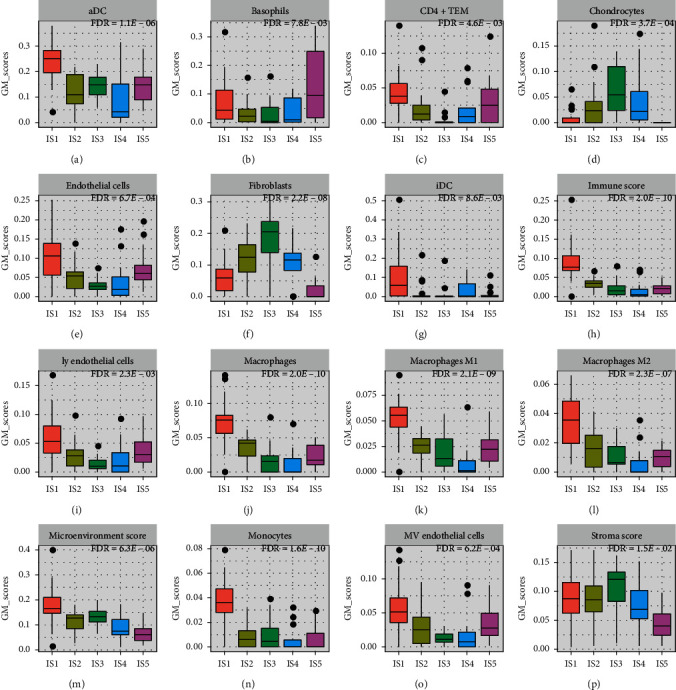
Molecular and cellular characteristics associated with the immune subtypes. (a)-(p) The middle bar in each box represents the median level of corresponding features in certain immune subtype. The FDR-adjusted *P* values for all features were less than 0.05.

We assessed the relation between the immune subtypes and 64 previously defined immune-related molecular features (Supplementary Table S6). Consistent with the immunosuppression phenotype, tumors in IS5 had high basophils fraction (Figure 3(b)) and the lowest microenvironment signature score (Figure 3(m) and Supplementary Table S7). The most basophils infiltration signature score would promote high infiltration of immunosuppressive cells, TH2, generating an immune-cold microenvironment. Overall, IS2, IS3, and IS4 are closely related to IS5 with the respect of the immune score, macrophage (Figure 3(h)). IS1 demonstrated a favorable immune profile and was associated with the best prognosis. Compared to the IS5 phenotype with the worst prognosis, IS1 tumors had the highest effector memory (TEM) CD4+ T cells and immune score. Of note, IS1 was enriched with dendritic cells (DCs), macrophages, monocytes (Figures 3(a), 3(h), 3(j), and 3(n)).

### 3.4. Immune Landscape of OS

**Figure 4:**
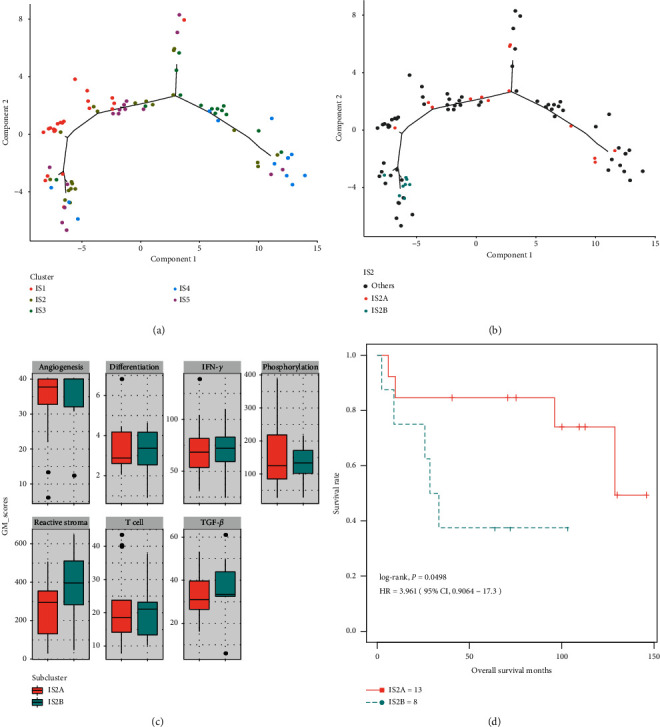
The immune landscape of OS and the intracluster heterogeneity within immune subtype 2. (a) The immune landscape of OS: each point represents a patient with colors corresponding to the immune subtype defined previously. (b) Patients of immune subtype 2 could be further stratified into 2 subgroups based on their location in the immune landscape. (c) Gene module expression patterns were shown to illustrate the intracluster heterogeneity of immune subtype 2. (d) The two subgroups of patients in immune subtype 2 as stratified by the immune landscape were associated with distinct prognoses. Log-rank *P* value was calculated among subgroup stratification.

Graph learning-based dimensionality reduction techniques were applied to facilitate visualization and reveal the underlying structure of individual patient distribution. This analysis placed individual patients in a graphic with sparse tree structure (Figure 4(a)) and defined the immune landscape of OS. The position of the patient in the immune landscape represents the overall characteristics of the corresponding subtype of tumor immune microenvironment (Figure 4(a)). Indeed, the horizontal coordinate was highly negatively correlated with IFN-*γ* and T cell (*ρ* = −0.40 and −0.75, respectively; both *P* < 0.001) and was highly positively correlated with TGF-*β* and differentiation (*ρ* = 0.41 and 0.45, respectively; both *P* < 0.001).This is consistent with the result that we found that IS3 has an increased T cell compared to IS5. Moreover, the horizontal coordinate has the highest correlation with reactive stroma module (*ρ* = −0.82, respectively; *P* < 2.2 × 10^−16^). Correspondingly, IS1 and IS5 have the lowest and highest reactive stroma scores, respectively (Figure 2(a)). On the other hand, the vertical coordinate is significantly related to the differentiation module (*ρ* = 0.52, respectively; *P* < 0.001).

Immune landscape analysis further revealed significant intraclass heterogeneity in each subtype. We found that the specific subtypes are more diverse and heterogeneous than other subtypes (Supplementary Figure S4). For example, IS1 tumors can be further divided into three subtypes according to their location in the immune landscape, which are manifested as specific immune expression patterns. Similar results were observed in IS4. Interestingly, the two subtypes of IS2 tumors were further divided according to the immune landscape (Figure 4(b)), showing different gene expression and prognosis patterns (Figures 4(c) and 4(d)). The immune landscape analysis provides further supplementary results for the immune subtypes we previously defined.

## 4. Discussion

As not all OS patients have greater benefit from immunotherapy, more immune microenvironment characteristics should be incorporated to instruct clinical treatment. Researchers have made great effort to reveal the role of tumor microenvironment and tumor microenvironment-related genes in OS by a series of bioinformatics methods [12–14, 23, 24]. In the present study, the ConsensusClusterPlus algorithm was utilized to identify the five reproducible immune subtypes in 87 OS patients from the TARGET database. Moreover, we first used a graph learning-based method to depict immune landscape and intracluster heterogeneity spectrum in OS. To sum up, we provided a better way of understanding the OS immune microenvironment and revealing OS immune heterogeneity.

The effect of tumor immune microenvironment on patient survival has been well documented in many types of cancer [22, 25–27]. OS is no exception, and the tumor immune microenvironment is closely related to the prognosis of OS patients. Zhang et al. first established the prognostic signature based on immune microenvironment-related genes for OS [14], and Hu et al. performed comprehensive analysis of prognostic tumor microenvironment-related genes based on several validated genes [23]. Song et al. identified a set of immune gene signature related to clinical response and was veriﬁed based on 45 OSA primary tumors [12]. Our work differs from these studies in several important aspects. First, we divided patients into five immune subtypes with different molecular and prognostic characteristics and further assessed their reproducibility in an independent cohort. Second, we used a comprehensive set of genes to reflect various immunological processes instead of using established signatures. Third, we applied graph learning approaches to uncover the overall structure of the patient distribution and capture intercluster and intracluster relationships.

In our study, OS of IS1 demonstrated the highest levels of infiltration by immune effectors such as CD4^+^ T and activated dendritic cells (DCs). Accordingly, patients in subtype 1 had the best prognosis. In comparison, tumors of subtype 5 had elevated basophils infiltration and reduced monocytes. Thus, these patients appeared to have the worst survival. The other immune subtypes demonstrated a similar level of immune infiltration according to the result of the microenvironment score and immune score. However, the immune landscape recapitulated the immune subtypes based on clustering analyses and uncovered previously unappreciated intracluster heterogeneity with potential clinical significance. For example, tumors of subtype 2 demonstrated the intracluster heterogeneity. The immune composition of IS2B was dominated by highly immune-suppressive factors such as TGF-*β* signaling and reactive stroma which had bad prognosis. These data add to the accumulating evidence that the suppressive factors are critical in determining prognosis.

The immune subtypes' analysis relies on the immune-related gene expression profiles to reveal the underlying structures of the immune landscape within tumors, although an individual-based model was used to develop predictive and prognostic biomarkers. It is conceivable that a hierarchical model may be used to predict clinical outcomes by stratifying patients into subgroups. The idea of “subtype-specific” biomarkers has been successfully applied to improve outcome prediction in breast, glioma, and colon cancers [28–30]. Therefore, integrating subtype analyses and the individual-based model would be a promising approach to developing clinically relevant biomarkers.

On the other hand, our study has potential therapeutic implications for the rational design of combination immunotherapy strategies. For patients with a favorable immune microenvironment (e.g., subtype 1), immune checkpoint blockade (ICB) may benefit these patients and further improve their survival. As we described above, OS has a high level of immune heterogeneity, and some tumors expressing the programmed cell death protein-1 ligand (PD-L1) may be potential sensitivities to inhibitors of the programmed cell death protein-1 (PD-1)/PD-L1 axis [31, 32]. However, it may be ineffective for treating patients in subtype 5 with ICB alone due to the suboptimal immune activation or presence of immune-suppressive mechanisms. Therefore, combination of ICB with immune costimulatory modulators such as mifamurtide (an approved macrophage activator) and interleukin-2 may be used to boost the weak immune response for patients in subtype 5 [10, 33]. For the remaining patients in subtypes 2, 3, and 4, depending on their specific immune cells infiltration and stromal microenvironment, both nonspeciﬁc immunotherapy and tumor speciﬁc immunotherapy might be used together with conventional chemotherapy to improve patients' prognosis [5].

Although the immune heterogeneity of different OS immune subtypes have been initially studied by bioinformatic and statistical analyses in our study, some limitations should be elucidated. First, we cannot obtain the treatment information from the TARGET database and GEO dataset, which may influence the prognosis of OS patients. Second, our approach is “unsupervised,” which means the underlying structures of the immune landscape within tumors rely on the immune-related gene expression profiles. Third, the validation cohort was generated and confirmed good reproducibility of the five immune subtypes in our research. However, independent validation by a large cohort is needed.

## 5. Conclusions

In conclusion, we identified 5 reproducible immune subtypes of OS with distinct molecular characteristics and clinical outcomes. The immune landscape of the tumor immune microenvironment was investigated to demonstrate OS immune heterogeneity. Our study provides a new perspective for the study of immune heterogeneity of OS, allowing for understanding individual differences in immunotherapy.

## Figures and Tables

**Table 1:** Univariate and multivariable Cox regression analyses of OS and RFS.

	Factors	Univariate Cox			Multivariable Cox		
		HR	95% CI	*P*	HR	95% CI	*P*
Overall survival	Immune subtype	1.6	1.2–2	4.76*E* − 04	2.4	1.2–5	0.0148
	Gender	0.75	0.34–1.7	0.48	4.1	0.75–23	0.104
	Race	0.78	0.31–1.9	0.587	0.25	0.043–1.4	0.121
	Age	1	1–1	0.813	1	1–1	0.374
	Location	0.37	0.12–1.1	0.0697	0.17	0.022–1.3	0.0919

Relapse-free survival	Immune subtype	1.3	1–1.6	0.0158	1.4	0.87–2.2	0.174
	Gender	1.2	0.6–2.2	0.658	2.8	0.76–11	0.12
	Race	0.66	0.3–1.4	0.291	0.33	0.061–1.8	0.203
	Age	1	1–1	0.248	1	1–1	0.878
	Location	0.34	0.13–0.87	0.0179	0.27	0.075–1	0.0507

## Data Availability

The training and validating data used to support the findings of this study have been deposited in the TARGET and GEO repository (GSE30699).

## Supplementary Materials

Supplementary Materials Supplementary Figure S1. Consensus matrix for immune-related gene modules derived from consensus clustering analysis. Supplementary Figure S2. Expression feature of immune subtypes in training TARGET cohort. Supplementary Figure S3. The validation of immune subtypes in the independent GEO cohort. Supplementary Figure S4. The intracluster heterogeneity revealed by the immune landscape analysis and different subcluster molecular characteristics of patients in immune subtypes 1 and 4 are shown. Supplementary Table S1. The bulk RNA-seq data retrieved from the OS-project. Supplementary Table S2. The collected 1989 immune-related genes. Supplementary Table S3. The immune subtype annotation at the patient level in the TARGET cohort. Supplementary Table S4. The immune-related gene modules feature. Supplementary Table S5. Functional enrichment analysis of gene modules. Supplementary Table S6. The immune-related molecular features of the TARGET patients. Supplementary Table S7. Correlation of average gene module scores between molecular characteristics and immune subtypes.
