# Supplementary material for: Analysis of Immune Gene Expression Subtypes Reveals Osteosarcoma Immune Heterogeneity
Source: J Oncol. 2021 Mar 1;2021:6649412. doi: 10.1155/2021/6649412 (PMC7939746; doi:10.1155/2021/6649412)
Supplement: Supplementary Materials — Supplementary Figure S1. Consensus matrix for immune-related gene modules derived from consensus clustering analysis. Supplementary Figure S2. Expression feature of immune subtypes in training TARGET cohort. Supplementary Figure S3. The validation of immune subtypes in the independent GEO cohort. Supplementary Figure S4. The intracluster heterogeneity revealed by the immune landscape analysis and different subcluster molecular characteristics of patients in immune subtypes 1 and 4 are shown. Supplementary Table S1. The bulk RNA-seq data retrieved from the OS-project. Supplementary Table S2. The collected 1989 immune-related genes. Supplementary Table S3. The immune subtype annotation at the patient level in the TARGET cohort. Supplementary Table S4. The immune-related gene modules feature. Supplementary Table S5. Functional enrichment analysis of gene modules. Supplementary Table S6. The immune-related molecular features of the TARGET patients. Supplementary Table S7. Correlation of average gene module scores between molecular characteristics and immune subtypes. [file 6649412.f1.zip › 6649412.f1/Supplementary Methods-revised.docx]

**Supplementary Methods**

**Study design**

The overall design of our study is to identify immune subtypes and depict immune landscape in OS. First, by integrating single-cell and bulk tumor RNA-seq data, as well as prior knowledge about the immune system, we collected a comprehensive set of genes reflecting various immunological processes. Based on the immune gene expression profiles of OS in GEO, we used consensus clustering to identify immune subtypes and functional gene modules. The identified immune subtypes were validated in an independent OS cohort in the TARGET. We further evaluated the clinical, molecular and cellular characteristics associated with the immune subtypes. Finally, we applied graph structure learning-based dimensionality reduction analysis to visualize the distribution of individual patients and elucidate the immune landscape.

**Patient selection and data preprocessing:**

For the discovery cohorts TARGET, the bulk RNA-seq data were retrieved from the OS-project (Supplementary Table S1)

For the GSE30699 validation cohort,. normalized gene expression data of the Illumina platform were retrieved from the GEO database. Entrez IDs were used to match genes across corresponding platform. In the validation cohort, immune-related genes (described below) were selected. Each gene expression was transformed by log2 across patients in both discovery and validation cohorts.

A variety of molecular and cellular immune related features were also retrieved for the patients in the TARGET cohort (described below) (1).

**Identification of immune subtypes and gene modules**

After curating the immune-related gene profiles, we then applied the partition around medoids (PAM) algorithm with the Euclidean distance metric and performed 500 bootstraps each encompassing 80% patients of the discovery cohort. The number of clusters was varied from 2 to 10, and the optimal partition was determined by evaluating the consensus matrix and the consensus cumulative distribution function (2). To identify robust immune gene modules, we also applied consensus clustering using the same settings and parameters, except for the distance metric using 1 - Pearson correlation.

Next, scores for gene modules (GM scores) were defined as the average expression level of all genes in a particular module. Spearman correlation was evaluated between GM score and molecular signatures (1) (described below). The overlap between genes of an established reactive stroma signature (3) and GM was also assessed.

**Validation of the immune subtypes in the GEO cohort**

The in-group-proportion (IGP) statistic (4) was used to quantitatively assess the similarity and reproducibility of the proposed immune subtypes between discovery and validation cohorts, at individual patient level as well as for gene expression patterns. IGP values range from 0 to 1. A high IGP for a subtype corresponds to a reproducible partition of patients for that subtype. Specifically, we first identified the immune-related genes shared by the discovery and validation cohort and focused on the expression profiles of common genes in both cohorts. To estimate IGP, we then calculated the centroid of each immune subtype of the shared immune-related genes in the discovery cohort. Then, each sample in the validation cohort was assigned to an immune subtype whose centroid had the highest Pearson correlation with the sample, and the IGP was estimated for each subtype in the validation cohort. In other words, the IGP measures the proportion of patients classified to an immune subtype whose nearest neighbors were also classified to the same immune subtype. The statistical significance of IGP was estimated with 500 permutations (package clusterRepro) (4).

To validate the gene expression modules associated with the immune subtypes, we trained a partition around medoids (PAM) classifier in the discovery cohort to predict the immune subtype for patients in the validation cohort. Then, for each immune subtype in the discovery and validation cohorts, we generated the centroids of the 7 gene module scores. The pairwise Pearson correlation among those centroids in the two cohorts was calculated to assess the one-to-one correspondence in gene expression modules between the two datasets.

**Immune-related molecular and cellular features**

We assessed the relation between the immune subtypes and 64 immune-related molecular and cellular features(5). We analyzed the immune cell composition in the tumor tissue inferred by the xCell algorithm.

**Immune landscape analysis**

The graph learning-based dimensionality reduction analysis was conducted using the reduceDimension function of Monocle (6) package with a Gaussian distribution. The maximum number of components was set to 4, and the discriminative dimensionality reduction with trees (DDRTree) was used as the dimension reduction method. Finally, the immune landscape was visualized with the function plot_cell_trajectory (package Monocle (6)) with the color corresponding to the immune subtype identified above.

All statistical analyses were performed in R version 3.6.3.

**References:**

1. Thorsson V, Gibbs DL, Brown SD, Wolf D, Bortone DS, Ou Yang TH*, et al.* The Immune Landscape of Cancer. Immunity **2018**;48:812-30 e14

2. Wilkerson MD, Hayes DN. ConsensusClusterPlus: a class discovery tool with confidence assessments and item tracking. Bioinformatics **2010**;26:1572-3

3. Moffitt RA, Marayati R, Flate EL, Volmar KE, Loeza SG, Hoadley KA*, et al.* Virtual microdissection identifies distinct tumor- and stroma-specific subtypes of pancreatic ductal adenocarcinoma. Nat Genet **2015**;47:1168-78

4. Kapp AV, Tibshirani R. Are clusters found in one dataset present in another dataset? Biostatistics (Oxford, England) **2007**;8:9-31

5. Breuer K, Foroushani AK, Laird MR, Chen C, Sribnaia A, Lo R*, et al.* InnateDB: systems biology of innate immunity and beyond-recent updates and continuing curation. Nucleic Acids Research **2013**;41:D1228-D33

6. Trapnell C, Cacchiarelli D, Grimsby J, Pokharel P, Li S, Morse M*, et al.* The dynamics and regulators of cell fate decisions are revealed by pseudotemporal ordering of single cells. Nature biotechnology **2014**;32:381
