# Supplementary material for: Analysis of Immune Gene Expression Subtypes Reveals Osteosarcoma Immune Heterogeneity
Source: J Oncol. 2021 Mar 1;2021:6649412. doi: 10.1155/2021/6649412 (PMC7939746; doi:10.1155/2021/6649412)
Supplement: Supplementary Materials — Supplementary Figure S1. Consensus matrix for immune-related gene modules derived from consensus clustering analysis. Supplementary Figure S2. Expression feature of immune subtypes in training TARGET cohort. Supplementary Figure S3. The validation of immune subtypes in the independent GEO cohort. Supplementary Figure S4. The intracluster heterogeneity revealed by the immune landscape analysis and different subcluster molecular characteristics of patients in immune subtypes 1 and 4 are shown. Supplementary Table S1. The bulk RNA-seq data retrieved from the OS-project. Supplementary Table S2. The collected 1989 immune-related genes. Supplementary Table S3. The immune subtype annotation at the patient level in the TARGET cohort. Supplementary Table S4. The immune-related gene modules feature. Supplementary Table S5. Functional enrichment analysis of gene modules. Supplementary Table S6. The immune-related molecular features of the TARGET patients. Supplementary Table S7. Correlation of average gene module scores between molecular characteristics and immune subtypes. [file 6649412.f1.zip › 6649412.f1/Supplementary Table S1.pdf]

sample	RFS_Event	RFS_time
TARGET.40.PALWWX	0	2163
TARGET.40.PAMEKS	1	385
TARGET.40.PAMLKS	1	71
TARGET.40.PAMRHD	1	39
TARGET.40.PAMYYJ	1	128
TARGET.40.PANMIG	1	513
TARGET.40.PANPUM	1	46
TARGET.40.PANZHX	1	812
TARGET.40.PAPIJR	0	1719
TARGET.40.PAPKWD	1	192
TARGET.40.PAPNVD	1	594
TARGET.40.PAPWWC	1	1933
TARGET.40.PAPXGT	0	2526
TARGET.40.PARBGW	0	286
TARGET.40.PARDAX	1	105
TARGET.40.PARFTG	1	53
TARGET.40.PARGTM	0	2610
TARGET.40.PARJXU	1	811
TARGET.40.PARKAF	1	56
TARGET.40.PASEBY	1	42
TARGET.40.PASEFS	0	2112
TARGET.40.PASFCV	0	2062
TARGET.40.PASKZZ	0	542
TARGET.40.PASSLM	1	1865
TARGET.40.PASUUH	0	1625
TARGET.40.PASYUK	0	1870
TARGET.40.PATAWV	0	1702
TARGET.40.PATJVI	0	1575
TARGET.40.PATKSS	1	63
TARGET.40.PATMIF	0	1580
TARGET.40.PATMPU	1	498
TARGET.40.PATPBS	0	1468
TARGET.40.PATXFN	0	1363
TARGET.40.PASNZV	0	1747
TARGET.40.PASRNE	1	512
TARGET.40.PATEEM	1	961
TARGET.40.PATUXZ	1	510
TARGET.40.PAUBIT	0	1029
TARGET.40.PAUTWB	1	598
TARGET.40.PAUTYB	1	527
TARGET.40.PAUUML	0	672
TARGET.40.PAUVUL	1	237
TARGET.40.PAUXPZ	1	419
TARGET.40.PAUYTT	0	579
TARGET.40.PAVALD	0	551
TARGET.40.PAVCLP	0	511
TARGET.40.PAVDTY	0	369
TARGET.40.PAVECB	0	476
TARGET.40.PATMXR	1	68
TARGET.40.0A4HLD	0	3946
TARGET.40.0A4HMC	0	377

TARGET.40.0A4HX8	0	3050
TARGET.40.0A4HXS	0	2948
TARGET.40.0A4HY5	1	218
TARGET.40.0A4HYB	0	1240
TARGET.40.0A4I0Q	1	519
TARGET.40.0A4I0S	0	603
TARGET.40.0A4I0W	0	683
TARGET.40.0A4I3S	0	1844
TARGET.40.0A4I42	1	351
TARGET.40.0A4I48	0	1616
TARGET.40.0A4I4E		
TARGET.40.0A4I4M	0	708
TARGET.40.0A4I4O	1	235
TARGET.40.0A4I5B	0	619
TARGET.40.0A4I6O	1	484
TARGET.40.0A4I8U	0	983
TARGET.40.PAPFLB		
TARGET.40.PAKFVX	1	361
TARGET.40.PAKXLD	1	578
TARGET.40.PAKZZK	1	452
TARGET.40.PAKUZU	0	0
TARGET.40.PALECC	1	384
TARGET.40.PALFYN	1	651
TARGET.40.PALHRL	0	4374
TARGET.40.PALKDP	0	3282
TARGET.40.PALKGN	0	2122
TARGET.40.PALZGU	1	1452
TARGET.40.PAMHLF	0	1913
TARGET.40.PAMHYN	0	3900
TARGET.40.PAMJXS	1	3184
TARGET.40.PAMTCM	0	3002
TARGET.40.PANGPE	1	833
TARGET.40.PANGRW	0	3378
TARGET.40.PANSEN	0	2256
TARGET.40.PANVJJ	0	3095
TARGET.40.PANXSC	1	366
TARGET.40.PANZZJ	0	1538
TARGET.40.PAPVYW	1	69
TARGET.40.0A4I65	1	1993
TARGET.40.0A4I9I	1	2014
TARGET.40.0A4I9K		2257

OS_Event	OS_time	Gender	Race	Age	location
0	2163	Female	White	4464	Distal
1	857	Male	Asian	3942	Distal
1	180	Female	White	4509	Distal
1	74	Female	Unknown	3942	Distal
1	271	Male	White	6477	
1	776	Male	White	5360	Proximal
1	295	Male	White	5996	
0	1323	Female	Asian	5127	
0	1719	Female	White	4597	Distal
1	422	Male	White	3478	Distal
1	922	Female	White	5289	Proximal
0	2520	Female	White	3595	Distal
0	2526	Female	Black or African American	4172	Proximal
0	286	Female	Unknown	4634	Distal
1	679	Male	White	3680	Distal
1	653	Female	Asian	4987	Distal
0	2610	Male	White	5925	Proximal
1	1631	Female	White	4624	Distal
1	1061	Male	White	6085	Distal
0	2049	Male	White	4249	Distal
0	2112	Male	White	6625	Proximal
0	2062	Female	White	3849	Proximal
0	542	Female	White	4808	Proximal
0	1865	Male	White	3538	Proximal
0	1625	Female	White	5143	Proximal
0	1870	Female	Unknown	5931	Proximal
0	1702	Male	White	5667	Proximal
0	1575	Male	White	5755	Proximal
1	758	Male	White	4889	
0	1580	Female	White	4383	Distal
1	1579	Male	Unknown	5060	Proximal
0	1468	Male	White	5513	Proximal
0	1363	Female	White	4964	Proximal
0	1747	Male	White	5947	
0	1707	Female	White	1299	
0	1451	Male	White	5875	
1	510	Female	Unknown	6345	
0	1029	Female	White	4513	
0	687	Male	White	5064	
1	537	Female	White	4418	
0	672	Male	Unknown	2177	
1	347	Female	Black or African American	2027	
1	606	Female	Unknown	4507	
0	579	Female	White	6702	
0	551	Male	White	5924	
0	511	Female	Black or African American	4062	
0	369	Male	White	4898	
0	476	Female	White	3272	
1	386	Female	Unknown	7631	
0	3946	Male		7254	Proximal
0	377	Male	White	10205	Other, Not speci

0	1750	Male	White	10635	Distal
0	2948	Male	White	8470	Distal
1	290	Male		7264	Other, Posterior
0	1240	Female		18919	Distal
1	519	Female		7269	Distal
0	603	Male		9114	Other, Anterior
0	683	Male	Asian	7330	Distal
0	1844	Male	Black or African American	8313	Other, Posterior
1	619	Female		6268	Proximal
0	1616	Female		5854	Distal
		Male		5978	Proximal
0	708	Female	Asian	6916	Proximal
1	627	Male	White	11828	Distal
0	619	Male		7615	Proximal
1	1906	Male		6738	Distal
0	983	Male		7328	Distal
		Male	White	5496	
0	922	Male	Unknown	5407	Distal
0	2462	Female	White	5245	Proximal
1	1515	Male	White	4864	Distal
0	0	Male	Asian	7270	
1	754	Female	Unknown	3661	
1	1003	Female	White	4841	
0	4374	Male	Black or African American	6019	
0	3282	Female	White	3388	Distal
0	2122	Male	White	6190	
1	2882	Male	White	5603	Distal
0	1913	Male	White	3233	
0	3900	Female	Asian	3499	
1	3862	Male	Black or African American	4100	
0	3002	Female	Black or African American	4938	
0	3378	Male	White	4794	
0	3378	Male	White	6088	Distal
0	2256	Male	White	5234	Proximal
0	3095	Female	White	5044	
0	1217	Male	White	5545	Distal
0	1538	Male	White	6855	
1	78	Male	Black or African American	2803	
0	5840	Male		6520	PRoximal
1	2014	Male		32518	Distal
0	2257	Male		8237	Proximal

fied
