# Supplementary material for: Analysis of Immune Gene Expression Subtypes Reveals Osteosarcoma Immune Heterogeneity
Source: J Oncol. 2021 Mar 1;2021:6649412. doi: 10.1155/2021/6649412 (PMC7939746; doi:10.1155/2021/6649412)
Supplement: Supplementary Materials — Supplementary Figure S1. Consensus matrix for immune-related gene modules derived from consensus clustering analysis. Supplementary Figure S2. Expression feature of immune subtypes in training TARGET cohort. Supplementary Figure S3. The validation of immune subtypes in the independent GEO cohort. Supplementary Figure S4. The intracluster heterogeneity revealed by the immune landscape analysis and different subcluster molecular characteristics of patients in immune subtypes 1 and 4 are shown. Supplementary Table S1. The bulk RNA-seq data retrieved from the OS-project. Supplementary Table S2. The collected 1989 immune-related genes. Supplementary Table S3. The immune subtype annotation at the patient level in the TARGET cohort. Supplementary Table S4. The immune-related gene modules feature. Supplementary Table S5. Functional enrichment analysis of gene modules. Supplementary Table S6. The immune-related molecular features of the TARGET patients. Supplementary Table S7. Correlation of average gene module scores between molecular characteristics and immune subtypes. [file 6649412.f1.zip › 6649412.f1/Supplementary Table S2.pdf]

| GeneSymbol | GeneID |
|---|---|
| A2M | 2 |
| ABCA6 | 23460 |
| ABCA8 | 10351 |
| ABCA9 | 10350 |
| ABCB1 | 5243 |
| ABCC9 | 10060 |
| ABCD2 | 225 |
| ABI3 | 51225 |
| ABI3BP | 25890 |
| ACAP1 | 9744 |
| ACE | 1636 |
| ACHE | 43 |
| ACOXL | 55289 |
| ACP5 | 54 |
| ACSL5 | 51703 |
| ACSM5 | 54988 |
| ACSS3 | 79611 |
| ACTA2 | 59 |
| ACTN1 | 87 |
| ACVR1 | 90 |
| ACVR1B | 91 |
| ACVR2A | 92 |
| ACVR2B | 93 |
| ACVRL1 | 94 |
| ADAM12 | 8038 |
| ADAM28 | 10863 |
| ADAM6 | 8755 |
| ADAM8 | 101 |
| ADAMDEC1 | 27299 |
| ADAMTS10 | 81794 |
| ADAMTS12 | 81792 |
| ADAMTS14 | 140766 |
| ADAMTS16 | 170690 |
| ADAMTS2 | 9509 |
| ADAMTS4 | 9507 |
| ADAMTS5 | 11096 |
| ADAMTS9 | 56999 |
| ADAMTSL2 | 9719 |
| ADAP2 | 55803 |
| ADAT1 | 23536 |
| ADCY4 | 196883 |
| ADCYAP1 | 116 |
| ADORA2A | 135 |
| ADORA3 | 140 |
| ADPRH | 141 |
| ADRA2A | 150 |
| ADRM1 | 11047 |
| AEBP1 | 165 |
| AFF3 | 3899 |
| AGAP2 | 116986 |
| AGTR1 | 185 |

| | |
|---|---|
| AHCYL2 | 23382 |
| AIF1 | 199 |
| CRYBG2 | 55057 |
| AIM2 | 9447 |
| AKAP12 | 9590 |
| AKAP2 | 11217 |
| AKAP5 | 9495 |
| AKNA | 80709 |
| ALDH1A1 | 216 |
| ALDH3B1 | 221 |
| ALOX5 | 240 |
| ALOX5AP | 241 |
| ALOXE3 | 59344 |
| ALPK2 | 115701 |
| AMH | 268 |
| AMHR2 | 269 |
| JAML | 120425 |
| AMIGO3 | 386724 |
| AMPD1 | 270 |
| AMPH | 273 |
| ANGPTL1 | 9068 |
| ANGPTL2 | 23452 |
| ANK2 | 287 |
| ANKRD17 | 26057 |
| ANKRD22 | 118932 |
| ANKRD36BP1 | 84832 |
| ANKRD44 | 91526 |
| ANKRD55 | 79722 |
| SOWAHD | 347454 |
| ANO6 | 196527 |
| ANTXR1 | 84168 |
| ANTXR2 | 118429 |
| ANXA6 | 309 |
| AOAH | 313 |
| AOC3 | 8639 |
| AP1S2 | 8905 |
| AP3B1 | 8546 |
| APBB1IP | 54518 |
| APBB2 | 323 |
| APLNR | 187 |
| APOBR | 55911 |
| APOBEC3A | 200315 |
| APOBEC3D | 140564 |
| APOBEC3G | 60489 |
| APOBEC3H | 164668 |
| APOC1 | 341 |
| APOC2 | 344 |
| APOE | 348 |
| APOL3 | 80833 |
| APOL6 | 80830 |
| AQP10 | 89872 |
| AQP1 | 358 |

| | |
|---|---|
| AQP9 | 366 |
| AREG | 374 |
| ARHGAP15 | 55843 |
| ARHGAP18 | 93663 |
| ARHGAP22 | 58504 |
| ARHGAP25 | 9938 |
| ARHGAP30 | 257106 |
| ARHGAP31 | 57514 |
| ARHGAP4 | 393 |
| ARHGAP6 | 395 |
| ARHGAP9 | 64333 |
| ARHGDIB | 397 |
| ARHGEF15 | 22899 |
| ARHGEF37 | 389337 |
| ARHGEF6 | 9459 |
| ARID5A | 10865 |
| ARL6IP5 | 10550 |
| ARRB1 | 408 |
| ARRB2 | 409 |
| ARRDC5 | 645432 |
| ARSB | 411 |
| ART4 | 420 |
| ASAH1 | 427 |
| CLMP | 79827 |
| ASGR2 | 433 |
| ASPN | 54829 |
| ASRGL1 | 80150 |
| ASXL2 | 55252 |
| ASXL3 | 80816 |
| ATE1 | 11101 |
| ATP10A | 57194 |
| ATP2A3 | 489 |
| ATP8A1 | 10396 |
| ATP8B4 | 79895 |
| AVPR1A | 552 |
| AZGP1 | 563 |
| B2M | 567 |
| B3GAT1 | 27087 |
| BANK1 | 55024 |
| BATF | 10538 |
| BATF2 | 116071 |
| BATF3 | 55509 |
| BCL2A1 | 597 |
| BCL2L14 | 79370 |
| BCL6B | 255877 |
| BDKRB2 | 624 |
| BEND5 | 79656 |
| BEX5 | 340542 |
| BFSP2 | 8419 |
| BGN | 633 |
| BHLHA15 | 168620 |
| BHLHE22 | 27319 |

| | |
|---|---|
| BHLHE41 | 79365 |
| BICC1 | 80114 |
| BIN2 | 51411 |
| BIRC6 | 57448 |
| BLK | 640 |
| BMP2 | 650 |
| BMP2K | 55589 |
| BMP7 | 655 |
| BMPR1A | 657 |
| BMPR1B | 658 |
| BMPR2 | 659 |
| BNC2 | 54796 |
| BST1 | 683 |
| BST2 | 684 |
| BTK | 695 |
| BTLA | 151888 |
| BTN2A2 | 10385 |
| BTN3A1 | 11119 |
| BTN3A2 | 11118 |
| BTN3A3 | 10384 |
| C10orf128 | 170371 |
| VSIR | 64115 |
| VSTM4 | 196740 |
| C10orf99 | 387695 |
| C11orf21 | 29125 |
| RUBCNL | 80183 |
| MEDAG | 84935 |
| CEP128 | 145508 |
| SLIRP | 81892 |
| RTRAF | 51637 |
| C14orf2 | 9556 |
| C15orf48 | 84419 |
| C15orf53 | 400359 |
| C16orf54 | 283897 |
| MILR1 | 284021 |
| SCIMP | 388325 |
| LDLRAD4 | 753 |
| PEAK3 | 374872 |
| C19orf38 | 255809 |
| TRIR | 79002 |
| MCEMP1 | 199675 |
| C19orf66 | 55337 |
| C1orf116 | 79098 |
| C1orf127 | 148345 |
| GCSAML | 148823 |
| C1orf162 | 128346 |
| PIK3CD-AS1 | 644997 |
| C1orf228 | 339541 |
| THEMIS2 | 9473 |
| C1orf54 | 79630 |
| C1QA | 712 |
| C1QB | 713 |

| | |
|---|---|
| C1QC | 714 |
| C1QTNF7 | 114905 |
| LAMP5 | 24141 |
| C2 | 717 |
| RTP5 | 285093 |
| TRABD2A | 129293 |
| C3 | 718 |
| C3AR1 | 719 |
| C4A | 720 |
| NDNF | 79625 |
| C5AR1 | 728 |
| DCANP1 | 140947 |
| CREBRF | 153222 |
| C5orf56 | 441108 |
| ADTRP | 84830 |
| UQCC2 | 84300 |
| C6orf132 | 647024 |
| CCDC170 | 80129 |
| C7 | 730 |
| CPED1 | 79974 |
| NUGGC | 389643 |
| ERCC6L2 | 375748 |
| C9orf139 | 401563 |
| CYSRT1 | 375791 |
| CACNA1C | 775 |
| CACNA2D2 | 9254 |
| CACNA2D4 | 93589 |
| CALB2 | 794 |
| CALD1 | 800 |
| CALR | 811 |
| CAMK1 | 8536 |
| CAMK4 | 814 |
| CANX | 821 |
| CARD11 | 84433 |
| CARD8 | 22900 |
| CARD9 | 64170 |
| CASP5 | 838 |
| CASS4 | 57091 |
| CAV1 | 857 |
| CCDC102B | 79839 |
| CCDC141 | 285025 |
| CCDC69 | 26112 |
| CCDC80 | 151887 |
| CCL11 | 6356 |
| CCL13 | 6357 |
| CCL14 | 6358 |
| CCL15 | 6359 |
| CCL1 | 6346 |
| CCL16 | 6360 |
| CCL17 | 6361 |
| CCL18 | 6362 |
| CCL19 | 6363 |

| | |
|---|---|
| CCL20 | 6364 |
| CCL21 | 6366 |
| CCL22 | 6367 |
| CCL23 | 6368 |
| CCL24 | 6369 |
| CCL25 | 6370 |
| CCL26 | 10344 |
| CCL2 | 6347 |
| CCL28 | 56477 |
| CCL3 | 6348 |
| CCL3L1 | 6349 |
| CCL3L3 | 414062 |
| CCL4 | 6351 |
| CCL4L1 | 388372 |
| CCL5 | 6352 |
| CCL7 | 6354 |
| CCL8 | 6355 |
| CCNT1 | 904 |
| CCR10 | 2826 |
| CCR1 | 1230 |
| CCR2 | 729230 |
| CCR3 | 1232 |
| CCR4 | 1233 |
| CCR5 | 1234 |
| CCR6 | 1235 |
| CCR7 | 1236 |
| CCR8 | 1237 |
| CCR9 | 10803 |
| CCRL2 | 9034 |
| CD14 | 929 |
| CD160 | 11126 |
| CD163 | 9332 |
| CD163L1 | 283316 |
| CD180 | 4064 |
| CD19 | 930 |
| CD1A | 909 |
| CD1B | 910 |
| CD1C | 911 |
| CD1D | 912 |
| CD1E | 913 |
| CD200 | 4345 |
| CD200R1 | 131450 |
| CD207 | 50489 |
| CD209 | 30835 |
| CD226 | 10666 |
| CD22 | 933 |
| CD244 | 51744 |
| CD247 | 919 |
| CD248 | 57124 |
| CD274 | 29126 |
| CD27 | 939 |
| CD28 | 940 |

| | |
|---|---|
| CD2 | 914 |
| CD300A | 11314 |
| CD300C | 10871 |
| CD300E | 342510 |
| CD300LB | 124599 |
| CD300LF | 146722 |
| CD302 | 9936 |
| CD33 | 945 |
| CD34 | 947 |
| CD36 | 948 |
| CD37 | 951 |
| CD3D | 915 |
| CD3E | 916 |
| CD3G | 917 |
| CD40 | 958 |
| CD40LG | 959 |
| CD48 | 962 |
| CD4 | 920 |
| CD52 | 1043 |
| CD53 | 963 |
| CD58 | 965 |
| CD5 | 921 |
| CD68 | 968 |
| CD6 | 923 |
| CD69 | 969 |
| CD70 | 970 |
| CD72 | 971 |
| CD74 | 972 |
| CD7 | 924 |
| CD79A | 973 |
| CD79B | 974 |
| CD80 | 941 |
| CD84 | 8832 |
| CD86 | 942 |
| CD8A | 925 |
| CD8B | 926 |
| CD93 | 22918 |
| CD96 | 10225 |
| ADGRE5 | 976 |
| CDC42SE2 | 56990 |
| CDH11 | 1009 |
| CDH20 | 28316 |
| CDH3 | 1001 |
| CDH5 | 1003 |
| CDH6 | 1004 |
| CDK15 | 65061 |
| CDKL5 | 6792 |
| CDSN | 1041 |
| CEACAM19 | 56971 |
| CEACAM21 | 90273 |
| CEACAM4 | 1089 |
| ADA2 | 51816 |

| | |
|---|---|
| CELF2 | 10659 |
| CERKL | 375298 |
| CETP | 1071 |
| CFP | 5199 |
| CHAC1 | 79094 |
| CHIT1 | 1118 |
| CHN1 | 1123 |
| CHRD | 8646 |
| CHRDL1 | 91851 |
| CHRNA6 | 8973 |
| CHST13 | 166012 |
| CHST2 | 9435 |
| CIITA | 4261 |
| CILP | 8483 |
| CISH | 1154 |
| CLCF1 | 23529 |
| CLEC10A | 10462 |
| CLEC11A | 6320 |
| CLEC12A | 160364 |
| CLEC14A | 161198 |
| CLEC1A | 51267 |
| CLEC3B | 7123 |
| CLEC4A | 50856 |
| CLEC4D | 338339 |
| CLEC4E | 26253 |
| CLEC4G | 339390 |
| CLEC4M | 10332 |
| CLEC5A | 23601 |
| CLEC6A | 93978 |
| CLEC9A | 283420 |
| CLECL1 | 160365 |
| CLIC2 | 1193 |
| CLIC3 | 9022 |
| CLIC5 | 53405 |
| CLIP3 | 25999 |
| CLNK | 116449 |
| CLOCK | 9575 |
| CLTB | 1212 |
| CMA1 | 1215 |
| CMAHP | 8418 |
| CMKLR1 | 1240 |
| CMPK2 | 129607 |
| CNFN | 84518 |
| CNR2 | 1269 |
| CNRIP1 | 25927 |
| CNTF | 1270 |
| CNTFR | 1271 |
| COL10A1 | 1300 |
| COL11A1 | 1301 |
| COL12A1 | 1303 |
| COL14A1 | 7373 |
| COL15A1 | 1306 |

| | |
|---|---|
| COL18A1 | 80781 |
| COL1A1 | 1277 |
| COL1A2 | 1278 |
| COL6A5 | 256076 |
| COL3A1 | 1281 |
| COL4A1 | 1282 |
| COL4A2 | 1284 |
| COL5A1 | 1289 |
| COL5A2 | 1290 |
| COL5A3 | 50509 |
| COL6A1 | 1291 |
| COL6A2 | 1292 |
| COL6A3 | 1293 |
| COL6A6 | 131873 |
| COL8A1 | 1295 |
| COLEC12 | 81035 |
| CORIN | 10699 |
| CORO1A | 11151 |
| COTL1 | 23406 |
| CPA3 | 1359 |
| CPNE5 | 57699 |
| CPVL | 54504 |
| CPXM1 | 56265 |
| CPZ | 8532 |
| CR1 | 1378 |
| CR1L | 1379 |
| CR2 | 1380 |
| CRCT1 | 54544 |
| CREB1 | 1385 |
| CREB3L1 | 90993 |
| CREBL2 | 1389 |
| CRISPLD2 | 83716 |
| CRLF2 | 64109 |
| CRTAM | 56253 |
| CRYBB1 | 1414 |
| CSF1 | 1435 |
| CSF1R | 1436 |
| CSF2 | 1437 |
| CSF2RA | 1438 |
| CSF2RB | 1439 |
| CSF3 | 1440 |
| CSF3R | 1441 |
| CSGALNACT2 | 55454 |
| CSMD2 | 114784 |
| CST7 | 8530 |
| CTF1 | 1489 |
| CTGF | 1490 |
| CTHRC1 | 115908 |
| CTLA4 | 1493 |
| CTSB | 1508 |
| CTSE | 1510 |
| CTSG | 1511 |

| | |
|---|---|
| CTSK | 1513 |
| CTSL | 1514 |
| CTSO | 1519 |
| CTSS | 1520 |
| CTSW | 1521 |
| CTSZ | 1522 |
| CTTNBP2 | 83992 |
| CX3CL1 | 6376 |
| CX3CR1 | 1524 |
| CXCL10 | 3627 |
| CXCL11 | 6373 |
| CXCL12 | 6387 |
| CXCL1 | 2919 |
| CXCL13 | 10563 |
| CXCL16 | 58191 |
| CXCL2 | 2920 |
| CXCL3 | 2921 |
| CXCL5 | 6374 |
| CXCL6 | 6372 |
| CXCL9 | 4283 |
| CXCR1 | 3577 |
| CXCR2 | 3579 |
| CXCR2P1 | 3580 |
| CXCR3 | 2833 |
| CXCR4 | 7852 |
| CXCR5 | 643 |
| CXCR6 | 10663 |
| ACKR3 | 57007 |
| CXorf21 | 80231 |
| CXorf36 | 79742 |
| CXorf65 | 158830 |
| CYBA | 1535 |
| CYBB | 1536 |
| CYFIP2 | 26999 |
| CYP1B1 | 1545 |
| CYP27A1 | 1593 |
| CYSLTR1 | 10800 |
| CYSLTR2 | 57105 |
| CYTH4 | 27128 |
| CYTIP | 9595 |
| DAAM2 | 23500 |
| DAB2 | 1601 |
| DACT1 | 51339 |
| DACT3 | 147906 |
| ACKR1 | 2532 |
| DBH | 1621 |
| DCBLD1 | 285761 |
| DCHS1 | 8642 |
| DCN | 1634 |
| DDI2 | 84301 |
| DDR2 | 4921 |
| DDX58 | 23586 |

| | |
|---|---|
| DDX60 | 55601 |
| DENND1C | 79958 |
| DENND2A | 27147 |
| DERL3 | 91319 |
| DHRS1 | 115817 |
| DHRS9 | 10170 |
| DIXDC1 | 85458 |
| DKK2 | 27123 |
| DLC1 | 10395 |
| DLL4 | 54567 |
| DMKN | 93099 |
| DMXL2 | 23312 |
| DNAH8 | 1769 |
| DNAJC5B | 85479 |
| DOCK10 | 55619 |
| DOCK11 | 139818 |
| DOCK2 | 1794 |
| DOCK4 | 9732 |
| DOCK8 | 81704 |
| DOK1 | 1796 |
| DOK2 | 9046 |
| DOK3 | 79930 |
| DOK5 | 55816 |
| DOK6 | 220164 |
| DPEP1 | 1800 |
| DPEP2 | 64174 |
| DPP8 | 54878 |
| DPT | 1805 |
| DSC1 | 1823 |
| DSG1 | 1828 |
| DSP | 1832 |
| DUOXA1 | 90527 |
| DUSP16 | 80824 |
| DUSP4 | 1846 |
| DYSF | 8291 |
| E2F5 | 1875 |
| EBF1 | 1879 |
| EBF2 | 64641 |
| EBI3 | 10148 |
| ECM2 | 1842 |
| ECSCR | 641700 |
| EDA | 1896 |
| EDA2R | 60401 |
| EDAR | 10913 |
| EDNRA | 1909 |
| EDNRB | 1910 |
| EFEMP2 | 30008 |
| EFTUD2 | 9343 |
| EGF | 1950 |
| EGFR | 1956 |
| EHD2 | 30846 |
| ADGRL4 | 64123 |

| | |
|---|---|
| EMCN | 51705 |
| EMILIN1 | 11117 |
| EMILIN2 | 84034 |
| ADGRE2 | 30817 |
| ADGRE4P | 326342 |
| ENG | 2022 |
| ENO3 | 2027 |
| ENOX1 | 55068 |
| ENPEP | 2028 |
| ENPP2 | 5168 |
| ENPP3 | 5169 |
| ENPP4 | 22875 |
| ENTPD1 | 953 |
| EOMES | 8320 |
| EP300 | 2033 |
| EPCAM | 4072 |
| EPO | 2056 |
| EPOR | 2057 |
| EPS8 | 2059 |
| EPS8L1 | 54869 |
| EPSTI1 | 94240 |
| ERAP1 | 51752 |
| ERAP2 | 64167 |
| ERN1 | 2081 |
| ERP27 | 121506 |
| ESAM | 90952 |
| ETS1 | 2113 |
| ETV3 | 2117 |
| ETV7 | 51513 |
| EVI2A | 2123 |
| EVI2B | 2124 |
| EVPL | 2125 |
| F13A1 | 2162 |
| F2R | 2149 |
| F5 | 2153 |
| FABP3 | 2170 |
| FCMR | 9214 |
| FAM105A | 54491 |
| FAM107A | 11170 |
| PCED1B | 91523 |
| DENND6B | 414918 |
| FAM129C | 199786 |
| FAM13C | 220965 |
| FAM155A | 728215 |
| FAM168A | 23201 |
| FAM171B | 165215 |
| FAM177B | 400823 |
| TVP23A | 780776 |
| CCSER1 | 401145 |
| FAM198B | 51313 |
| FAM19A5 | 25817 |
| FAM25A | 643161 |

| | |
|---|---|
| FAM25BP | 100132929 |
| CALHM5 | 254228 |
| CALHM6 | 441168 |
| PIEZO2 | 63895 |
| STRIP2 | 57464 |
| FAM46B | 115572 |
| FAM46C | 54855 |
| FAM49A | 81553 |
| NXPE4 | 54827 |
| MINDY2 | 54629 |
| RIPOR2 | 9750 |
| FAM78A | 286336 |
| FAM83A | 84985 |
| FAM92B | 339145 |
| FAP | 2191 |
| FAS | 355 |
| FASLG | 356 |
| FAT4 | 79633 |
| FBLN2 | 2199 |
| FBLN5 | 10516 |
| FBN1 | 2200 |
| FBP1 | 2203 |
| FBXL7 | 23194 |
| FBXO6 | 26270 |
| FCAR | 2204 |
| FCER1A | 2205 |
| FCER1G | 2207 |
| FCER2 | 2208 |
| FCGBP | 8857 |
| FCGR1A | 2209 |
| FCGR1B | 2210 |
| FCGR1CP | 100132417 |
| FCGR2A | 2212 |
| FCGR2B | 2213 |
| FCGR2C | 9103 |
| FCGR3A | 2214 |
| FCGR3B | 2215 |
| FCGRT | 2217 |
| FCN1 | 2219 |
| FCRL1 | 115350 |
| FCRL2 | 79368 |
| FCRL3 | 115352 |
| FCRL4 | 83417 |
| FCRL5 | 83416 |
| FCRL6 | 343413 |
| FCRLA | 84824 |
| FERMT2 | 10979 |
| FERMT3 | 83706 |
| FGD2 | 221472 |
| FGD3 | 89846 |
| FGD5 | 152273 |
| FGF14 | 2259 |

| | |
|---|---|
| FGF7 | 2252 |
| FGL2 | 10875 |
| FGR | 2268 |
| FHL5 | 9457 |
| FIBIN | 387758 |
| FICD | 11153 |
| VEGFD | 2277 |
| FILIP1L | 11259 |
| FKBP11 | 51303 |
| FKBP7 | 51661 |
| FLI1 | 2313 |
| ANKRD36BP2 | 645784 |
| FLT1 | 2321 |
| FLT3 | 2322 |
| FLT3LG | 2323 |
| FLT4 | 2324 |
| FLVCR2 | 55640 |
| FMNL1 | 752 |
| FMNL3 | 91010 |
| FMOD | 2331 |
| FN1 | 2335 |
| FNBP1 | 23048 |
| FNDC1 | 84624 |
| FNIP2 | 57600 |
| FOLR2 | 2350 |
| FOXP3 | 50943 |
| FPR1 | 2357 |
| FPR2 | 2358 |
| FPR3 | 2359 |
| FRZB | 2487 |
| FSCN1 | 6624 |
| FSTL1 | 11167 |
| FSTL3 | 10272 |
| FUCA1 | 2517 |
| FUT7 | 2529 |
| FYB1 | 2533 |
| FYN | 2534 |
| FZD4 | 8322 |
| GAB3 | 139716 |
| GALM | 130589 |
| GALNT15 | 117248 |
| GAPT | 202309 |
| GAS7 | 8522 |
| GATA1 | 2623 |
| GATA2 | 2624 |
| GATA3 | 2625 |
| GATM | 2628 |
| GBGT1 | 26301 |
| GBP1 | 2633 |
| GBP2 | 2634 |
| GBP4 | 115361 |
| GBP5 | 115362 |

| | |
|---|---|
| GCSAM | 257144 |
| GDF5 | 8200 |
| GFI1 | 2672 |
| GFRA3 | 2676 |
| GGT1 | 2678 |
| GGT5 | 2687 |
| GGTA1P | 2681 |
| GHR | 2690 |
| GHRL | 51738 |
| GIMAP1 | 170575 |
| GIMAP2 | 26157 |
| GIMAP4 | 55303 |
| GIMAP5 | 55340 |
| GIMAP6 | 474344 |
| GIMAP7 | 168537 |
| GIMAP8 | 155038 |
| GIPC3 | 126326 |
| GIT2 | 9815 |
| GJA4 | 2701 |
| GJA5 | 2702 |
| GJB2 | 2706 |
| GJB3 | 2707 |
| GJB5 | 2709 |
| GJD3 | 125111 |
| GLIPR2 | 152007 |
| GLIS3 | 169792 |
| GLRX | 2745 |
| COLGALT2 | 23127 |
| GLT8D2 | 83468 |
| GMFG | 9535 |
| GMIP | 51291 |
| GMPR | 2766 |
| GNA15 | 2769 |
| GNAI2 | 2771 |
| GNG11 | 2791 |
| GNG2 | 54331 |
| GNG7 | 2788 |
| GNGT2 | 2793 |
| GNLY | 10578 |
| GNS | 2799 |
| GPBAR1 | 151306 |
| GPC5 | 2262 |
| GPC6 | 10082 |
| GPIHBP1 | 338328 |
| ADGRG5 | 221188 |
| ADGRF4 | 221393 |
| ADGRF5 | 221395 |
| ADGRA2 | 25960 |
| GPR132 | 29933 |
| ADGRD1 | 283383 |
| GPR137B | 7107 |
| GPR141 | 353345 |

| | |
|---|---|
| GPR15 | 2838 |
| GPR157 | 80045 |
| GPR171 | 29909 |
| GPR174 | 84636 |
| GPR18 | 2841 |
| GPR183 | 1880 |
| GPR25 | 2848 |
| GPR34 | 2857 |
| GPR35 | 2859 |
| GPR4 | 2828 |
| GPR55 | 9290 |
| GPR65 | 8477 |
| GPR78 | 27201 |
| GPR82 | 27197 |
| GPR84 | 53831 |
| GPRIN3 | 285513 |
| GPSM3 | 63940 |
| GRAP | 10750 |
| GRAP2 | 9402 |
| GREM1 | 26585 |
| GRIN3A | 116443 |
| GSDMA | 284110 |
| GTF2A1 | 2957 |
| GUCY1A2 | 2977 |
| GUCY1A3 | 2982 |
| GVINP1 | 387751 |
| GYPC | 2995 |
| GZMA | 3001 |
| GZMB | 3002 |
| GZMH | 2999 |
| GZMK | 3003 |
| GZMM | 3004 |
| HAMP | 57817 |
| HAPLN3 | 145864 |
| HAVCR1 | 26762 |
| HAVCR2 | 84868 |
| HCG26 | 352961 |
| HCK | 3055 |
| HCLS1 | 3059 |
| HCP5 | 10866 |
| HCST | 10870 |
| HDC | 3067 |
| HECW2 | 57520 |
| HEPH | 9843 |
| HEPHL1 | 341208 |
| HERC6 | 55008 |
| HERPUD1 | 9709 |
| HEYL | 26508 |
| HFE | 3077 |
| HGF | 3082 |
| HIC1 | 3090 |
| HIPK3 | 10114 |

| | |
|---|---|
| HIST1H2AE | 3012 |
| HIST1H2AG | 8969 |
| HIST1H2AM | 8336 |
| HIST1H3H | 8357 |
| HK3 | 3101 |
| HLA-A | 3105 |
| HLA-B | 3106 |
| HLA-C | 3107 |
| HLA-DMA | 3108 |
| HLA-DMB | 3109 |
| HLA-DOA | 3111 |
| HLA-DOB | 3112 |
| HLA-DPA1 | 3113 |
| HLA-DPB1 | 3115 |
| HLA-DPB2 | 3116 |
| HLA-DQA1 | 3117 |
| HLA-DQA2 | 3118 |
| HLA-DQB1 | 3119 |
| HLA-DQB2 | 3120 |
| HLA-DRA | 3122 |
| HLA-DRB1 | 3123 |
| HLA-DRB5 | 3127 |
| HLA-DRB6 | 3128 |
| HLA-E | 3133 |
| HLA-F | 3134 |
| HLA-G | 3135 |
| HLA-H | 3136 |
| HLX | 3142 |
| HMCN1 | 83872 |
| ARHGAP45 | 23526 |
| HMSD | 284293 |
| HNMT | 3176 |
| HPGD | 3248 |
| HPGDS | 27306 |
| HRH2 | 3274 |
| HS3ST1 | 9957 |
| HS3ST2 | 9956 |
| HSD11B1 | 3290 |
| HSD17B14 | 51171 |
| HSH2D | 84941 |
| HSP90AA1 | 3320 |
| HSP90AB1 | 3326 |
| HSPA12B | 116835 |
| HSPA1A | 3303 |
| HSPA1B | 3304 |
| HSPA1L | 3305 |
| HSPA2 | 3306 |
| HSPA4 | 3308 |
| HSPA5 | 3309 |
| HSPA6 | 3310 |
| HSPA8 | 3312 |
| HTR2A | 3356 |

| | |
|---|---|
| HTRA3 | 94031 |
| HTRA4 | 203100 |
| HVCN1 | 84329 |
| HYDIN | 54768 |
| ICAM1 | 3383 |
| ICAM2 | 3384 |
| ICAM3 | 3385 |
| ICK | 22858 |
| ICOS | 29851 |
| ICOSLG | 23308 |
| IDO1 | 3620 |
| IDO2 | 169355 |
| IFFO1 | 25900 |
| IFI27 | 3429 |
| IFI30 | 10437 |
| IFI35 | 3430 |
| IFI44 | 10561 |
| IFI44L | 10964 |
| IFI6 | 2537 |
| IFIH1 | 64135 |
| IFIT2 | 3433 |
| IFIT3 | 3437 |
| IFIT5 | 24138 |
| IFITM1 | 8519 |
| IFITM3 | 10410 |
| IFNA13 | 3447 |
| IFNA21 | 3452 |
| IFNAR1 | 3454 |
| IFNAR2 | 3455 |
| IFNB1 | 3456 |
| IFNE | 338376 |
| IFNG | 3458 |
| IFNGR1 | 3459 |
| IFNGR2 | 3460 |
| IFNK | 56832 |
| IFNW1 | 3467 |
| IGDCC4 | 57722 |
| IGF1 | 3479 |
| JCHAIN | 3512 |
| IGLL1 | 3543 |
| IGSF10 | 285313 |
| IGSF21 | 84966 |
| IGSF6 | 10261 |
| IKZF1 | 10320 |
| IKZF3 | 22806 |
| IL10 | 3586 |
| IL10RA | 3587 |
| IL10RB | 3588 |
| IL11 | 3589 |
| IL11RA | 3590 |
| IL12A | 3592 |
| IL12B | 3593 |

| | |
|---|---|
| IL12RB1 | 3594 |
| IL12RB2 | 3595 |
| IL13 | 3596 |
| IL13RA1 | 3597 |
| IL15 | 3600 |
| IL15RA | 3601 |
| IL16 | 3603 |
| IL17A | 3605 |
| IL17B | 27190 |
| IL17RA | 23765 |
| IL17RB | 55540 |
| IL18 | 3606 |
| IL18BP | 10068 |
| IL18R1 | 8809 |
| IL18RAP | 8807 |
| IL19 | 29949 |
| IL1A | 3552 |
| IL1B | 3553 |
| IL36G | 56300 |
| IL1R1 | 3554 |
| IL1R2 | 7850 |
| IL1RAP | 3556 |
| IL1RL1 | 9173 |
| IL1RN | 3557 |
| IL20 | 50604 |
| IL20RA | 53832 |
| IL20RB | 53833 |
| IL21 | 59067 |
| IL21R | 50615 |
| IL22 | 50616 |
| IL22RA1 | 58985 |
| IL22RA2 | 116379 |
| IL2 | 3558 |
| IL23A | 51561 |
| IL23R | 149233 |
| IL24 | 11009 |
| IL27 | 246778 |
| IFNL2 | 282616 |
| IFNLR1 | 163702 |
| IFNL1 | 282618 |
| IL2RA | 3559 |
| IL2RB | 3560 |
| IL2RG | 3561 |
| IL32 | 9235 |
| IL3RA | 3563 |
| IL4 | 3565 |
| IL4I1 | 259307 |
| IL4R | 3566 |
| IL5 | 3567 |
| IL5RA | 3568 |
| IL6 | 3569 |
| IL6R | 3570 |

| | |
|---|---|
| IL6ST | 3572 |
| IL7 | 3574 |
| IL7R | 3575 |
| CXCL8 | 3576 |
| IL9 | 3578 |
| IL9R | 3581 |
| INHBA | 3624 |
| INHBB | 3625 |
| INHBC | 3626 |
| INHBE | 83729 |
| INMT | 11185 |
| INPP5D | 3635 |
| IPCEF1 | 26034 |
| IQGAP2 | 10788 |
| IRF1 | 3659 |
| IRF4 | 3662 |
| IRF7 | 3665 |
| IRF8 | 3394 |
| IRF9 | 10379 |
| ISG15 | 9636 |
| ISG20 | 3669 |
| ISLR | 3671 |
| ITGA11 | 22801 |
| ITGA1 | 3672 |
| ITGA2B | 3674 |
| ITGA4 | 3676 |
| ITGA5 | 3678 |
| ITGA8 | 8516 |
| ITGA9 | 3680 |
| ITGAD | 3681 |
| ITGAL | 3683 |
| ITGAM | 3684 |
| ITGAX | 3687 |
| ITGB1 | 3688 |
| ITGB2 | 3689 |
| ITGB3 | 3690 |
| ITGB7 | 3695 |
| ITGBL1 | 9358 |
| ITK | 3702 |
| ITM2A | 9452 |
| IVL | 3713 |
| JAK2 | 3717 |
| JAK3 | 3718 |
| JAKMIP1 | 152789 |
| JAM2 | 58494 |
| JAM3 | 83700 |
| JMY | 133746 |
| JSRP1 | 126306 |
| JUP | 3728 |
| KCNA3 | 3738 |
| KCNAB2 | 8514 |
| KCND2 | 3751 |

| | |
|---|---|
| KCNE4 | 23704 |
| KCNH2 | 3757 |
| KCNJ10 | 3766 |
| KCNJ8 | 3764 |
| KCNK13 | 56659 |
| KCNK6 | 9424 |
| KCNMB1 | 3779 |
| KCNN3 | 3782 |
| KCNN4 | 3783 |
| KCNT2 | 343450 |
| KCTD12 | 115207 |
| KDR | 3791 |
| FAM30A | 9834 |
| KIAA0368 | 23392 |
| TESPA1 | 9840 |
| KIAA0754 | 643314 |
| JCAD | 57608 |
| KIAA1549 | 57670 |
| TLDC1 | 57707 |
| SHISAL1 | 85352 |
| KIAA1755 | 85449 |
| KIF21B | 23046 |
| KIR2DL1 | 3802 |
| KIR2DL3 | 3804 |
| KIR2DL4 | 3805 |
| KIR2DS4 | 3809 |
| KIR3DL1 | 3811 |
| KIR3DL2 | 3812 |
| KIR3DL3 | 115653 |
| KIRREL1 | 55243 |
| KIT | 3815 |
| KITLG | 4254 |
| KL | 9365 |
| KLHDC10 | 23008 |
| KLHL11 | 55175 |
| KLHL23 | 151230 |
| KLHL6 | 89857 |
| KLK7 | 5650 |
| KLK9 | 284366 |
| KLRB1 | 3820 |
| KLRC1 | 3821 |
| KLRC2 | 3822 |
| KLRC3 | 3823 |
| KLRC4 | 8302 |
| KLRD1 | 3824 |
| KLRG1 | 10219 |
| KLRK1 | 22914 |
| KMO | 8564 |
| KRT1 | 3848 |
| KRT14 | 3861 |
| KRT16 | 3868 |
| KRT6A | 3853 |

| | |
|---|---|
| KRT6B | 3854 |
| KRT6C | 286887 |
| KRT78 | 196374 |
| LAD1 | 3898 |
| LAG3 | 3902 |
| LAIR1 | 3903 |
| LAIR2 | 3904 |
| LAMA2 | 3908 |
| LAMA4 | 3910 |
| LAMC2 | 3918 |
| LAP3 | 51056 |
| LAPTM5 | 7805 |
| LAT | 27040 |
| LAT2 | 7462 |
| LATS1 | 9113 |
| LATS2 | 26524 |
| LAX1 | 54900 |
| LCE3D | 84648 |
| LCK | 3932 |
| LCN10 | 414332 |
| LCOR | 84458 |
| LCP1 | 3936 |
| LCP2 | 3937 |
| LDB2 | 9079 |
| LEP | 3952 |
| LEPR | 3953 |
| LGALS2 | 3957 |
| LGALS9 | 3965 |
| LGI2 | 55203 |
| LGMN | 5641 |
| LHFPL6 | 10186 |
| LHFPL2 | 10184 |
| LIF | 3976 |
| LIFR | 3977 |
| LIG3 | 3980 |
| LILRA1 | 11024 |
| LILRA2 | 11027 |
| LILRA3 | 11026 |
| LILRA4 | 23547 |
| LILRA5 | 353514 |
| LILRA6 | 79168 |
| LILRB1 | 10859 |
| LILRB2 | 10288 |
| LILRB3 | 11025 |
| LILRB4 | 11006 |
| LILRB5 | 10990 |
| LILRP2 | 79166 |
| LIMD2 | 80774 |
| LIME1 | 54923 |
| LIMS1 | 3987 |
| LIPA | 3988 |
| LMOD1 | 25802 |

| | |
|---|---|
| LMTK2 | 22853 |
| UNQ6494 | 100129066 |
| FAM83A-AS1 | 100131726 |
| LINC00426 | 100188949 |
| PCED1B-AS1 | 100233209 |
| TNFRSF14-AS1 | 115110 |
| LINC00654 | 149837 |
| LINC00926 | 283663 |
| SMIM1 | 388588 |
| LGALS17A | 400696 |
| MIR31HG | 554202 |
| LOC606724 | 606724 |
| LOC653786 | 653786 |
| LOC730101 | 730101 |
| BMS1P20 | 96610 |
| LOXL2 | 4017 |
| LOXL3 | 84695 |
| LPAR4 | 2846 |
| LPL | 4023 |
| PLPPR4 | 9890 |
| LPXN | 9404 |
| LRCH2 | 57631 |
| LRMP | 4033 |
| LRP6 | 4040 |
| LRRC15 | 131578 |
| LRRC17 | 10234 |
| LRRC25 | 126364 |
| LRRC32 | 2615 |
| NRROS | 375387 |
| LSAMP | 4045 |
| LSP1 | 4046 |
| LST1 | 7940 |
| LTA | 4049 |
| LTB | 4050 |
| LTBP2 | 4053 |
| LTBR | 4055 |
| LTC4S | 4056 |
| LUM | 4060 |
| LY86 | 9450 |
| LY9 | 4063 |
| LY96 | 23643 |
| LYL1 | 4066 |
| LYN | 4067 |
| LYPD3 | 27076 |
| LYPD5 | 284348 |
| LYVE1 | 10894 |
| LYZ | 4069 |
| MAGEL2 | 54551 |
| MAN1A1 | 4121 |
| MAN1A2 | 10905 |
| MAN1C1 | 57134 |
| MAOB | 4129 |

| | | |
|---|---|---|
| MAP1LC3C | | 440738 |
| MAP3K2 | | 10746 |
| MAP4K1 | | 11184 |
| MAP7D1 | | 55700 |
| | 43160 | 55016 |
| | 43167 | 220972 |
| MARCO | | 8685 |
| MBNL3 | | 55796 |
| SLC25A53 | | 401612 |
| MCOLN2 | | 255231 |
| MED13 | | 9969 |
| MED13L | | 23389 |
| MEF2B | | 100271849 |
| MEF2C | | 4208 |
| MEI1 | | 150365 |
| MEOX2 | | 4223 |
| MET | | 4233 |
| MFAP3 | | 4238 |
| MFAP4 | | 4239 |
| MFNG | | 4242 |
| MFRP | | 83552 |
| MFSD7 | | 84179 |
| MGAT4A | | 11320 |
| MGAT5 | | 4249 |
| MZB1 | | 51237 |
| MGP | | 4256 |
| NA | | 4276 |
| MICAL2 | | 9645 |
| MICB | | 4277 |
| MIR155HG | | 114614 |
| MITF | | 4286 |
| MLPH | | 79083 |
| MMP12 | | 4321 |
| MMP1 | | 4312 |
| MMP14 | | 4323 |
| MMP16 | | 4325 |
| MMP2 | | 4313 |
| MMP25 | | 64386 |
| MMP3 | | 4314 |
| MMP9 | | 4318 |
| MMRN1 | | 22915 |
| MMRN2 | | 79812 |
| MNDA | | 4332 |
| MPEG1 | | 219972 |
| MPL | | 4352 |
| MPP1 | | 4354 |
| MR1 | | 3140 |
| MRC1 | | 4360 |
| MRGPRF | | 116535 |
| MRO | | 83876 |
| MRPL27 | | 51264 |
| MRPL55 | | 128308 |

| | |
|---|---|
| MRPS12 | 6183 |
| MRPS21 | 54460 |
| MRVI1 | 10335 |
| MS4A14 | 84689 |
| MS4A1 | 931 |
| MS4A2 | 2206 |
| MS4A4A | 51338 |
| MS4A6A | 64231 |
| MS4A7 | 58475 |
| MSR1 | 4481 |
| MSRB3 | 253827 |
| MVP | 9961 |
| MX1 | 4599 |
| MXD1 | 4084 |
| MXRA8 | 54587 |
| MYCT1 | 80177 |
| MYEF2 | 50804 |
| MYO1F | 4542 |
| MYO1G | 64005 |
| MYO7A | 4647 |
| MYO9A | 4649 |
| N4BP2 | 55728 |
| N4BP2L1 | 90634 |
| NAALADL1 | 10004 |
| NAIP | 4671 |
| NAP1L3 | 4675 |
| NAPSB | 256236 |
| NBEA | 26960 |
| NBEAL1 | 65065 |
| NCCRP1 | 342897 |
| NCF1 | 653361 |
| NCF1B | 654816 |
| NCF1C | 654817 |
| NCF2 | 4688 |
| NCF4 | 4689 |
| NCKAP1L | 3071 |
| NCOA2 | 10499 |
| NCR1 | 9437 |
| NCR3 | 259197 |
| NEGR1 | 257194 |
| NFAM1 | 150372 |
| NFATC2 | 4773 |
| NFKB2 | 4791 |
| NFKBID | 84807 |
| NFKBIE | 4794 |
| NFYA | 4800 |
| NFYB | 4801 |
| NFYC | 4802 |
| NHLRC2 | 374354 |
| NHSL2 | 340527 |
| NID2 | 22795 |
| NIPAL4 | 348938 |

| | |
|---|---|
| NKG7 | 4818 |
| NLRC3 | 197358 |
| NLRC4 | 58484 |
| NLRC5 | 84166 |
| NLRP12 | 91662 |
| NLRP3 | 114548 |
| NMI | 9111 |
| NOD2 | 64127 |
| NOTCH4 | 4855 |
| NOVA2 | 4858 |
| NOX4 | 50507 |
| NR1H3 | 10062 |
| NR5A2 | 2494 |
| NRP1 | 8829 |
| NRXN3 | 9369 |
| NT5E | 4907 |
| NTM | 50863 |
| NTNG2 | 84628 |
| NTRK1 | 4914 |
| OAS1 | 4938 |
| OAS2 | 4939 |
| OAS3 | 4940 |
| OASL | 8638 |
| TENM3 | 55714 |
| OGFRL1 | 79627 |
| OGN | 4969 |
| OLFML1 | 283298 |
| OLFML2B | 25903 |
| OLFML3 | 56944 |
| OLR1 | 4973 |
| OMD | 4958 |
| OSCAR | 126014 |
| OSM | 5008 |
| OSMR | 9180 |
| OTOA | 146183 |
| OVOL1 | 5017 |
| P2RX1 | 5023 |
| P2RX4 | 5025 |
| P2RX5 | 5026 |
| P2RX7 | 5027 |
| P2RY10 | 27334 |
| P2RY11 | 5032 |
| P2RY12 | 64805 |
| P2RY13 | 53829 |
| P2RY14 | 9934 |
| P2RY8 | 286530 |
| P4HA3 | 283208 |
| PABPC5 | 140886 |
| PADI2 | 11240 |
| PAFAH1B2 | 5049 |
| PAG1 | 55824 |
| PAK5 | 57144 |

| | |
|---|---|
| PALM2-AKAP2 | 445815 |
| PARM1 | 25849 |
| PARP12 | 64761 |
| PARP14 | 54625 |
| PARP15 | 165631 |
| PARP9 | 83666 |
| PARVG | 64098 |
| PATL2 | 197135 |
| PATZ1 | 23598 |
| PBX4 | 80714 |
| PCDH12 | 51294 |
| PCDH17 | 27253 |
| PCDH18 | 54510 |
| PCDHGA12 | 26025 |
| PCOLCE | 5118 |
| PCYOX1L | 78991 |
| PDCD1 | 5133 |
| PDCD1LG2 | 80380 |
| PDE1A | 5136 |
| PDE1B | 5153 |
| PDE3A | 5139 |
| PDE3B | 5140 |
| PDE4B | 5142 |
| PDE6G | 5148 |
| PDGFA | 5154 |
| PDGFB | 5155 |
| PDGFC | 56034 |
| PDGFRA | 5156 |
| PDGFRB | 5159 |
| PDGFRL | 5157 |
| PDIA2 | 64714 |
| PDIA3 | 2923 |
| PDZK1IP1 | 10158 |
| PDZRN3 | 23024 |
| PECAM1 | 5175 |
| PEG3 | 5178 |
| PFDN2 | 5202 |
| PGLYRP4 | 57115 |
| PGM5 | 5239 |
| PHACTR1 | 221692 |
| PIK3AP1 | 118788 |
| PIK3CG | 5294 |
| PIK3R5 | 23533 |
| PIK3R6 | 146850 |
| PILRA | 29992 |
| PIM2 | 11040 |
| PIP4K2A | 5305 |
| PKD2L1 | 9033 |
| PKHD1L1 | 93035 |
| PKIB | 5570 |
| PKP3 | 11187 |
| PLA1A | 51365 |

| | |
|---|---|
| PLA2G2D | 26279 |
| PLA2G4E | 123745 |
| PLA2G7 | 7941 |
| PLAC9 | 219348 |
| PLCB2 | 5330 |
| PLCB4 | 5332 |
| PLCL1 | 5334 |
| PLCL2 | 23228 |
| PLD4 | 122618 |
| PLEK2 | 26499 |
| PLEK | 5341 |
| PLEKHM3 | 389072 |
| PLEKHN1 | 84069 |
| PLEKHO1 | 51177 |
| PLEKHO2 | 80301 |
| PLIN3 | 10226 |
| PLVAP | 83483 |
| PLXDC1 | 57125 |
| PLXNA4 | 91584 |
| PLXNC1 | 10154 |
| PLXND1 | 23129 |
| PML | 5371 |
| PMP22 | 5376 |
| PNLIPRP3 | 119548 |
| PNMA2 | 10687 |
| PNOC | 5368 |
| PODN | 127435 |
| POSTN | 10631 |
| POU2AF1 | 5450 |
| POU2F2 | 5452 |
| PLPP3 | 8613 |
| PLPP4 | 196051 |
| PLPP7 | 84814 |
| PPBP | 5473 |
| PPFIA2 | 8499 |
| PPL | 5493 |
| PPM1H | 57460 |
| PPM1M | 132160 |
| PPP1R13L | 10848 |
| PPP1R16B | 26051 |
| PPP1R9A | 55607 |
| PRAM1 | 84106 |
| PRELP | 5549 |
| PREX1 | 57580 |
| PREX2 | 80243 |
| PRF1 | 5551 |
| PRG2 | 5553 |
| PRKAR2A | 5576 |
| PRKAR2B | 5577 |
| PRKCB | 5579 |
| PRKCQ | 5588 |
| PRKG1 | 5592 |

| | |
|---|---|
| PRL | 5617 |
| PRLR | 5618 |
| PROCR | 10544 |
| PROM1 | 8842 |
| PRRX1 | 5396 |
| PRSS27 | 83886 |
| PRTG | 283659 |
| PSAP | 5660 |
| PSMB10 | 5699 |
| PSMB8 | 5696 |
| PSMB9 | 5698 |
| PSMC1 | 5700 |
| PSMC2 | 5701 |
| PSMC3 | 5702 |
| PSMC4 | 5704 |
| PSMC5 | 5705 |
| PSMC6 | 5706 |
| PSMD10 | 5716 |
| PSMD11 | 5717 |
| PSMD13 | 5719 |
| PSMD14 | 10213 |
| PSMD1 | 5707 |
| PSMD2 | 5708 |
| PSMD3 | 5709 |
| PSMD4 | 5710 |
| PSMD5 | 5711 |
| PSMD6 | 9861 |
| PSMD7 | 5713 |
| PSMD8 | 5714 |
| PSME1 | 5720 |
| PSME2 | 5721 |
| PSME3 | 10197 |
| PSTPIP1 | 9051 |
| PTAFR | 5724 |
| PTCRA | 171558 |
| PTGDR | 5729 |
| PTGDS | 5730 |
| PTGER2 | 5732 |
| PTGFR | 5737 |
| PTGIR | 5739 |
| PTGIS | 5740 |
| PTH1R | 5745 |
| PTPN22 | 26191 |
| PTPN6 | 5777 |
| PTPN7 | 5778 |
| PTPRB | 5787 |
| PTPRC | 5788 |
| PTPRCAP | 5790 |
| PTPRJ | 5795 |
| PTPRM | 5797 |
| PTPRO | 5800 |
| CAVIN1 | 284119 |

| | |
|---|---|
| PUS10 | 150962 |
| PVR | 5817 |
| PVRIG | 79037 |
| NECTIN2 | 5819 |
| NECTIN3 | 25945 |
| PYHIN1 | 149628 |
| QPRT | 23475 |
| RAB20 | 55647 |
| RAB33A | 9363 |
| RAB37 | 326624 |
| RAB39A | 54734 |
| RAB39B | 116442 |
| RAB42 | 115273 |
| RAB8B | 51762 |
| RAD23B | 5887 |
| RAD54L2 | 23132 |
| RAET1E | 135250 |
| RAET1G | 353091 |
| RAET1L | 154064 |
| RAI2 | 10742 |
| RAMP3 | 10268 |
| RAPGEF2 | 9693 |
| RAPGEF6 | 51735 |
| RARRES2 | 5919 |
| RARRES3 | 5920 |
| RASAL3 | 64926 |
| RASGRF2 | 5924 |
| RASGRP2 | 10235 |
| RASGRP3 | 25780 |
| RASGRP4 | 115727 |
| RASL12 | 51285 |
| RASSF2 | 9770 |
| RASSF3 | 283349 |
| RASSF4 | 83937 |
| RASSF5 | 83593 |
| RASSF6 | 166824 |
| RBM38 | 55544 |
| RBP5 | 83758 |
| RC3H2 | 54542 |
| RCAN2 | 10231 |
| RCN3 | 57333 |
| RCSD1 | 92241 |
| RDH12 | 145226 |
| RECK | 8434 |
| REL | 5966 |
| RELB | 5971 |
| RELN | 5649 |
| RENBP | 5973 |
| REST | 5978 |
| RFTN1 | 23180 |
| RFX5 | 5993 |
| RFXANK | 8625 |

| | |
|---|---|
| RFXAP | 5994 |
| RGL1 | 23179 |
| RGL4 | 266747 |
| RGPD1 | 400966 |
| RGS13 | 6003 |
| RGS1 | 5996 |
| RGS18 | 64407 |
| RGS5 | 8490 |
| RHOD | 29984 |
| RHOH | 399 |
| RHOJ | 57381 |
| RIF1 | 55183 |
| RIMKLA | 284716 |
| RIN1 | 9610 |
| RIN3 | 79890 |
| CARMIL2 | 146206 |
| RNASE1 | 6035 |
| RNASE2 | 6036 |
| RNASE6 | 6039 |
| RNASE7 | 84659 |
| RNF125 | 54941 |
| RNF166 | 115992 |
| RNF180 | 285671 |
| RNF222 | 643904 |
| LAMTOR2 | 28956 |
| ROBO4 | 54538 |
| ROCK2 | 9475 |
| ROR1 | 4919 |
| RPS6KA4 | 8986 |
| RRN3P2 | 653390 |
| RSAD2 | 91543 |
| RTKN2 | 219790 |
| RTN1 | 6252 |
| RTP4 | 64108 |
| RUFY4 | 285180 |
| NA | 84127 |
| RUNX1T1 | 862 |
| RUNX3 | 864 |
| S100A12 | 6283 |
| S100A16 | 140576 |
| S100A2 | 6273 |
| S100A7 | 6278 |
| S100A7A | 338324 |
| S100A8 | 6279 |
| S100A9 | 6280 |
| S100B | 6285 |
| S1PR1 | 1901 |
| S1PR4 | 8698 |
| SALL2 | 6297 |
| SAMD14 | 201191 |
| SAMD3 | 154075 |
| SAMD9 | 54809 |

| | | |
|---|---|---|
| SAMD9L | | 219285 |
| SAMHD1 | | 25939 |
| SAMSN1 | | 64092 |
| SARDH | | 1757 |
| SASH3 | | 54440 |
| SBNO1 | | 55206 |
| SBSN | | 374897 |
| SCARF1 | | 8578 |
| SCARF2 | | 91179 |
| SCEL | | 8796 |
| SCML4 | | 256380 |
| SCN7A | | 6332 |
| SCUBE3 | | 222663 |
| SDC2 | | 6383 |
| SDCBP2 | | 27111 |
| SDR9C7 | | 121214 |
| SDS | | 10993 |
| SDSL | | 113675 |
| SEC24A | | 10802 |
| SEC24D | | 9871 |
| SECISBP2L | | 9728 |
| SECTM1 | | 6398 |
| SELENBP1 | | 8991 |
| SELL | | 6402 |
| SELP | | 6403 |
| SELPLG | | 6404 |
| | 43344 | 1731 |
| | 43349 | 23157 |
| SERINC5 | | 256987 |
| SERPINA1 | | 5265 |
| SERPINE1 | | 5054 |
| SERPINF1 | | 5176 |
| SERPING1 | | 710 |
| SFMBT2 | | 57713 |
| SFN | | 2810 |
| SFRP2 | | 6423 |
| SFTPB | | 6439 |
| SGCD | | 6444 |
| SGIP1 | | 84251 |
| POMK | | 84197 |
| PEAK1 | | 79834 |
| SH2B3 | | 10019 |
| SH2D1A | | 4068 |
| SH2D2A | | 9047 |
| SH2D3C | | 10044 |
| SH2D5 | | 400745 |
| SHE | | 126669 |
| SEM1 | | 7979 |
| SIGLEC10 | | 89790 |
| SIGLEC11 | | 114132 |
| SIGLEC12 | | 89858 |
| SIGLEC14 | | 100049587 |

| | |
|---|---|
| SIGLEC1 | 6614 |
| SIGLEC5 | 8778 |
| SIGLEC6 | 946 |
| SIGLEC7 | 27036 |
| SIGLEC8 | 27181 |
| SIGLEC9 | 27180 |
| SIGLEC17P | 284367 |
| SIRPB1 | 10326 |
| SIRPB2 | 284759 |
| SIRPG | 55423 |
| SIT1 | 27240 |
| SKAP1 | 8631 |
| SLA2 | 84174 |
| SLA | 6503 |
| SLAMF1 | 6504 |
| SLAMF6 | 114836 |
| SLAMF7 | 57823 |
| SLAMF8 | 56833 |
| SLC10A2 | 6555 |
| SLC11A1 | 6556 |
| SLC12A3 | 6559 |
| SLC15A3 | 51296 |
| SLC17A9 | 63910 |
| SLC18A2 | 6571 |
| SLC1A7 | 6512 |
| SLC24A4 | 123041 |
| SLC25A45 | 283130 |
| SLC29A3 | 55315 |
| SLC2A5 | 6518 |
| SLC34A2 | 10568 |
| SLC39A2 | 29986 |
| SLC45A3 | 85414 |
| SLC6A12 | 6539 |
| SLC7A7 | 9056 |
| SLC8A1 | 6546 |
| SLCO2B1 | 11309 |
| SLCO5A1 | 81796 |
| NA | 342615 |
| SLIT2 | 9353 |
| SLIT3 | 6586 |
| SLURP1 | 57152 |
| SMAP2 | 64744 |
| SMPDL3B | 27293 |
| SNAI3 | 333929 |
| SNED1 | 25992 |
| SNRPF | 6636 |
| SNTB1 | 6641 |
| SNX20 | 124460 |
| SOD3 | 6649 |
| SON | 6651 |
| SOX17 | 64321 |
| SOX5 | 6660 |

| | |
|---|---|
| SP100 | 6672 |
| SP110 | 3431 |
| SP140 | 11262 |
| SPAG4 | 6676 |
| SPARC | 6678 |
| SPARCL1 | 8404 |
| SPATA13 | 221178 |
| SPI1 | 6688 |
| SPIB | 6689 |
| SPN | 6693 |
| SPNS3 | 201305 |
| SPOCK2 | 9806 |
| SPON1 | 10418 |
| SPRR1A | 6698 |
| SPRR1B | 6699 |
| SPRR2D | 6703 |
| SPRR2E | 6704 |
| SPRR2G | 6706 |
| SPRY1 | 10252 |
| SRGN | 5552 |
| SSC5D | 284297 |
| SSTR3 | 6753 |
| ST3GAL2 | 6483 |
| ST3GAL5 | 8869 |
| ST3GAL6 | 10402 |
| ST6GAL1 | 6480 |
| ST6GALNAC3 | 256435 |
| ST8SIA4 | 7903 |
| STAB1 | 23166 |
| STAC3 | 246329 |
| STAP1 | 26228 |
| STARD13 | 90627 |
| STARD8 | 9754 |
| STAT1 | 6772 |
| STAT4 | 6775 |
| STAT5A | 6776 |
| STK17B | 9262 |
| STK33 | 65975 |
| STRN | 6801 |
| STXBP6 | 29091 |
| SUCNR1 | 56670 |
| SULF1 | 23213 |
| SULT1C2 | 6819 |
| SULT1C4 | 27233 |
| SULT2B1 | 6820 |
| SUSD3 | 203328 |
| SVOPL | 136306 |
| SYNE1 | 23345 |
| SYT11 | 23208 |
| SYTL3 | 94120 |
| TAGAP | 117289 |
| TAOK1 | 57551 |

| | |
|---|---|
| TAP1 | 6890 |
| TAP2 | 6891 |
| TAPBP | 6892 |
| TAPBPL | 55080 |
| TARP | 445347 |
| TBC1D10C | 374403 |
| TBCEL | 219899 |
| TBX21 | 30009 |
| TBXA2R | 6915 |
| TBXAS1 | 6916 |
| TCEAL7 | 56849 |
| TCIRG1 | 10312 |
| TCL1A | 8115 |
| TCN2 | 6948 |
| TEK | 7010 |
| TESC | 54997 |
| TFEC | 22797 |
| TGFB1 | 7040 |
| TGFB2 | 7042 |
| TGFB3 | 7043 |
| TGFBI | 7045 |
| TGFBR1 | 7046 |
| TGFBR2 | 7048 |
| TGFBRAP1 | 9392 |
| TGM1 | 7051 |
| TGM2 | 7052 |
| THBS1 | 7057 |
| THBS2 | 7058 |
| THEMIS | 387357 |
| THPO | 7066 |
| THSD7A | 221981 |
| THY1 | 7070 |
| TIE1 | 7075 |
| TIFAB | 497189 |
| TIGIT | 201633 |
| TIMD4 | 91937 |
| PAM16 | 51025 |
| TIMM50 | 92609 |
| TIMP2 | 7077 |
| TIMP3 | 7078 |
| TLR10 | 81793 |
| TLR1 | 7096 |
| TLR4 | 7099 |
| TLR5 | 7100 |
| TLR7 | 51284 |
| TLR8 | 51311 |
| TLR9 | 54106 |
| TM4SF18 | 116441 |
| TM6SF1 | 53346 |
| DCSTAMP | 81501 |
| TMC8 | 147138 |
| TMEM106A | 113277 |

| | |
|---|---|
| TMEM119 | 338773 |
| TMEM140 | 55281 |
| IGFLR1 | 79713 |
| TMEM150B | 284417 |
| TMEM156 | 80008 |
| TMEM170B | 100113407 |
| TMEM176A | 55365 |
| TMEM176B | 28959 |
| TMEM200A | 114801 |
| TMEM204 | 79652 |
| TMEM229B | 161145 |
| TMEM233 | 387890 |
| TMEM26 | 219623 |
| TMEM47 | 83604 |
| TMEM79 | 84283 |
| SYNDIG1 | 79953 |
| TMIGD2 | 126259 |
| TNF | 7124 |
| TNFAIP6 | 7130 |
| TNFAIP8L2 | 79626 |
| TNFRSF10A | 8797 |
| TNFRSF10B | 8795 |
| TNFRSF10C | 8794 |
| TNFRSF10D | 8793 |
| TNFRSF11A | 8792 |
| TNFRSF11B | 4982 |
| TNFRSF12A | 51330 |
| TNFRSF13B | 23495 |
| TNFRSF13C | 115650 |
| TNFRSF14 | 8764 |
| TNFRSF17 | 608 |
| TNFRSF18 | 8784 |
| TNFRSF1A | 7132 |
| TNFRSF1B | 7133 |
| TNFRSF25 | 8718 |
| TNFRSF4 | 7293 |
| TNFRSF6B | 8771 |
| TNFRSF8 | 943 |
| TNFRSF9 | 3604 |
| TNFSF10 | 8743 |
| TNFSF11 | 8600 |
| TNFSF12 | 8742 |
| TNFSF12-TNFSF13 | 407977 |
| TNFSF13 | 8741 |
| TNFSF13B | 10673 |
| TNFSF14 | 8740 |
| TNFSF15 | 9966 |
| TNFSF18 | 8995 |
| TNFSF4 | 7292 |
| TNFSF8 | 944 |
| TNFSF9 | 8744 |
| TNIK | 23043 |

| | |
|---|---|
| TNIP3 | 79931 |
| TNN | 63923 |
| TNNT2 | 7139 |
| TNS3 | 64759 |
| TOX | 9760 |
| TPK1 | 27010 |
| TPSAB1 | 7177 |
| TPSB2 | 64499 |
| TPSD1 | 23430 |
| TPSG1 | 25823 |
| TRAF1 | 7185 |
| TRAF3IP3 | 80342 |
| TRANK1 | 9881 |
| TRAT1 | 50852 |
| TREM1 | 54210 |
| TREM2 | 54209 |
| TREML1 | 340205 |
| TRIM21 | 6737 |
| TRIM22 | 10346 |
| TRIM61 | 391712 |
| TRPC4AP | 26133 |
| TRPV2 | 51393 |
| TRPV3 | 162514 |
| TSHR | 7253 |
| TSHZ3 | 57616 |
| TSLP | 85480 |
| TSPAN11 | 441631 |
| TSPAN32 | 10077 |
| TSPAN4 | 7106 |
| TTBK2 | 146057 |
| TTC16 | 158248 |
| TTC21B | 79809 |
| TTC24 | 164118 |
| TTC37 | 9652 |
| TUBA4A | 7277 |
| TUBB6 | 84617 |
| TXK | 7294 |
| NME8 | 51314 |
| TYMP | 1890 |
| TYROBP | 7305 |
| UBA7 | 7318 |
| UBASH3A | 53347 |
| UBD | 10537 |
| UBE2L6 | 9246 |
| UBR1 | 197131 |
| UBXN11 | 91544 |
| UBXN1 | 51035 |
| UCP2 | 7351 |
| UHMK1 | 127933 |
| ULBP1 | 80329 |
| ULBP2 | 80328 |
| ULBP3 | 79465 |

| | |
|---|---|
| UNC13D | 201294 |
| UNC5C | 8633 |
| UNC93B1 | 81622 |
| USHBP1 | 83878 |
| USP12 | 219333 |
| USP51 | 158880 |
| UTS2 | 10911 |
| VAMP5 | 10791 |
| VASH1 | 22846 |
| VAV1 | 7409 |
| VCAM1 | 7412 |
| VCAN | 1462 |
| VEGFA | 7422 |
| VEGFB | 7423 |
| VEGFC | 7424 |
| VENTX | 27287 |
| VGLL3 | 389136 |
| VIM | 7431 |
| VMO1 | 284013 |
| VNN2 | 8875 |
| VPREB3 | 29802 |
| VPS37D | 155382 |
| VSIG4 | 11326 |
| VWF | 7450 |
| WARS | 7453 |
| WAS | 7454 |
| WDFY4 | 57705 |
| WFDC12 | 128488 |
| WIPF1 | 7456 |
| WISP1 | 8840 |
| WNT2 | 7472 |
| XAF1 | 54739 |
| XBP1 | 7494 |
| XCL1 | 6375 |
| XCL2 | 6846 |
| XCR1 | 2829 |
| XKR8 | 55113 |
| XPNPEP2 | 7512 |
| ZAP70 | 7535 |
| ZBP1 | 81030 |
| ZBTB10 | 65986 |
| ZBTB32 | 27033 |
| ZC3H12D | 340152 |
| ZCCHC24 | 219654 |
| ZDHHC20 | 253832 |
| ZEB1 | 6935 |
| ZEB2 | 9839 |
| ZFPM2 | 23414 |
| ZKSCAN1 | 7586 |
| ZMYND15 | 84225 |
| ZNF185 | 7739 |
| ZKSCAN8 | 7745 |

| | |
|---|---|
| ZNF215 | 7762 |
| ZNF366 | 167465 |
| ZNF423 | 23090 |
| ZNF469 | 84627 |
| ZNF521 | 25925 |
| ZNF620 | 253639 |
| ZNF660 | 285349 |
| ZNF671 | 79891 |
| ZNF683 | 257101 |
| ZNF804A | 91752 |
| ZNF80 | 7634 |
| ZNF827 | 152485 |
| ZNF831 | 128611 |
| ZNF835 | 90485 |
