# Supplementary material for: Analysis of Immune Gene Expression Subtypes Reveals Osteosarcoma Immune Heterogeneity
Source: J Oncol. 2021 Mar 1;2021:6649412. doi: 10.1155/2021/6649412 (PMC7939746; doi:10.1155/2021/6649412)
Supplement: Supplementary Materials — Supplementary Figure S1. Consensus matrix for immune-related gene modules derived from consensus clustering analysis. Supplementary Figure S2. Expression feature of immune subtypes in training TARGET cohort. Supplementary Figure S3. The validation of immune subtypes in the independent GEO cohort. Supplementary Figure S4. The intracluster heterogeneity revealed by the immune landscape analysis and different subcluster molecular characteristics of patients in immune subtypes 1 and 4 are shown. Supplementary Table S1. The bulk RNA-seq data retrieved from the OS-project. Supplementary Table S2. The collected 1989 immune-related genes. Supplementary Table S3. The immune subtype annotation at the patient level in the TARGET cohort. Supplementary Table S4. The immune-related gene modules feature. Supplementary Table S5. Functional enrichment analysis of gene modules. Supplementary Table S6. The immune-related molecular features of the TARGET patients. Supplementary Table S7. Correlation of average gene module scores between molecular characteristics and immune subtypes. [file 6649412.f1.zip › 6649412.f1/Supplementary Table S4.pdf]

k	cluster	item	itemConsensus
7	1	2	0.74836571
7	2	23460	0.947034253
7	2	10351	0.945216922
7	2	10350	0.950201497
7	3	5243	0.765990848
7	2	10060	0.885234256
7	4	225	0.572815815
7	1	51225	0.794470985
7	2	25890	0.887077794
7	3	1636	0.829496732
7	6	43	0.783319033
7	2	55289	0.946213577
7	1	54	0.797178931
7	3	51703	0.746645229
7	4	59	0.894632621
7	4	87	0.895241794
7	6	90	0.760048449
7	6	91	0.896819734
7	2	92	0.756714978
7	2	93	0.943655231
7	3	94	0.834054961
7	6	8038	0.775645494
7	1	10863	0.446196523
7	1	101	0.470982585
7	1	27299	0.802917739
7	1	81794	0.727421561
7	4	81792	0.842831333
7	4	140766	0.893540045
7	2	170690	0.717641689
7	3	9509	0.794911885
7	4	9507	0.891458734
7	6	11096	0.755421139
7	3	56999	0.818322781
7	3	9719	0.825909955
7	5	23536	0.399682907
7	3	196883	0.820624354
7	2	116	0.944679881
7	3	135	0.63422576
7	7	141	0.567392328
7	4	150	0.881571748
7	5	11047	0.536460105
7	4	165	0.894636297
7	2	3899	0.945746515
7	4	185	0.875285051
7	5	199	0.598327803
7	4	9447	0.889591636
7	6	9590	0.871779023
7	6	11217	0.882257208
7	2	9495	0.725421349
7	5	80709	0.576225569
7	7	216	0.397067927

7	7	221	0.566720997
7	5	240	0.592606766
7	5	241	0.579581047
7	2	59344	0.947053934
7	2	268	0.467972499
7	2	269	0.946365733
7	3	386724	0.655953574
7	7	270	0.526401875
7	4	273	0.881749053
7	4	9068	0.690326363
7	4	23452	0.888286936
7	6	287	0.889510439
7	6	26057	0.899156071
7	5	118932	0.541788094
7	6	91526	0.853541554
7	4	84168	0.863726547
7	1	118429	0.326379931
7	1	309	0.775535129
7	5	313	0.581258299
7	3	8639	0.814956014
7	6	8905	0.79338174
7	1	8546	0.412815022
7	1	54518	0.54529274
7	6	323	0.857187009
7	7	200315	0.595790288
7	2	140564	0.888456306
7	7	60489	0.664588789
7	5	341	0.591684765
7	1	344	0.548122746
7	5	348	0.588102775
7	7	80833	0.684764568
7	7	80830	0.704250627
7	2	89872	0.941573357
7	3	358	0.556136293
7	7	366	0.719220902
7	5	374	0.374841217
7	2	55843	0.929069601
7	6	93663	0.558349505
7	2	58504	0.901187547
7	5	9938	0.498361584
7	5	257106	0.603607429
7	1	393	0.575200232
7	6	395	0.752893955
7	1	64333	0.584734835
7	5	397	0.565157857
7	3	22899	0.831986248
7	3	9459	0.701174175
7	7	10865	0.673047071
7	6	10550	0.50513087
7	1	408	0.640180453
7	5	409	0.578841814
7	1	411	0.679865661

7	2	420	0.947773068
7	7	427	0.596566953
7	1	433	0.795031233
7	4	54829	0.892402923
7	2	80150	0.897550221
7	6	55252	0.896609512
7	6	11101	0.884151495
7	4	57194	0.893737144
7	2	489	0.948320716
7	2	10396	0.904275898
7	3	79895	0.589254415
7	3	552	0.549609226
7	2	563	0.945306452
7	7	567	0.430999706
7	2	27087	0.943841212
7	6	55024	0.523511319
7	5	10538	0.517646573
7	7	116071	0.72554498
7	5	597	0.53110301
7	2	79370	0.941973838
7	3	255877	0.834499259
7	4	624	0.878523302
7	2	8419	0.943481341
7	3	633	0.609585327
7	6	80114	0.891475327
7	5	51411	0.575887402
7	6	57448	0.895893473
7	2	640	0.946090799
7	2	650	0.555868601
7	6	55589	0.778625106
7	1	655	0.691524159
7	6	657	0.883373138
7	2	658	0.946766924
7	6	659	0.871035411
7	4	54796	0.888330801
7	7	683	0.548101186
7	5	684	0.565293313
7	1	695	0.661048181
7	5	151888	0.382695478
7	6	10385	0.882814003
7	7	11119	0.714714136
7	7	11118	0.656197405
7	7	10384	0.700377702
7	1	170371	0.799036385
7	5	9556	0.327787152
7	5	84419	0.39064255
7	2	283897	0.946768786
7	2	199675	0.403870422
7	2	79098	0.73082311
7	2	148345	0.94932927
7	5	128346	0.588564246
7	3	79630	0.627322243

7	5	712	0.576912767
7	5	713	0.585660472
7	5	714	0.592566505
7	4	114905	0.894260945
7	5	717	0.557584556
7	5	718	0.58150903
7	5	719	0.586635545
7	5	728	0.556360792
7	2	730	0.942449248
7	4	775	0.888203699
7	2	9254	0.893950761
7	1	93589	0.797749325
7	2	794	0.774444674
7	4	800	0.874034305
7	7	811	0.5215026
7	1	8536	0.290373839
7	2	814	0.948105576
7	6	821	0.890653246
7	5	84433	0.574747024
7	6	22900	0.812676493
7	5	64170	0.582750055
7	1	838	0.732685061
7	4	857	0.893976973
7	2	26112	0.943595593
7	5	6357	0.532952842
7	3	6358	0.858848238
7	2	6359	0.946289374
7	2	6360	0.949986971
7	1	6361	0.76186367
7	5	6362	0.549376005
7	5	6363	0.548149681
7	2	6364	0.943172835
7	5	6366	0.539870012
7	2	6367	0.946307254
7	5	6368	0.535562475
7	3	6369	0.733353658
7	2	6370	0.947662091
7	5	10344	0.268787075
7	5	6347	0.591083228
7	6	56477	0.905582855
7	5	6348	0.595086832
7	5	414062	0.557323899
7	5	6351	0.586172463
7	5	6352	0.524758293
7	7	6354	0.731638998
7	5	6355	0.430980356
7	6	904	0.888128688
7	5	2826	0.318980575
7	1	1230	0.798507951
7	5	729230	0.526434862
7	2	1232	0.947612328
7	2	1233	0.946539688

7	5	1234	0.57845527
7	2	1235	0.917605456
7	5	1236	0.541497912
7	2	1237	0.708800584
7	2	10803	0.791081379
7	1	9034	0.758744304
7	5	929	0.595688884
7	2	11126	0.953386579
7	5	9332	0.588239669
7	5	4064	0.570523467
7	2	930	0.374149484
7	1	909	0.768318074
7	2	910	0.903777353
7	5	911	0.527350416
7	5	912	0.364902838
7	2	913	0.948357875
7	3	4345	0.834930589
7	5	131450	0.567966297
7	6	50489	0.827095653
7	5	30835	0.568960813
7	2	10666	0.947451867
7	2	933	0.925721722
7	5	51744	0.46578659
7	4	57124	0.40135233
7	7	29126	0.560242874
7	5	940	0.584079096
7	5	914	0.550008868
7	5	11314	0.591329762
7	1	10871	0.794970787
7	5	342510	0.451284756
7	1	124599	0.79271322
7	5	146722	0.563808262
7	3	9936	0.570993116
7	1	945	0.794699197
7	3	947	0.835247231
7	6	948	0.447851891
7	5	951	0.578710254
7	5	915	0.555391513
7	5	916	0.542824685
7	2	917	0.935129509
7	1	958	0.795192594
7	5	959	0.430544042
7	5	962	0.514646954
7	1	920	0.799499094
7	5	1043	0.559781159
7	1	963	0.495285451
7	7	965	0.472882285
7	5	921	0.531193392
7	1	968	0.803490847
7	2	923	0.453474907
7	5	969	0.53513587
7	1	971	0.697965233

7	5	972	0.588457518
7	5	924	0.569943033
7	5	973	0.523505342
7	3	974	0.529220843
7	5	941	0.565380424
7	1	8832	0.795561488
7	5	942	0.578669647
7	5	925	0.526419635
7	2	10225	0.370142376
7	6	56990	0.896430243
7	6	1009	0.794414322
7	2	28316	0.945485309
7	2	1001	0.618241543
7	3	1003	0.858423574
7	2	1004	0.946383613
7	2	6792	0.950866964
7	2	56971	0.733205513
7	5	90273	0.572996886
7	5	1089	0.596292481
7	5	375298	0.542211278
7	3	1071	0.815106238
7	4	79094	0.326353172
7	2	1118	0.943852134
7	7	1123	0.706228729
7	6	8646	0.807038068
7	6	91851	0.885098022
7	2	8973	0.946718865
7	1	166012	0.794726252
7	3	9435	0.824879833
7	5	4261	0.536050956
7	4	8483	0.892589698
7	5	1154	0.581387372
7	4	23529	0.838148931
7	5	10462	0.585432744
7	1	6320	0.796573448
7	2	160364	0.334839672
7	3	161198	0.831441352
7	2	51267	0.947143816
7	3	7123	0.619946406
7	1	50856	0.800946381
7	2	338339	0.914423709
7	2	26253	0.859091763
7	5	23601	0.590577948
7	2	283420	0.390651932
7	3	1193	0.311661934
7	7	9022	0.604924711
7	1	53405	0.616946845
7	6	9575	0.905663735
7	5	1212	0.394545494
7	7	1215	0.620264942
7	5	1240	0.488121259
7	2	84518	0.364115944

7	2	1269	0.94600448
7	6	1270	0.892141412
7	6	1271	0.889046157
7	4	1300	0.885801543
7	4	1301	0.890312825
7	4	1303	0.88615689
7	4	7373	0.895684478
7	3	1306	0.548338178
7	3	80781	0.822109467
7	4	1277	0.822761512
7	4	1278	0.791666999
7	4	1281	0.894412334
7	3	1282	0.824764817
7	3	1284	0.832462264
7	4	1289	0.891716657
7	6	1290	0.831192489
7	3	50509	0.818854567
7	4	1291	0.896293441
7	4	1292	0.892986442
7	4	1293	0.893436222
7	4	1295	0.890615293
7	1	81035	0.336439731
7	2	10699	0.946418824
7	5	11151	0.612101657
7	5	23406	0.578432805
7	4	1359	0.871777679
7	2	57699	0.923082717
7	5	54504	0.60250803
7	7	8532	0.571719401
7	2	1378	0.948128631
7	2	1380	0.948810756
7	6	1385	0.892985602
7	1	90993	0.6907029
7	6	1389	0.50664386
7	4	83716	0.891013689
7	2	64109	0.952090177
7	5	56253	0.27266881
7	5	1414	0.568164992
7	1	1435	0.686713414
7	1	1436	0.794399545
7	2	1438	0.944188745
7	1	1439	0.63546689
7	2	1440	0.912919744
7	5	1441	0.547861756
7	2	114784	0.609253792
7	4	8530	0.583505322
7	4	1489	0.703191702
7	4	1490	0.528615985
7	7	115908	0.402048523
7	5	1493	0.536232933
7	1	1508	0.471880168
7	2	1510	0.948798006

7	3	1511	0.445555766
7	1	1513	0.754670889
7	7	1514	0.69625549
7	6	1519	0.774996236
7	5	1520	0.572006118
7	5	1521	0.445683074
7	1	1522	0.79769677
7	2	83992	0.950490122
7	5	6376	0.332148898
7	5	1524	0.527530634
7	5	3627	0.484371858
7	5	6373	0.522165122
7	4	6387	0.311234746
7	5	2919	0.532752246
7	5	10563	0.476483976
7	5	58191	0.592095066
7	5	2920	0.5482865
7	5	2921	0.560163908
7	1	6374	0.738446651
7	5	6372	0.292715055
7	5	4283	0.524279998
7	5	2833	0.548485914
7	3	7852	0.71680577
7	5	10663	0.533400596
7	5	80231	0.594571778
7	3	79742	0.838530201
7	1	1535	0.489801649
7	5	1536	0.593071924
7	2	26999	0.94767441
7	6	1545	0.736597925
7	4	1593	0.890632621
7	2	10800	0.9430175
7	2	57105	0.86420194
7	6	23500	0.877697958
7	4	1601	0.813012726
7	4	51339	0.707206285
7	2	1621	0.907755697
7	6	285761	0.888835241
7	6	8642	0.5333816
7	4	1634	0.891611324
7	6	84301	0.893278428
7	6	4921	0.791385529
7	7	23586	0.691705027
7	5	79958	0.532359779
7	2	27147	0.714761153
7	2	91319	0.882758783
7	1	115817	0.386581275
7	1	10170	0.785202476
7	6	85458	0.906119944
7	4	27123	0.635653591
7	6	10395	0.842787465
7	3	54567	0.828632176

7	6	23312	0.889397607
7	2	1769	0.944862874
7	2	85479	0.943313115
7	6	55619	0.654301883
7	1	139818	0.547440304
7	5	1794	0.589894853
7	6	9732	0.796694541
7	5	81704	0.49424527
7	7	1796	0.621774266
7	5	9046	0.59630907
7	1	79930	0.485818876
7	6	55816	0.567341777
7	2	220164	0.782427782
7	3	1800	0.476909208
7	5	64174	0.570801288
7	6	54878	0.884169264
7	4	1805	0.816443211
7	2	1823	0.945651832
7	2	1828	0.944589761
7	6	1832	0.839475548
7	6	80824	0.89348795
7	1	1846	0.795553802
7	3	8291	0.821333872
7	6	1875	0.838674665
7	2	64641	0.942397956
7	5	10148	0.594912234
7	4	1842	0.894064543
7	2	1896	0.945766993
7	2	60401	0.944913719
7	2	10913	0.944651808
7	4	1909	0.643733084
7	3	1910	0.669349723
7	3	30008	0.455835414
7	4	9343	0.705408995
7	2	1950	0.953133306
7	4	1956	0.892532705
7	4	30846	0.889330354
7	3	51705	0.595985281
7	4	11117	0.735904371
7	1	84034	0.801277329
7	1	2022	0.548661031
7	6	2027	0.383421355
7	4	2028	0.89045809
7	7	5168	0.643535771
7	2	5169	0.944558541
7	6	22875	0.649102925
7	3	953	0.800368708
7	5	8320	0.532144863
7	6	2033	0.894313214
7	2	2056	0.948476607
7	3	2057	0.447622626
7	6	2059	0.840099244

7	2	54869	0.923943187
7	7	94240	0.676285395
7	2	2081	0.902870429
7	3	90952	0.831854534
7	6	2113	0.828371989
7	6	2117	0.8933759
7	7	51513	0.658900299
7	1	2123	0.736678383
7	1	2124	0.799798495
7	2	2125	0.94130275
7	4	2162	0.463261617
7	3	2149	0.707647778
7	1	2153	0.793959571
7	6	2170	0.878318961
7	5	54491	0.532446294
7	2	11170	0.947987986
7	6	25817	0.530341905
7	1	115572	0.468859996
7	4	54855	0.874317335
7	6	81553	0.776462353
7	5	286336	0.501945136
7	2	84985	0.948172847
7	2	339145	0.945899585
7	4	2191	0.906899115
7	4	355	0.889638327
7	5	356	0.532899417
7	6	79633	0.869383105
7	3	2199	0.526973504
7	4	10516	0.890046896
7	4	2200	0.885372313
7	5	2203	0.585243585
7	4	23194	0.893332073
7	7	26270	0.689202806
7	2	2204	0.945083392
7	7	2205	0.584187805
7	1	2207	0.497376138
7	1	2208	0.79155932
7	5	8857	0.571680138
7	5	2209	0.588802533
7	5	2212	0.565740882
7	5	2213	0.586435947
7	5	2214	0.581380008
7	5	2215	0.593583264
7	5	2217	0.439882887
7	5	2219	0.583210675
7	2	115350	0.942687478
7	2	79368	0.943352665
7	2	115352	0.94576436
7	2	83416	0.955355792
7	5	343413	0.536294239
7	5	221472	0.576652248
7	1	89846	0.786245831

7	3	152273	0.824016482
7	2	2259	0.944656864
7	4	2252	0.692794165
7	5	10875	0.57453755
7	5	2268	0.420764513
7	3	9457	0.617106134
7	1	51303	0.518653748
7	6	51661	0.655319169
7	3	2313	0.598981895
7	3	2321	0.817599267
7	2	2322	0.941858401
7	7	2323	0.597349183
7	3	2324	0.835400314
7	5	752	0.595608743
7	3	91010	0.814731652
7	4	2331	0.876815056
7	3	2335	0.368237735
7	5	23048	0.486327326
7	4	84624	0.879625438
7	1	2350	0.60706039
7	5	50943	0.540586817
7	5	2357	0.592391156
7	6	2487	0.890883563
7	7	6624	0.506935724
7	6	11167	0.819432721
7	2	10272	0.298511288
7	5	2517	0.59732061
7	5	2529	0.595222115
7	6	2534	0.61330316
7	3	8322	0.548943471
7	2	139716	0.945187198
7	4	130589	0.404368209
7	5	8522	0.588469127
7	2	2623	0.945965732
7	3	2624	0.760521846
7	5	2625	0.321254825
7	6	2628	0.82906897
7	1	26301	0.787002914
7	7	2633	0.601539022
7	1	2634	0.437975047
7	5	115361	0.524979783
7	5	115362	0.351013489
7	4	8200	0.850044441
7	5	2672	0.544951864
7	2	2676	0.945686562
7	1	2678	0.799127103
7	6	2690	0.852743924
7	3	51738	0.349253029
7	5	170575	0.578733728
7	5	26157	0.477946323
7	5	55303	0.57713821
7	5	55340	0.584896355

7	3	474344	0.557465474
7	5	168537	0.567918799
7	3	155038	0.638451674
7	3	126326	0.823199612
7	6	9815	0.890484651
7	3	2701	0.817274095
7	3	2702	0.826217078
7	4	2706	0.893188781
7	2	2707	0.948371021
7	6	2709	0.878755773
7	4	169792	0.797024521
7	1	2745	0.800807782
7	4	83468	0.892430972
7	1	9535	0.643668441
7	1	51291	0.570047902
7	7	2766	0.649965067
7	1	2769	0.661380595
7	1	2771	0.707911439
7	3	2791	0.616052821
7	3	54331	0.531245623
7	2	2788	0.848982933
7	5	2793	0.53698907
7	5	10578	0.536987433
7	1	2799	0.743764213
7	5	151306	0.598120485
7	2	2262	0.947526853
7	6	10082	0.350688019
7	5	29933	0.488917055
7	1	7107	0.802825475
7	2	353345	0.937095931
7	6	80045	0.564080079
7	7	29909	0.320282621
7	5	84636	0.535353702
7	5	2841	0.541205104
7	5	2848	0.534487766
7	5	2857	0.529167278
7	2	2859	0.948199905
7	3	2828	0.825974651
7	5	9290	0.570029645
7	5	8477	0.59390797
7	2	27201	0.513620365
7	2	27197	0.948098371
7	5	53831	0.592788724
7	1	63940	0.573407598
7	3	10750	0.827060008
7	5	9402	0.534107613
7	4	26585	0.877650814
7	2	116443	0.796910474
7	6	2957	0.882797043
7	6	2977	0.890032716
7	6	2982	0.686985936
7	5	2995	0.579153931

7	5	3001	0.512063832
7	5	3002	0.469220227
7	5	2999	0.531598634
7	5	3003	0.528225973
7	5	3004	0.541347877
7	5	57817	0.534572443
7	4	145864	0.890864064
7	2	26762	0.936471848
7	5	84868	0.480939509
7	5	3055	0.43047298
7	5	3059	0.590420945
7	7	10866	0.362808583
7	5	10870	0.584807913
7	5	3067	0.280666855
7	3	57520	0.725717178
7	4	9843	0.881498624
7	7	55008	0.708952376
7	7	9709	0.676473585
7	3	26508	0.826259625
7	7	3077	0.694941355
7	4	3082	0.888057381
7	4	3090	0.890226331
7	6	10114	0.901146043
7	7	3012	0.432694709
7	6	8969	0.901967951
7	5	8336	0.308614228
7	7	8357	0.348875793
7	5	3101	0.528815806
7	5	3105	0.555821226
7	5	3106	0.4039545
7	7	3107	0.653215367
7	5	3108	0.578493687
7	5	3109	0.580225729
7	5	3111	0.542342945
7	5	3112	0.392786472
7	5	3113	0.582532272
7	5	3115	0.585605555
7	5	3116	0.520260423
7	5	3117	0.593622423
7	5	3118	0.520532355
7	5	3119	0.589389487
7	5	3120	0.571696437
7	5	3122	0.598824305
7	5	3123	0.578673828
7	5	3127	0.58429697
7	5	3128	0.58273486
7	5	3133	0.612649148
7	5	3134	0.552448456
7	5	3135	0.401198806
7	7	3136	0.642158004
7	4	83872	0.896553323
7	7	3176	0.629226695

7	1	3274	0.671530947
7	3	9957	0.401211145
7	1	9956	0.611790213
7	7	3290	0.653276366
7	2	84941	0.314626466
7	6	3320	0.888642918
7	6	3326	0.889371052
7	4	116835	0.888323553
7	5	3303	0.412153891
7	2	3304	0.737748632
7	5	3305	0.262927687
7	6	3306	0.720785105
7	6	3308	0.887692581
7	6	3309	0.871677075
7	5	3310	0.377000155
7	6	3312	0.896387893
7	4	3356	0.894823432
7	1	94031	0.742890354
7	2	203100	0.945512523
7	2	54768	0.945278826
7	1	3383	0.49492004
7	3	3384	0.830119747
7	1	3385	0.408810038
7	6	22858	0.900278172
7	5	29851	0.53502659
7	4	23308	0.352178029
7	7	3429	0.722874607
7	1	10437	0.606782495
7	7	3430	0.709699709
7	7	10561	0.672031505
7	7	10964	0.745882981
7	7	64135	0.75245665
7	7	3433	0.739864246
7	7	3437	0.722762183
7	6	24138	0.814564887
7	7	8519	0.715808551
7	7	10410	0.691326555
7	6	3454	0.887062554
7	7	3455	0.615969778
7	5	3458	0.545994493
7	6	3459	0.772001976
7	7	3460	0.676433195
7	2	3467	0.945406885
7	3	3479	0.569203972
7	4	285313	0.887436528
7	1	84966	0.800514953
7	5	10261	0.543780169
7	5	3586	0.588790099
7	5	3587	0.578454549
7	7	3588	0.693438589
7	2	3589	0.800641451
7	6	3590	0.616193259

7	7	3592	0.536073909
7	2	3593	0.943851699
7	5	3594	0.538501267
7	2	3595	0.924726281
7	2	3596	0.699617766
7	4	3597	0.804367219
7	5	3600	0.283007536
7	7	3601	0.669326816
7	4	3603	0.243547636
7	6	27190	0.895025009
7	6	55540	0.889715425
7	5	3606	0.540860188
7	7	10068	0.649401665
7	2	8809	0.779502044
7	2	8807	0.790139663
7	2	29949	0.94932333
7	2	3552	0.901761474
7	5	3553	0.578678688
7	4	3554	0.760778878
7	3	7850	0.664230472
7	2	3556	0.946488649
7	2	9173	0.946746779
7	5	3557	0.549546734
7	2	53832	0.861799768
7	1	50615	0.789456712
7	7	58985	0.702325318
7	5	51561	0.218858805
7	2	149233	0.868061205
7	2	11009	0.469909411
7	5	246778	0.564240455
7	5	3559	0.562452555
7	5	3560	0.500838839
7	5	3561	0.527828517
7	5	9235	0.54840227
7	3	3563	0.842464575
7	2	3565	0.946031886
7	7	259307	0.699502121
7	1	3566	0.461911457
7	2	3568	0.946432152
7	5	3569	0.349459871
7	6	3570	0.85208103
7	6	3572	0.870655803
7	2	3574	0.768727083
7	2	3575	0.682717685
7	5	3581	0.321341469
7	4	3624	0.835182039
7	3	3625	0.799711447
7	2	3626	0.946037801
7	4	83729	0.890385294
7	2	11185	0.949064155
7	1	3635	0.652670148
7	2	10788	0.657150656

7	7	3659	0.429474611
7	5	3662	0.294869997
7	7	3665	0.719848656
7	5	3394	0.572749415
7	7	3669	0.661949994
7	4	3671	0.888679173
7	4	22801	0.891014039
7	3	3672	0.833505914
7	2	3674	0.948187055
7	6	3676	0.659186674
7	4	3678	0.880889074
7	6	8516	0.842975027
7	3	3680	0.612427227
7	2	3681	0.946699151
7	5	3683	0.52873831
7	5	3684	0.576611448
7	1	3687	0.790121019
7	6	3688	0.785775937
7	5	3689	0.576368782
7	1	3690	0.786741383
7	5	3695	0.553006519
7	4	9358	0.877195053
7	2	3702	0.90519992
7	3	9452	0.671013479
7	2	3713	0.756833445
7	6	3717	0.7810743
7	5	3718	0.479353223
7	5	152789	0.321335949
7	6	58494	0.885619967
7	6	83700	0.891831014
7	6	133746	0.901978813
7	5	126306	0.245251927
7	3	3728	0.803592338
7	5	3738	0.355157757
7	7	8514	0.470710961
7	2	3751	0.950762351
7	2	23704	0.942745511
7	2	3757	0.768825981
7	2	3766	0.326245766
7	3	3764	0.440659844
7	5	56659	0.508293149
7	2	9424	0.949858834
7	2	3779	0.954442637
7	4	3782	0.745162247
7	1	3783	0.76668867
7	2	343450	0.661538753
7	6	115207	0.86068456
7	3	3791	0.828800386
7	6	57670	0.898599509
7	7	85449	0.594095141
7	2	23046	0.886842536
7	5	3805	0.522161153

7	5	3811	0.524011547
7	2	3812	0.39758859
7	6	3815	0.695826579
7	6	4254	0.722056298
7	3	9365	0.70637158
7	6	55175	0.885386595
7	6	151230	0.89562244
7	1	89857	0.492742361
7	2	5650	0.944826531
7	5	3820	0.539920953
7	2	3821	0.943974807
7	4	3822	0.496763565
7	1	3823	0.796552751
7	5	8302	0.371903541
7	2	3824	0.946436466
7	2	10219	0.919615714
7	2	22914	0.399516582
7	1	8564	0.752216399
7	5	3848	0.434448463
7	4	3861	0.777986082
7	4	3868	0.528328429
7	2	3853	0.768410396
7	7	3854	0.69090914
7	2	3898	0.943518663
7	5	3902	0.568050425
7	5	3903	0.59385324
7	5	3904	0.521295034
7	6	3908	0.847241491
7	3	3910	0.670647258
7	2	3918	0.944669628
7	7	51056	0.686104025
7	1	7805	0.794974143
7	1	27040	0.794714242
7	1	7462	0.797890701
7	6	9113	0.885069836
7	6	26524	0.864423819
7	2	54900	0.466264518
7	5	3932	0.535868906
7	2	414332	0.949113407
7	1	3936	0.769393876
7	5	3937	0.588101719
7	3	9079	0.836730037
7	2	3952	0.915888521
7	1	3953	0.439214542
7	7	3957	0.617877332
7	5	3965	0.584888777
7	4	55203	0.891051769
7	5	5641	0.577413013
7	6	10184	0.863138334
7	1	3976	0.71764076
7	6	3977	0.766261485
7	6	3980	0.897390269

7	5	11024	0.579081968
7	5	11027	0.579331487
7	2	23547	0.34991455
7	2	353514	0.854755814
7	1	79168	0.516111621
7	5	10859	0.588572398
7	5	10288	0.596052199
7	5	11025	0.587299331
7	1	11006	0.767242711
7	5	10990	0.585957802
7	1	80774	0.468986631
7	5	54923	0.538495216
7	6	3987	0.818551615
7	7	3988	0.609820785
7	3	25802	0.642988113
7	6	22853	0.891510231
7	4	4017	0.892579189
7	7	84695	0.584481654
7	6	4023	0.840729064
7	5	9404	0.586809214
7	6	57631	0.896281102
7	5	4033	0.392549146
7	6	4040	0.885537303
7	4	131578	0.831731301
7	4	10234	0.656142673
7	5	126364	0.582192152
7	4	2615	0.88932465
7	2	4045	0.940987141
7	4	4046	0.896984738
7	5	7940	0.591261092
7	5	4049	0.544947752
7	5	4050	0.569818869
7	4	4053	0.753380132
7	4	4055	0.335395207
7	5	4056	0.553939396
7	4	4060	0.879996281
7	5	9450	0.573093682
7	2	4063	0.398487212
7	5	23643	0.581204646
7	5	4066	0.572850134
7	7	4067	0.452960795
7	3	27076	0.588332178
7	2	284348	0.945107588
7	5	4069	0.525256204
7	6	54551	0.689787639
7	4	4121	0.698484631
7	6	10905	0.862263341
7	2	57134	0.815902557
7	5	4129	0.250086836
7	2	440738	0.942418051
7	6	10746	0.897423175
7	2	11184	0.426152319

7	5	8685	0.55777487
7	6	55796	0.863642562
7	2	255231	0.922699094
7	3	1E+08	0.615284124
7	6	4208	0.888140139
7	3	4223	0.585257739
7	6	4233	0.89202648
7	6	4238	0.880054806
7	4	4239	0.888082399
7	5	4242	0.605696066
7	2	83552	0.946588306
7	1	84179	0.795403034
7	1	11320	0.651043113
7	6	4249	0.859942332
7	4	4256	0.888907505
7	4	9645	0.880979212
7	6	4277	0.681893764
7	2	4286	0.515017727
7	1	79083	0.795260372
7	2	4321	0.943993999
7	4	4312	0.889589314
7	4	4323	0.892550843
7	6	4325	0.751606962
7	4	4313	0.895324849
7	2	64386	0.768347176
7	7	4314	0.563660566
7	1	4318	0.766710967
7	3	22915	0.81972787
7	3	79812	0.817692159
7	5	4332	0.589085604
7	5	219972	0.579060415
7	2	4352	0.941603193
7	5	4354	0.432631678
7	7	3140	0.71153015
7	4	116535	0.891693115
7	5	83876	0.516361828
7	7	51264	0.548844349
7	5	128308	0.449546799
7	1	6183	0.486379978
7	7	54460	0.678662009
7	2	10335	0.499205279
7	5	931	0.508060791
7	2	2206	0.945879151
7	5	51338	0.586789671
7	5	64231	0.570145459
7	5	58475	0.509655482
7	5	4481	0.581202526
7	6	253827	0.831285586
7	4	9961	0.816267089
7	7	4599	0.714175889
7	6	4084	0.896379133
7	1	54587	0.521436222

7	3	80177	0.856677235
7	2	50804	0.943800424
7	5	4542	0.572973917
7	5	64005	0.585172139
7	5	4647	0.589725965
7	6	4649	0.824874624
7	6	55728	0.878250935
7	7	10004	0.571755234
7	4	4675	0.898511177
7	5	256236	0.535900919
7	6	26960	0.877704999
7	2	65065	0.797494103
7	5	653361	0.59736515
7	5	4688	0.574642934
7	5	4689	0.566080106
7	6	10499	0.900531271
7	2	9437	0.94429709
7	5	259197	0.534191479
7	2	257194	0.948618924
7	5	150372	0.584292621
7	4	4773	0.825722714
7	1	4791	0.708068593
7	1	4794	0.797022491
7	6	4800	0.888792216
7	6	4801	0.894962836
7	6	4802	0.894445536
7	6	374354	0.899393403
7	4	22795	0.885273606
7	5	4818	0.54694945
7	7	9111	0.75041985
7	3	4855	0.829696225
7	3	4858	0.582536781
7	3	50507	0.556745414
7	5	10062	0.578591866
7	2	2494	0.943375239
7	3	8829	0.815798306
7	2	9369	0.945987566
7	4	4907	0.869540052
7	5	84628	0.505421291
7	4	4914	0.89289562
7	7	4938	0.722456889
7	7	4939	0.73527523
7	7	4940	0.706647371
7	7	8638	0.712142493
7	6	79627	0.817116436
7	4	4969	0.889882629
7	7	283298	0.596638359
7	4	25903	0.889645431
7	4	56944	0.82542828
7	2	4973	0.464466895
7	6	4958	0.726427686
7	1	126014	0.795262902

7	5	5008	0.591307154
7	4	9180	0.880726657
7	1	146183	0.585736393
7	2	5017	0.941845347
7	2	5023	0.944030499
7	7	5025	0.680404787
7	2	5026	0.379282451
7	7	5027	0.645710415
7	5	27334	0.539258464
7	5	5032	0.500050139
7	3	64805	0.554038061
7	3	53829	0.406057782
7	2	9934	0.445125548
7	3	286530	0.633576606
7	4	283208	0.89120735
7	7	140886	0.479043726
7	7	11240	0.455378589
7	6	5049	0.895811704
7	2	55824	0.752027971
7	6	445815	0.865887425
7	7	64761	0.703572751
7	7	54625	0.706585484
7	2	165631	0.935891588
7	7	83666	0.7615102
7	5	64098	0.587265818
7	6	80714	0.877130917
7	3	51294	0.821360421
7	3	27253	0.627674528
7	6	54510	0.807521096
7	7	26025	0.551308103
7	4	5118	0.678362733
7	5	5133	0.530888222
7	4	80380	0.5411817
7	2	5136	0.946801916
7	2	5153	0.355018167
7	4	5139	0.896901953
7	2	5140	0.941997524
7	3	5142	0.603392508
7	5	5148	0.456617809
7	2	5154	0.946755065
7	3	5155	0.815605114
7	6	56034	0.889945561
7	6	5156	0.5401631
7	4	5159	0.888775506
7	4	5157	0.884317788
7	2	64714	0.777335334
7	1	2923	0.524407733
7	2	10158	0.632804651
7	4	23024	0.890530509
7	2	5178	0.802678081
7	7	5202	0.672620627
7	2	5239	0.927863033

7	2	221692	0.949197868
7	1	118788	0.775066746
7	1	5294	0.629657615
7	5	23533	0.58254719
7	5	29992	0.59160057
7	5	11040	0.428383827
7	5	9033	0.533365461
7	2	93035	0.946908104
7	5	5570	0.558431792
7	4	11187	0.83806765
7	2	51365	0.906873162
7	2	26279	0.947215685
7	2	123745	0.943824484
7	7	7941	0.567004096
7	3	219348	0.815930128
7	1	5330	0.795860459
7	6	5332	0.88816
7	6	5334	0.730946539
7	6	23228	0.809999508
7	5	122618	0.569772775
7	1	26499	0.802533259
7	5	5341	0.506812694
7	7	84069	0.408355582
7	4	51177	0.534507244
7	3	83483	0.828495592
7	4	57125	0.805952475
7	4	10154	0.883116552
7	3	23129	0.76304371
7	7	5371	0.726771129
7	4	5376	0.898388638
7	6	10687	0.656051425
7	1	5368	0.795796359
7	4	127435	0.892514404
7	4	10631	0.891110989
7	2	5450	0.947491242
7	5	5452	0.580960284
7	5	5473	0.525182692
7	1	8499	0.700100292
7	2	5493	0.75488899
7	1	57460	0.751835945
7	1	132160	0.587723165
7	4	10848	0.565924096
7	3	26051	0.824378796
7	2	55607	0.943938499
7	2	84106	0.944934444
7	7	5549	0.49028915
7	1	57580	0.794864977
7	5	5551	0.539144884
7	7	5553	0.666112231
7	6	5576	0.900977124
7	6	5577	0.834590527
7	5	5588	0.336574545

7	6	5592	0.887878578
7	6	5617	0.842612781
7	2	5618	0.626505591
7	7	10544	0.62088054
7	2	8842	0.949133103
7	4	5396	0.88529335
7	2	83886	0.944737186
7	2	283659	0.741054608
7	1	5660	0.801438775
7	5	5699	0.568008431
7	7	5696	0.655503051
7	7	5698	0.674655849
7	6	5700	0.868948511
7	6	5701	0.844063811
7	7	5702	0.677987094
7	7	5704	0.526456188
7	5	5705	0.290169706
7	6	5706	0.887322953
7	6	5716	0.883567437
7	6	5717	0.892840939
7	7	5719	0.698925394
7	6	10213	0.800464162
7	2	5707	0.77815877
7	6	5708	0.878342453
7	7	5709	0.54325723
7	7	5710	0.569176471
7	6	5711	0.895555744
7	6	9861	0.887539614
7	7	5713	0.705382037
7	7	5714	0.734635775
7	5	5720	0.373105331
7	5	5721	0.419204667
7	6	10197	0.893801613
7	1	9051	0.794597271
7	5	5724	0.57616148
7	5	171558	0.514650661
7	5	5729	0.335660561
7	4	5730	0.375233434
7	4	5732	0.888855873
7	4	5737	0.888462134
7	2	5739	0.945856768
7	6	5740	0.778252837
7	1	26191	0.791651842
7	5	5777	0.534493067
7	1	5778	0.525223443
7	3	5787	0.578080048
7	1	5788	0.471448855
7	5	5790	0.55116517
7	1	5795	0.791467957
7	3	5797	0.82020469
7	1	5800	0.760859744
7	6	5817	0.839169846

7	2	149628	0.340776145
7	7	23475	0.711571958
7	5	55647	0.571222825
7	4	9363	0.822786351
7	5	326624	0.31857278
7	6	116442	0.302683621
7	6	115273	0.564527246
7	6	51762	0.864652785
7	6	5887	0.898105765
7	2	23132	0.880276732
7	2	135250	0.946566794
7	4	353091	0.844864604
7	2	154064	0.947783973
7	3	10742	0.697367094
7	3	10268	0.544356017
7	6	51735	0.896100733
7	4	5919	0.889683578
7	7	5920	0.684749029
7	3	5924	0.58835546
7	3	10235	0.821971938
7	2	25780	0.943517508
7	1	115727	0.546662759
7	3	51285	0.79582706
7	1	9770	0.529942508
7	6	283349	0.897002765
7	1	83937	0.797499121
7	5	83593	0.506164694
7	2	166824	0.94455001
7	4	83758	0.887714371
7	7	57333	0.473968855
7	5	92241	0.542935305
7	2	145226	0.946672847
7	6	8434	0.75766339
7	6	5966	0.88693392
7	1	5971	0.357612324
7	2	5649	0.941902256
7	5	5973	0.472186071
7	6	5978	0.921910118
7	6	5993	0.888106742
7	5	8625	0.423314288
7	6	5994	0.894293811
7	6	23179	0.799274092
7	4	6003	0.544385343
7	5	5996	0.520465535
7	5	64407	0.567327115
7	3	8490	0.583600497
7	2	399	0.942909399
7	4	57381	0.473484501
7	6	55183	0.895166966
7	4	9610	0.89709337
7	1	79890	0.796655252
7	5	6035	0.593199212

7	5	6036	0.580837368
7	1	6039	0.671769817
7	4	84659	0.665125613
7	2	54941	0.942207682
7	5	115992	0.58335057
7	3	285671	0.786520903
7	3	54538	0.83836605
7	6	9475	0.898298367
7	4	4919	0.882603534
7	7	8986	0.522211288
7	7	91543	0.715298932
7	1	6252	0.752702076
7	7	64108	0.718938268
7	4	862	0.788293582
7	2	864	0.54559973
7	2	6283	0.938483215
7	4	140576	0.895037938
7	4	6273	0.888569427
7	5	6279	0.589767429
7	5	6280	0.578221159
7	7	6285	0.616481295
7	6	6297	0.883239159
7	3	201191	0.80446431
7	2	154075	0.948363025
7	7	54809	0.711698969
7	7	219285	0.724394037
7	5	25939	0.588603235
7	5	64092	0.594458128
7	2	1757	0.94721952
7	6	55206	0.888323584
7	4	374897	0.889612207
7	1	8578	0.668423962
7	7	91179	0.455790619
7	2	8796	0.948202264
7	2	256380	0.404414399
7	2	6332	0.947557132
7	7	222663	0.495918869
7	1	6383	0.357110322
7	2	27111	0.279715622
7	5	10993	0.594044049
7	5	113675	0.54202883
7	6	9871	0.857130323
7	5	6398	0.479333495
7	7	8991	0.689941513
7	1	6402	0.757113248
7	5	6403	0.501580028
7	1	6404	0.795947226
7	6	256987	0.858098373
7	5	5265	0.587079373
7	4	5054	0.891754027
7	4	5176	0.82602471
7	7	710	0.459945343

7	5	57713	0.46064656
7	2	2810	0.741565231
7	4	6423	0.638306567
7	2	6439	0.951375185
7	6	6444	0.832516965
7	2	84251	0.944742243
7	5	4068	0.534434658
7	2	9047	0.90791517
7	3	10044	0.831355165
7	2	400745	0.826823427
7	3	126669	0.80255618
7	5	89790	0.574175498
7	5	114132	0.334286837
7	5	89858	0.535911375
7	5	8778	0.59542398
7	2	946	0.948089146
7	5	27036	0.590248864
7	2	27181	0.902062705
7	5	27180	0.587220754
7	2	10326	0.46873348
7	5	284759	0.589378095
7	5	27240	0.538611759
7	5	84174	0.50612181
7	5	6503	0.569301041
7	5	6504	0.498897339
7	1	114836	0.592258947
7	1	57823	0.795682791
7	5	56833	0.601776519
7	2	6556	0.410589218
7	2	6559	0.945549728
7	5	51296	0.600423763
7	6	6571	0.908345998
7	4	6512	0.893879696
7	2	123041	0.948042963
7	1	55315	0.697646086
7	5	6518	0.501046296
7	2	10568	0.950702798
7	2	29986	0.945564197
7	1	85414	0.793994195
7	2	6539	0.904340035
7	5	9056	0.591757463
7	6	6546	0.90148568
7	5	11309	0.572465936
7	2	81796	0.778163882
7	3	9353	0.832250123
7	1	6586	0.498578003
7	2	27293	0.943553054
7	5	333929	0.564613697
7	4	25992	0.883165627
7	7	6636	0.480141077
7	4	6641	0.87430801
7	4	6649	0.895228935

7	2	6651	0.546526708
7	3	64321	0.821056061
7	2	6660	0.931220405
7	7	6672	0.699900965
7	7	3431	0.692176063
7	5	11262	0.554616261
7	4	6676	0.85154075
7	1	6678	0.413339315
7	3	8404	0.621660785
7	6	221178	0.898532678
7	1	6688	0.795190376
7	2	6689	0.941713514
7	5	6693	0.464265658
7	5	9806	0.54207172
7	4	10418	0.777917672
7	3	10252	0.817766682
7	2	6753	0.944580805
7	6	6483	0.473901178
7	1	8869	0.51363125
7	1	10402	0.784334826
7	5	6480	0.509896811
7	2	256435	0.949124281
7	6	7903	0.51550706
7	5	23166	0.582778571
7	5	246329	0.559424589
7	6	90627	0.858588318
7	2	9754	0.94316039
7	7	6772	0.737012146
7	2	6775	0.928185359
7	7	6776	0.623459365
7	6	9262	0.839845056
7	2	65975	0.942162541
7	6	6801	0.862538545
7	4	29091	0.869853503
7	1	56670	0.796938471
7	7	23213	0.562695448
7	2	6819	0.867952829
7	2	6820	0.946476918
7	5	203328	0.595841634
7	4	23345	0.87883558
7	6	23208	0.874678392
7	4	94120	0.815644694
7	5	117289	0.592717318
7	6	57551	0.885743156
7	7	6890	0.736167759
7	7	6891	0.699407609
7	7	6892	0.631281066
7	1	55080	0.591472189
7	5	374403	0.54444263
7	5	30009	0.553461936
7	3	6915	0.835701679
7	1	6916	0.786944586

7	6	56849	0.880036904
7	1	10312	0.796145154
7	5	8115	0.510687668
7	7	6948	0.642269712
7	3	7010	0.823932066
7	3	54997	0.617054431
7	1	22797	0.769823114
7	1	7040	0.760469667
7	6	7042	0.528450668
7	4	7043	0.890710255
7	4	7045	0.890891099
7	6	7046	0.84812455
7	3	7048	0.835313229
7	6	9392	0.894418267
7	7	7051	0.429925982
7	1	7052	0.779445969
7	6	7057	0.751988963
7	4	7058	0.892929209
7	6	7066	0.792165797
7	4	7070	0.841538548
7	3	7075	0.817133152
7	5	497189	0.546196268
7	5	91937	0.30963936
7	2	92609	0.935942991
7	4	7077	0.881193228
7	4	7078	0.887991814
7	2	81793	0.902418114
7	5	7096	0.524795698
7	5	7099	0.433998691
7	2	7100	0.942358607
7	5	51284	0.575245777
7	5	51311	0.586389004
7	3	54106	0.324134539
7	3	116441	0.841720921
7	5	53346	0.564810838
7	5	147138	0.561725681
7	7	113277	0.691591719
7	6	338773	0.577054041
7	6	219623	0.882891442
7	6	83604	0.895505043
7	7	84283	0.465050521
7	5	7124	0.59423236
7	4	7130	0.888931161
7	5	79626	0.584232379
7	1	8797	0.610957216
7	6	8795	0.864098912
7	1	8794	0.567305991
7	4	8793	0.891616586
7	1	8792	0.798088022
7	7	4982	0.619416033
7	4	51330	0.887338354
7	5	23495	0.452118857

7	2	115650	0.945187762
7	1	8764	0.795968214
7	2	608	0.904354452
7	5	8784	0.253687805
7	4	7132	0.791588061
7	5	7133	0.564190371
7	3	8718	0.433366385
7	3	7293	0.437557243
7	1	8771	0.759514297
7	5	943	0.283871821
7	1	3604	0.689109678
7	7	8743	0.719308259
7	1	8600	0.720992092
7	5	8742	0.454834424
7	5	407977	0.566974321
7	5	8741	0.588649808
7	5	10673	0.587050208
7	2	8740	0.420459849
7	2	9966	0.62608152
7	7	8995	0.728195989
7	4	7292	0.833515187
7	5	944	0.583334079
7	7	8744	0.693243299
7	6	23043	0.889406958
7	2	79931	0.944687426
7	6	63923	0.536017502
7	2	7139	0.944991154
7	6	64759	0.795019674
7	6	9760	0.818696921
7	2	27010	0.946768136
7	4	7177	0.846987474
7	4	23430	0.306607501
7	2	25823	0.704888186
7	5	7185	0.272651828
7	5	80342	0.530929215
7	2	50852	0.397718584
7	5	54210	0.389424593
7	5	54209	0.584663498
7	5	340205	0.582804133
7	7	6737	0.74646006
7	7	10346	0.618272809
7	2	391712	0.944682186
7	6	26133	0.714289682
7	1	51393	0.593000229
7	2	162514	0.778453588
7	6	7253	0.885388699
7	2	85480	0.289820712
7	1	441631	0.75202924
7	5	10077	0.42632452
7	1	7106	0.423714936
7	6	146057	0.86712442
7	2	158248	0.9498621

7	6	79809	0.890210278
7	4	84617	0.894950794
7	2	7294	0.946694994
7	1	7305	0.537317971
7	2	53347	0.324579177
7	5	10537	0.516128541
7	7	9246	0.712895401
7	2	197131	0.76422093
7	5	7351	0.593867459
7	6	127933	0.892316766
7	6	80329	0.890173038
7	4	80328	0.811295409
7	6	79465	0.904016749
7	5	201294	0.478498063
7	6	8633	0.89547927
7	1	81622	0.744761772
7	2	83878	0.915466542
7	6	219333	0.896803569
7	6	158880	0.890374651
7	1	10911	0.732783108
7	7	10791	0.672723783
7	3	22846	0.696217103
7	5	7409	0.585808783
7	4	7412	0.894581863
7	7	7423	0.4827317
7	4	7424	0.573159132
7	2	27287	0.394696677
7	2	389136	0.822154899
7	4	7431	0.891000405
7	5	284013	0.601278503
7	2	8875	0.403585061
7	5	29802	0.544429838
7	4	155382	0.824349997
7	5	11326	0.599610971
7	3	7450	0.648258082
7	7	7453	0.657245022
7	5	7454	0.560493672
7	2	128488	0.950578982
7	6	8840	0.866868143
7	4	7472	0.719298797
7	6	7494	0.780178547
7	5	6375	0.457131831
7	5	6846	0.509013781
7	6	2829	0.743264831
7	1	55113	0.386741411
7	4	7512	0.878825344
7	5	7535	0.532945735
7	2	81030	0.406211476
7	6	65986	0.884002243
7	2	27033	0.928539711
7	6	253832	0.88899688
7	6	23414	0.889911613

7	6	7586	0.845465358
7	5	84225	0.495935772
7	4	7739	0.822976205
7	2	7762	0.942185102
