# Supplementary material for: Analysis of Immune Gene Expression Subtypes Reveals Osteosarcoma Immune Heterogeneity
Source: J Oncol. 2021 Mar 1;2021:6649412. doi: 10.1155/2021/6649412 (PMC7939746; doi:10.1155/2021/6649412)
Supplement: Supplementary Materials — Supplementary Figure S1. Consensus matrix for immune-related gene modules derived from consensus clustering analysis. Supplementary Figure S2. Expression feature of immune subtypes in training TARGET cohort. Supplementary Figure S3. The validation of immune subtypes in the independent GEO cohort. Supplementary Figure S4. The intracluster heterogeneity revealed by the immune landscape analysis and different subcluster molecular characteristics of patients in immune subtypes 1 and 4 are shown. Supplementary Table S1. The bulk RNA-seq data retrieved from the OS-project. Supplementary Table S2. The collected 1989 immune-related genes. Supplementary Table S3. The immune subtype annotation at the patient level in the TARGET cohort. Supplementary Table S4. The immune-related gene modules feature. Supplementary Table S5. Functional enrichment analysis of gene modules. Supplementary Table S6. The immune-related molecular features of the TARGET patients. Supplementary Table S7. Correlation of average gene module scores between molecular characteristics and immune subtypes. [file 6649412.f1.zip › 6649412.f1/Supplementary Table S5.pdf]

subtype	Category	Term
Phosphoryl	GOTERM_BP_DIRECT	GO:0035335~peptidyl-tyrosine dephosphorylation
Phosphoryl	GOTERM_BP_DIRECT	GO:0006470~protein dephosphorylation
Phosphoryl	GOTERM_BP_DIRECT	GO:0030225~macrophage differentiation
Phosphoryl	GOTERM_BP_DIRECT	GO:0032496~response to lipopolysaccharide
Phosphoryl	GOTERM_BP_DIRECT	GO:0030168~platelet activation
Phosphoryl	GOTERM_BP_DIRECT	GO:0030316~osteoclast differentiation
Phosphoryl	GOTERM_BP_DIRECT	GO:0042981~regulation of apoptotic process
Phosphoryl	GOTERM_BP_DIRECT	GO:0007229~integrin-mediated signaling pathway
Phosphoryl	GOTERM_BP_DIRECT	GO:0045860~positive regulation of protein kinase activity
Phosphoryl	GOTERM_BP_DIRECT	GO:0032967~positive regulation of collagen biosynthetic process
Phosphoryl	GOTERM_BP_DIRECT	GO:0001934~positive regulation of protein phosphorylation
Phosphoryl	GOTERM_BP_DIRECT	GO:0001503~ossification
Phosphoryl	GOTERM_BP_DIRECT	GO:0033280~response to vitamin D
Differentia	GOTERM_BP_DIRECT	GO:0007268~chemical synaptic transmission
Differentia	GOTERM_BP_DIRECT	GO:0019233~sensory perception of pain
Differentia	GOTERM_BP_DIRECT	GO:0061337~cardiac conduction
Differentia	GOTERM_BP_DIRECT	GO:0070588~calcium ion transmembrane transport
Differentia	GOTERM_BP_DIRECT	GO:0031424~keratinization
Differentia	GOTERM_BP_DIRECT	GO:0034765~regulation of ion transmembrane transport
Angiogene	GOTERM_BP_DIRECT	GO:0001525~angiogenesis
Angiogene	GOTERM_BP_DIRECT	GO:0001569~patterning of blood vessels
Angiogene	GOTERM_BP_DIRECT	GO:0030198~extracellular matrix organization
Angiogene	GOTERM_BP_DIRECT	GO:0003158~endothelium development
Angiogene	GOTERM_BP_DIRECT	GO:0001938~positive regulation of endothelial cell proliferation
Angiogene	GOTERM_BP_DIRECT	GO:0043410~positive regulation of MAPK cascade
Angiogene	GOTERM_BP_DIRECT	GO:0010596~negative regulation of endothelial cell migration
Angiogene	GOTERM_BP_DIRECT	GO:0035924~cellular response to vascular endothelial growth fac
Angiogene	GOTERM_BP_DIRECT	GO:0003151~outflow tract morphogenesis
Angiogene	GOTERM_BP_DIRECT	GO:0045766~positive regulation of angiogenesis
Angiogene	GOTERM_BP_DIRECT	GO:0008015~blood circulation
Angiogene	GOTERM_BP_DIRECT	GO:0009611~response to wounding
Angiogene	GOTERM_BP_DIRECT	GO:0001570~vasculogenesis
Angiogene	GOTERM_BP_DIRECT	GO:0042311~vasodilation
Angiogene	GOTERM_BP_DIRECT	GO:0016525~negative regulation of angiogenesis
Angiogene	GOTERM_BP_DIRECT	GO:0014911~positive regulation of smooth muscle cell migration
Angiogene	GOTERM_BP_DIRECT	GO:0035815~positive regulation of renal sodium excretion
Angiogene	GOTERM_BP_DIRECT	GO:0007599~hemostasis
Angiogene	GOTERM_BP_DIRECT	GO:0048514~blood vessel morphogenesis
Angiogene	GOTERM_BP_DIRECT	GO:0031290~retinal ganglion cell axon guidance
Angiogene	GOTERM_BP_DIRECT	GO:0061299~retina vasculature morphogenesis in camera-type
Angiogene	GOTERM_BP_DIRECT	GO:0090051~negative regulation of cell migration involved in spr
Angiogene	GOTERM_BP_DIRECT	GO:0007156~homophilic cell adhesion via plasma membrane ad
Angiogene	GOTERM_BP_DIRECT	GO:0007507~heart development
Angiogene	GOTERM_BP_DIRECT	GO:0030334~regulation of cell migration
Angiogene	GOTERM_BP_DIRECT	GO:0045907~positive regulation of vasoconstriction
Angiogene	GOTERM_BP_DIRECT	GO:0001974~blood vessel remodeling
Reactive st	GOTERM_BP_DIRECT	GO:0030199~collagen fibril organization
Reactive st	GOTERM_BP_DIRECT	GO:0030198~extracellular matrix organization
Reactive st	GOTERM_BP_DIRECT	GO:0030574~collagen catabolic process
Reactive st	GOTERM_BP_DIRECT	GO:0035987~endodermal cell differentiation
Reactive st	GOTERM_BP_DIRECT	GO:0007155~cell adhesion

Reactive st	GOTERM_BP_DIRECT	GO:0007568~aging
Reactive st	GOTERM_BP_DIRECT	GO:0001501~skeletal system development
Reactive st	GOTERM_BP_DIRECT	GO:0001666~response to hypoxia
Reactive st	GOTERM_BP_DIRECT	GO:0046426~negative regulation of JAK-STAT cascade
Reactive st	GOTERM_BP_DIRECT	GO:0070208~protein heterotrimerization
Reactive st	GOTERM_BP_DIRECT	GO:0043206~extracellular fibril organization
Reactive st	GOTERM_BP_DIRECT	GO:0071230~cellular response to amino acid stimulus
Reactive st	GOTERM_BP_DIRECT	GO:0009612~response to mechanical stimulus
Reactive st	GOTERM_BP_DIRECT	GO:0022617~extracellular matrix disassembly
Reactive st	GOTERM_BP_DIRECT	GO:0007160~cell-matrix adhesion
Reactive st	GOTERM_BP_DIRECT	GO:0032355~response to estradiol
Reactive st	GOTERM_BP_DIRECT	GO:0043408~regulation of MAPK cascade
Reactive st	GOTERM_BP_DIRECT	GO:0001525~angiogenesis
Reactive st	GOTERM_BP_DIRECT	GO:0060395~SMAD protein signal transduction
Reactive st	GOTERM_BP_DIRECT	GO:0016055~Wnt signaling pathway
Reactive st	GOTERM_BP_DIRECT	GO:0060325~face morphogenesis
Reactive st	GOTERM_BP_DIRECT	GO:0030178~negative regulation of Wnt signaling pathway
Reactive st	GOTERM_BP_DIRECT	GO:0042633~hair cycle
Reactive st	GOTERM_BP_DIRECT	GO:0071560~cellular response to transforming growth factor bet
Reactive st	GOTERM_BP_DIRECT	GO:0007601~visual perception
Reactive st	GOTERM_BP_DIRECT	GO:0008284~positive regulation of cell proliferation
Reactive st	GOTERM_BP_DIRECT	GO:0002576~platelet degranulation
Reactive st	GOTERM_BP_DIRECT	GO:0006469~negative regulation of protein kinase activity
T cell	GOTERM_BP_DIRECT	GO:0006955~immune response
T cell	GOTERM_BP_DIRECT	GO:0031295~T cell costimulation
T cell	GOTERM_BP_DIRECT	GO:0006954~inflammatory response
T cell	GOTERM_BP_DIRECT	GO:0002504~antigen processing and presentation of peptide or
T cell	GOTERM_BP_DIRECT	GO:0007186~G-protein coupled receptor signaling pathway
T cell	GOTERM_BP_DIRECT	GO:0050776~regulation of immune response
T cell	GOTERM_BP_DIRECT	GO:0070098~chemokine-mediated signaling pathway
T cell	GOTERM_BP_DIRECT	GO:0042102~positive regulation of T cell proliferation
T cell	GOTERM_BP_DIRECT	GO:0006935~chemotaxis
T cell	GOTERM_BP_DIRECT	GO:0071346~cellular response to interferon-gamma
T cell	GOTERM_BP_DIRECT	GO:0019886~antigen processing and presentation of exogenous
T cell	GOTERM_BP_DIRECT	GO:0006959~humoral immune response
T cell	GOTERM_BP_DIRECT	GO:0030593~neutrophil chemotaxis
T cell	GOTERM_BP_DIRECT	GO:0042130~negative regulation of T cell proliferation
T cell	GOTERM_BP_DIRECT	GO:0007267~cell-cell signaling
T cell	GOTERM_BP_DIRECT	GO:0090023~positive regulation of neutrophil chemotaxis
T cell	GOTERM_BP_DIRECT	GO:0019882~antigen processing and presentation
T cell	GOTERM_BP_DIRECT	GO:0071222~cellular response to lipopolysaccharide
T cell	GOTERM_BP_DIRECT	GO:0031663~lipopolysaccharide-mediated signaling pathway
T cell	GOTERM_BP_DIRECT	GO:0060333~interferon-gamma-mediated signaling pathway
T cell	GOTERM_BP_DIRECT	GO:0045087~innate immune response
T cell	GOTERM_BP_DIRECT	GO:0060326~cell chemotaxis
T cell	GOTERM_BP_DIRECT	GO:0050852~T cell receptor signaling pathway
T cell	GOTERM_BP_DIRECT	GO:0006968~cellular defense response
T cell	GOTERM_BP_DIRECT	GO:0045086~positive regulation of interleukin-2 biosynthetic pro
T cell	GOTERM_BP_DIRECT	GO:0032760~positive regulation of tumor necrosis factor product
T cell	GOTERM_BP_DIRECT	GO:0050729~positive regulation of inflammatory response
T cell	GOTERM_BP_DIRECT	GO:0071347~cellular response to interleukin-1
T cell	GOTERM_BP_DIRECT	GO:0045060~negative thymic T cell selection

T cell	GOTERM_BP_DIRECT	GO:0032729~positive regulation of interferon-gamma productio
T cell	GOTERM_BP_DIRECT	GO:0032689~negative regulation of interferon-gamma producti
T cell	GOTERM_BP_DIRECT	GO:0006952~defense response
T cell	GOTERM_BP_DIRECT	GO:0071356~cellular response to tumor necrosis factor
T cell	GOTERM_BP_DIRECT	GO:0070374~positive regulation of ERK1 and ERK2 cascade
T cell	GOTERM_BP_DIRECT	GO:0032753~positive regulation of interleukin-4 production
T cell	GOTERM_BP_DIRECT	GO:0002548~monocyte chemotaxis
T cell	GOTERM_BP_DIRECT	GO:0007165~signal transduction
T cell	GOTERM_BP_DIRECT	GO:0042110~T cell activation
T cell	GOTERM_BP_DIRECT	GO:0032720~negative regulation of tumor necrosis factor produ
T cell	GOTERM_BP_DIRECT	GO:0042535~positive regulation of tumor necrosis factor biosynt
T cell	GOTERM_BP_DIRECT	GO:0046641~positive regulation of alpha-beta T cell proliferatio
T cell	GOTERM_BP_DIRECT	GO:0001771~immunological synapse formation
T cell	GOTERM_BP_DIRECT	GO:0048247~lymphocyte chemotaxis
T cell	GOTERM_BP_DIRECT	GO:0002690~positive regulation of leukocyte chemotaxis
T cell	GOTERM_BP_DIRECT	GO:0050830~defense response to Gram-positive bacterium
T cell	GOTERM_BP_DIRECT	GO:0006915~apoptotic process
T cell	GOTERM_BP_DIRECT	GO:0042742~defense response to bacterium
T cell	GOTERM_BP_DIRECT	GO:0034142~toll-like receptor 4 signaling pathway
T cell	GOTERM_BP_DIRECT	GO:0097190~apoptotic signaling pathway
T cell	GOTERM_BP_DIRECT	GO:0042346~positive regulation of NF-kappaB import into nucle
T cell	GOTERM_BP_DIRECT	GO:0002250~adaptive immune response
T cell	GOTERM_BP_DIRECT	GO:0051897~positive regulation of protein kinase B signaling
T cell	GOTERM_BP_DIRECT	GO:0032703~negative regulation of interleukin-2 production
T cell	GOTERM_BP_DIRECT	GO:0048246~macrophage chemotaxis
T cell	GOTERM_BP_DIRECT	GO:0090630~activation of GTPase activity
T cell	GOTERM_BP_DIRECT	GO:0032496~response to lipopolysaccharide
T cell	GOTERM_BP_DIRECT	GO:0032735~positive regulation of interleukin-12 production
T cell	GOTERM_BP_DIRECT	GO:0032715~negative regulation of interleukin-6 production
T cell	GOTERM_BP_DIRECT	GO:0050870~positive regulation of T cell activation
T cell	GOTERM_BP_DIRECT	GO:0002523~leukocyte migration involved in inflammatory respo
T cell	GOTERM_BP_DIRECT	GO:0006956~complement activation
T cell	GOTERM_BP_DIRECT	GO:0019835~cytolysis
T cell	GOTERM_BP_DIRECT	GO:0006909~phagocytosis
T cell	GOTERM_BP_DIRECT	GO:0045580~regulation of T cell differentiation
T cell	GOTERM_BP_DIRECT	GO:0045059~positive thymic T cell selection
T cell	GOTERM_BP_DIRECT	GO:0070266~necroptotic process
T cell	GOTERM_BP_DIRECT	GO:0045410~positive regulation of interleukin-6 biosynthetic pro
T cell	GOTERM_BP_DIRECT	GO:0030816~positive regulation of cAMP metabolic process
T cell	GOTERM_BP_DIRECT	GO:0045416~positive regulation of interleukin-8 biosynthetic pro
T cell	GOTERM_BP_DIRECT	GO:0001768~establishment of T cell polarity
T cell	GOTERM_BP_DIRECT	GO:0002503~peptide antigen assembly with MHC class II protei
T cell	GOTERM_BP_DIRECT	GO:0007166~cell surface receptor signaling pathway
T cell	GOTERM_BP_DIRECT	GO:0002224~toll-like receptor signaling pathway
T cell	GOTERM_BP_DIRECT	GO:0050715~positive regulation of cytokine secretion
T cell	GOTERM_BP_DIRECT	GO:0030889~negative regulation of B cell proliferation
T cell	GOTERM_BP_DIRECT	GO:0010818~T cell chemotaxis
T cell	GOTERM_BP_DIRECT	GO:0051044~positive regulation of membrane protein ectodoma
T cell	GOTERM_BP_DIRECT	GO:0032722~positive regulation of chemokine production
T cell	GOTERM_BP_DIRECT	GO:0042088~T-helper 1 type immune response
T cell	GOTERM_BP_DIRECT	GO:0050900~leukocyte migration
T cell	GOTERM_BP_DIRECT	GO:0042832~defense response to protozoan

T cell	GOTERM_BP_DIRECT	GO:0007249~I-kappaB kinase/NF-kappaB signaling
T cell	GOTERM_BP_DIRECT	GO:0002755~MyD88-dependent toll-like receptor signaling patl
T cell	GOTERM_BP_DIRECT	GO:0001916~positive regulation of T cell mediated cytotoxicity
T cell	GOTERM_BP_DIRECT	GO:0002479~antigen processing and presentation of exogenous
TGF-β	GOTERM_BP_DIRECT	GO:0006468~protein phosphorylation
TGF-β	GOTERM_BP_DIRECT	GO:0090090~negative regulation of canonical Wnt signaling patl
TGF-β	GOTERM_BP_DIRECT	GO:0043161~proteasome-mediated ubiquitin-dependent protei
TGF-β	GOTERM_BP_DIRECT	GO:0038061~NIK/NF-kappaB signaling
TGF-β	GOTERM_BP_DIRECT	GO:0070120~ciliary neurotrophic factor-mediated signaling path
TGF-β	GOTERM_BP_DIRECT	GO:0006511~ubiquitin-dependent protein catabolic process
TGF-β	GOTERM_BP_DIRECT	GO:0043488~regulation of mRNA stability
TGF-β	GOTERM_BP_DIRECT	GO:0035556~intracellular signal transduction
TGF-β	GOTERM_BP_DIRECT	GO:0090263~positive regulation of canonical Wnt signaling pathv
TGF-β	GOTERM_BP_DIRECT	GO:0006521~regulation of cellular amino acid metabolic process
TGF-β	GOTERM_BP_DIRECT	GO:0051436~negative regulation of ubiquitin-protein ligase activ
TGF-β	GOTERM_BP_DIRECT	GO:0031145~anaphase-promoting complex-dependent catabol
TGF-β	GOTERM_BP_DIRECT	GO:0051437~positive regulation of ubiquitin-protein ligase activit
TGF-β	GOTERM_BP_DIRECT	GO:0060071~Wnt signaling pathway, planar cell polarity pathway
TGF-β	GOTERM_BP_DIRECT	GO:0006366~transcription from RNA polymerase II promoter
TGF-β	GOTERM_BP_DIRECT	GO:0018107~peptidyl-threonine phosphorylation
TGF-β	GOTERM_BP_DIRECT	GO:0045669~positive regulation of osteoblast differentiation
TGF-β	GOTERM_BP_DIRECT	GO:0002223~stimulatory C-type lectin receptor signaling pathwa
TGF-β	GOTERM_BP_DIRECT	GO:0007399~nervous system development
TGF-β	GOTERM_BP_DIRECT	GO:0003148~outflow tract septum morphogenesis
TGF-β	GOTERM_BP_DIRECT	GO:0035329~hippo signaling
TGF-β	GOTERM_BP_DIRECT	GO:0000165~MAPK cascade
TGF-β	GOTERM_BP_DIRECT	GO:0002479~antigen processing and presentation of exogenous
TGF-β	GOTERM_BP_DIRECT	GO:0006986~response to unfolded protein
TGF-β	GOTERM_BP_DIRECT	GO:0000082~G1/S transition of mitotic cell cycle
TGF-β	GOTERM_BP_DIRECT	GO:0060412~ventricular septum morphogenesis
TGF-β	GOTERM_BP_DIRECT	GO:0023014~signal transduction by protein phosphorylation
TGF-β	GOTERM_BP_DIRECT	GO:0000209~protein polyubiquitination
TGF-β	GOTERM_BP_DIRECT	GO:0018105~peptidyl-serine phosphorylation
TGF-β	GOTERM_BP_DIRECT	GO:0060271~cilium morphogenesis
TGF-β	GOTERM_BP_DIRECT	GO:0030900~forebrain development
TGF-β	GOTERM_BP_DIRECT	GO:0006355~regulation of transcription, DNA-templated
TGF-β	GOTERM_BP_DIRECT	GO:0051301~cell division
TGF-β	GOTERM_BP_DIRECT	GO:0006457~protein folding
TGF-β	GOTERM_BP_DIRECT	GO:0001701~in utero embryonic development
TGF-β	GOTERM_BP_DIRECT	GO:0046777~protein autophosphorylation
TGF-β	GOTERM_BP_DIRECT	GO:0045893~positive regulation of transcription, DNA-templatec
TGF-β	GOTERM_BP_DIRECT	GO:2001237~negative regulation of extrinsic apoptotic signaling
TGF-β	GOTERM_BP_DIRECT	GO:0032956~regulation of actin cytoskeleton organization
TGF-β	GOTERM_BP_DIRECT	GO:0007178~transmembrane receptor protein serine/threonine
TGF-β	GOTERM_BP_DIRECT	GO:0001707~mesoderm formation
TGF-β	GOTERM_BP_DIRECT	GO:0009755~hormone-mediated signaling pathway
TGF-β	GOTERM_BP_DIRECT	GO:0043065~positive regulation of apoptotic process
TGF-β	GOTERM_BP_DIRECT	GO:0038095~Fc-epsilon receptor signaling pathway
TGF-β	GOTERM_BP_DIRECT	GO:0045600~positive regulation of fat cell differentiation
TGF-β	GOTERM_BP_DIRECT	GO:0051726~regulation of cell cycle
TGF-β	GOTERM_BP_DIRECT	GO:0014033~neural crest cell differentiation
TGF-β	GOTERM_BP_DIRECT	GO:0061626~pharyngeal arch artery morphogenesis

TGF-β	GOTERM_BP_DIRECT	GO:0003223~ventricular compact myocardium morphogenesis
TGF-β	GOTERM_BP_DIRECT	GO:0043254~regulation of protein complex assembly
TGF-β	GOTERM_BP_DIRECT	GO:0048568~embryonic organ development
TGF-β	GOTERM_BP_DIRECT	GO:0003183~mitral valve morphogenesis
TGF-β	GOTERM_BP_DIRECT	GO:0045899~positive regulation of RNA polymerase II transcripti
TGF-β	GOTERM_BP_DIRECT	GO:1900034~regulation of cellular response to heat
TGF-β	GOTERM_BP_DIRECT	GO:0048666~neuron development
TGF-β	GOTERM_BP_DIRECT	GO:0016055~Wnt signaling pathway
TGF-β	GOTERM_BP_DIRECT	GO:0030501~positive regulation of bone mineralization
TGF-β	GOTERM_BP_DIRECT	GO:0060021~palate development
TGF-β	GOTERM_BP_DIRECT	GO:0072659~protein localization to plasma membrane
IFN-γ	GOTERM_BP_DIRECT	GO:0060337~type I interferon signaling pathway
IFN-γ	GOTERM_BP_DIRECT	GO:0009615~response to virus
IFN-γ	GOTERM_BP_DIRECT	GO:0051607~defense response to virus
IFN-γ	GOTERM_BP_DIRECT	GO:0060333~interferon-gamma-mediated signaling pathway
IFN-γ	GOTERM_BP_DIRECT	GO:0045071~negative regulation of viral genome replication
IFN-γ	GOTERM_BP_DIRECT	GO:0000209~protein polyubiquitination
IFN-γ	GOTERM_BP_DIRECT	GO:0002474~antigen processing and presentation of peptide an
IFN-γ	GOTERM_BP_DIRECT	GO:0002479~antigen processing and presentation of exogenous
IFN-γ	GOTERM_BP_DIRECT	GO:0060071~Wnt signaling pathway, planar cell polarity pathway
IFN-γ	GOTERM_BP_DIRECT	GO:0006521~regulation of cellular amino acid metabolic process
IFN-γ	GOTERM_BP_DIRECT	GO:0051436~negative regulation of ubiquitin-protein ligase activ
IFN-γ	GOTERM_BP_DIRECT	GO:0051437~positive regulation of ubiquitin-protein ligase activit
IFN-γ	GOTERM_BP_DIRECT	GO:0031145~anaphase-promoting complex-dependent catabol
IFN-γ	GOTERM_BP_DIRECT	GO:0038095~Fc-epsilon receptor signaling pathway
IFN-γ	GOTERM_BP_DIRECT	GO:0043161~proteasome-mediated ubiquitin-dependent protei
IFN-γ	GOTERM_BP_DIRECT	GO:0038061~NIK/NF-kappaB signaling
IFN-γ	GOTERM_BP_DIRECT	GO:0043488~regulation of mRNA stability
IFN-γ	GOTERM_BP_DIRECT	GO:0002223~stimulatory C-type lectin receptor signaling pathwa
IFN-γ	GOTERM_BP_DIRECT	GO:0090263~positive regulation of canonical Wnt signaling pathv
IFN-γ	GOTERM_BP_DIRECT	GO:0090090~negative regulation of canonical Wnt signaling path
IFN-γ	GOTERM_BP_DIRECT	GO:0035456~response to interferon-beta
IFN-γ	GOTERM_BP_DIRECT	GO:0019885~antigen processing and presentation of endogenou
IFN-γ	GOTERM_BP_DIRECT	GO:0032728~positive regulation of interferon-beta production
IFN-γ	GOTERM_BP_DIRECT	GO:0045787~positive regulation of cell cycle
IFN-γ	GOTERM_BP_DIRECT	GO:0033209~tumor necrosis factor-mediated signaling pathway
IFN-γ	GOTERM_BP_DIRECT	GO:0032897~negative regulation of viral transcription
IFN-γ	GOTERM_BP_DIRECT	GO:0007259~JAK-STAT cascade
IFN-γ	GOTERM_BP_DIRECT	GO:0016925~protein sumoylation
IFN-γ	GOTERM_BP_DIRECT	GO:0032727~positive regulation of interferon-alpha production
IFN-γ	GOTERM_BP_DIRECT	GO:0035455~response to interferon-alpha
IFN-γ	GOTERM_BP_DIRECT	GO:0002481~antigen processing and presentation of exogenous

Count	%	PValue
6	3.529412	1.75E-03
6	3.529412	2.78E-03
4	2.352941	1.21E-02
13	7.647059	1.55E-02
8	4.705882	1.85E-02
4	2.352941	1.98E-02
9	5.294118	3.23E-02
8	4.705882	3.48E-02
5	2.941176	0.041048317
4	2.352941	0.041538026
7	4.117647	0.042607602
6	3.529412	0.043659292
3	1.764706	0.043918657
7	2.348993	0.012294309
5	1.677852	0.035739089
4	1.342282	0.04944385
6	2.013423	0.049475398
6	2.013423	0.049475398
6	2.013423	0.049475398
21	15.44118	1.33E-08
6	4.411765	5.29E-04
14	10.29412	0.001290604
4	2.941176	0.001302494
8	5.882353	0.001562825
8	5.882353	0.001987768
5	3.676471	0.002254758
5	3.676471	0.002254758
5	3.676471	0.003554191
10	7.352941	0.00445473
6	4.411765	0.005012779
6	4.411765	0.005012779
5	3.676471	0.005283005
4	2.941176	0.005859092
7	5.147059	0.006357053
4	2.941176	0.009728081
3	2.205882	0.014134378
3	2.205882	0.014134378
3	2.205882	0.026966262
3	2.205882	0.026966262
3	2.205882	0.026966262
3	2.205882	0.026966262
6	4.411765	0.031930606
6	4.411765	0.031930606
5	3.676471	0.03331084
4	2.941176	0.047139987
4	2.941176	0.047139987
14	7.45E+00	7.26E-09
26	1.38E+01	8.01E-09
15	7.98E+00	6.34E-07
8	4.255319	9.57E-05
28	14.89362	3.33E-04

12	6.382979	6.60E-04
10	5.319149	0.001113419
11	5.851064	0.002073798
5	2.659574	0.002739261
4	2.12766	0.00373327
4	2.12766	0.00373327
6	3.191489	0.006459259
6	3.191489	0.00923986
8	4.255319	0.009359768
9	4.787234	0.011472811
6	3.191489	0.012748251
4	2.12766	0.016009432
14	7.446809	0.017336794
5	2.659574	0.018675649
6	3.191489	0.022239883
4	2.12766	0.02596206
4	2.12766	0.02596206
3	1.595745	0.028303063
6	3.191489	0.028343863
6	3.191489	0.028343863
17	9.042553	0.041015159
9	4.787234	0.041706678
5	2.659574	0.044562978
106	2.70E+01	1.14E-19
29	7.38E+00	8.35E-11
73	1.86E+01	1.64E-09
13	3.31E+00	3.74E-08
56	1.42E+01	5.47E-08
41	1.04E+01	7.93E-08
31	7.89E+00	2.92E-07
23	5.85E+00	1.32E-06
32	8.14E+00	1.45E-06
21	5.34E+00	3.53E-06
16	4.07E+00	5.56E-06
18	4.58E+00	8.46E-06
24	6.11E+00	1.47E-05
13	3.31E+00	1.99E-05
36	9.16E+00	2.24E-05
11	2.80E+00	4.22E-05
19	4.83E+00	7.22E-05
22	5.60E+00	8.75E-05
11	2.80E+00	1.04E-04
21	5.34E+00	2.05E-04
44	1.12E+01	2.20E-04
19	4.83E+00	2.55E-04
27	6.870229	3.95E-04
18	4.580153	6.01E-04
8	2.035623	8.29E-04
12	3.053435	8.95E-04
21	5.343511	0.001047053
16	4.071247	0.001087439
7	1.78117	0.001193164

15	3.816794	0.001206228
10	2.544529	0.001393715
16	4.071247	0.001562299
18	4.580153	0.001653499
27	6.870229	0.002126014
9	2.290076	0.002290171
16	4.071247	0.003037615
71	18.06616	0.003255602
12	3.053435	0.003541528
8	2.035623	0.003767562
6	1.526718	0.004576108
6	1.526718	0.004576108
6	1.526718	0.004576108
12	3.053435	0.005213648
7	1.78117	0.006203833
11	2.798982	0.008494441
28	7.124682	0.009657874
14	3.562341	0.010136151
6	1.526718	0.010214348
12	3.053435	0.010376456
8	2.035623	0.011268572
28	7.124682	0.01135083
12	3.053435	0.014106334
5	1.272265	0.016753885
5	1.272265	0.016753885
5	1.272265	0.016753885
22	5.597964	0.016775005
8	2.035623	0.01757296
7	1.78117	0.018989642
6	1.526718	0.019258364
6	1.526718	0.019258364
6	1.526718	0.019258364
6	1.526718	0.019258364
9	2.290076	0.022573051
4	1.017812	0.027006568
4	1.017812	0.027006568
4	1.017812	0.027006568
4	1.017812	0.027006568
4	1.017812	0.027006568
4	1.017812	0.027006568
4	1.017812	0.027006568
4	1.017812	0.027006568
28	7.124682	0.027200124
7	1.78117	0.029455589
6	1.526718	0.032312894
5	1.272265	0.03299092
5	1.272265	0.03299092
5	1.272265	0.03299092
5	1.272265	0.03299092
5	1.272265	0.03299092
17	4.3257	0.034484222
7	1.78117	0.043095852

7	1.78117	0.043095852
6	1.526718	0.049758138
6	1.526718	0.049758138
14	3.562341	0.049853567
22	9.606987	3.33E-06
15	6.550218	1.75E-04
13	5.676856	2.64E-04
11	4.803493	7.39E-04
5	2.183406	9.62E-04
8	3.49345	9.69E-04
11	4.803493	0.001029006
18	7.860262	0.001052843
12	5.240175	0.001119538
10	4.366812	0.001237341
10	4.366812	0.001237341
10	4.366812	0.001237341
10	4.366812	0.001237341
11	4.803493	0.00140613
15	6.550218	0.002549049
5	2.183406	0.002610187
8	3.49345	0.004940155
12	5.240175	0.004982465
9	3.930131	0.005410311
5	2.183406	0.005512713
4	1.746725	0.00644957
17	7.423581	0.006563635
12	5.240175	0.007528906
6	2.620087	0.009752696
5	2.183406	0.009983975
5	2.183406	0.009983975
7	3.056769	0.011670209
10	4.366812	0.013654773
6	2.620087	0.014308067
4	1.746725	0.014681443
4	1.746725	0.014681443
20	8.733624	0.01479699
5	2.183406	0.01628096
6	2.620087	0.020101271
7	3.056769	0.0207669
9	3.930131	0.022571332
15	6.550218	0.023336868
5	2.183406	0.024594256
5	2.183406	0.024594256
5	2.183406	0.024594256
4	1.746725	0.026752698
4	1.746725	0.026752698
12	5.240175	0.028798396
10	4.366812	0.033608069
5	2.183406	0.035046058
5	2.183406	0.035046058
3	1.310044	0.040563981
3	1.310044	0.040563981

3	1.310044	0.040563981
3	1.310044	0.040563981
3	1.310044	0.040563981
3	1.310044	0.040563981
4	1.746725	0.042682687
4	1.746725	0.042682687
4	1.746725	0.042682687
6	2.620087	0.045871753
6	2.620087	0.045871753
5	2.183406	0.04769274
5	2.183406	0.04769274
21	14.09396	2.96E-13
19	12.75168	4.80E-09
21	14.09396	1.60E-08
17	11.4094	9.19E-08
10	6.711409	5.47E-07
12	8.053691	1.75E-05
9	6.040268	1.31E-04
12	8.053691	1.66E-04
10	6.711409	2.00E-04
9	6.040268	2.64E-04
9	6.040268	2.64E-04
9	6.040268	2.64E-04
9	6.040268	2.64E-04
11	7.38255	3.96E-04
10	6.711409	5.88E-04
9	6.040268	6.53E-04
9	6.040268	8.56E-04
10	6.711409	0.002161284
9	6.040268	0.002742408
10	6.711409	0.00312608
4	2.684564	0.007592174
4	2.684564	0.012540051
4	2.684564	0.012540051
4	2.684564	0.012540051
12	8.053691	0.014483579
3	2.013423	0.016886858
5	3.355705	0.018547866
3	2.013423	0.032068049
3	2.013423	0.032068049
3	2.013423	0.032068049
3	2.013423	0.032068049

Genes
5795, 5800, 26191, 5778, 5788, 1846
57460, 5800, 26191, 132160, 5778, 5788
1436, 1435, 4318, 6688
958, 3604, 8764, 6678, 1439, 8797, 8771, 6374, 8792, 26191, 8794, 4791, 54
958, 408, 2769, 2207, 3690, 27040, 5294, 2153
1436, 1435, 8792, 8600
8797, 7040, 8792, 8794, 838, 3604, 655, 8764, 1508
81794, 3687, 7305, 27299, 2207, 3690, 27040, 101
920, 1435, 27040, 5788, 9770
90993, 7040, 10911, 2022
1436, 958, 408, 7040, 4318, 3690, 2022
8792, 4318, 655, 6678, 9770, 8600
920, 7040, 655
3779, 6753, 3751, 9495, 8973, 1621, 6539
116, 3593, 3751, 59344, 1269
9424, 3751, 3757, 9254
162514, 123041, 9254, 116443, 489, 255231
5493, 1001, 84518, 3713, 2810, 2125
9424, 3751, 23704, 3766, 9254, 6332
79812, 1306, 91010, 94, 1284, 54567, 2702, 54538, 7010, 22846, 2321, 3791, 2335, 8829, 7075, 4223, 80781, 51[illegible]
4855, 1282, 8829, 54567, 7048, 23129
9719, 1282, 3672, 5155, 1284, 50509, 633, 3384, 7450, 3791, 2335, 3680, 3910, 80781
2701, 2702, 3791, 947
94, 5155, 26051, 8829, 7010, 3791, 2324, 6369
5155, 2149, 7293, 2321, 3791, 3479, 2324, 8718
94, 54567, 22846, 9353, 5797
8829, 54567, 2321, 3791, 2324
2702, 26508, 7048, 23129, 64321
94, 2624, 7010, 358, 6915, 2321, 3791, 7048, 6369, 947
135, 94, 54567, 4223, 552, 2313
5155, 8829, 2149, 22846, 7450, 2335
8322, 7075, 3791, 7048, 64321
1910, 135, 3764, 3672
51738, 1284, 7075, 7010, 22846, 2828, 5797
5155, 8829, 50507, 3479
1910, 135, 552
64805, 7450, 2313
1282, 2321, 2324
8829, 9353, 5797
22899, 8322, 8829
79812, 54567, 4223
1003, 90952, 4345, 5797, 51294, 27253
3764, 5155, 2624, 7010, 7048, 2057
9079, 54538, 3910, 7852, 23129
2702, 2149, 6915, 552
94, 54567, 1636, 633
1281, 1301, 1303, 26585, 6423, 140766, 1805, 4060, 2331, 1277, 1289, 7373, 1278, 4017
1292, 10516, 1293, 1281, 1291, 22795, 1295, 11117, 1301, 1300, 5054, 7412, 1842, 25903, 10631, 4060, 3678, 1[illegible]
1292, 1293, 1281, 4313, 1291, 4312, 1295, 1301, 1300, 1303, 140766, 1277, 1289, 1278, 4323
4313, 1291, 3678, 1295, 1301, 1303, 4323, 3624
1292, 1293, 1291, 22795, 1295, 145864, 1805, 10631, 3678, 1289, 22801, 4017, 7045, 2191, 10154, 3671, 7130, [illegible]

3356, 7412, 1281, 5176, 7077, 1490, 1634, 3861, 4017, 3868, 4914, 7043
1281, 10631, 1277, 1300, 1278, 1303, 145864, 165, 9507, 2200
1909, 6387, 7412, 4313, 10631, 6649, 7424, 857, 4017, 4323, 7043
131578, 1634, 127435, 857, 54829
1292, 1291, 1277, 1278
1281, 4239, 4053, 1289
1281, 4313, 1291, 1956, 1277, 1278
6387, 1281, 10631, 1634, 624, 4323
4313, 4312, 7177, 7077, 1634, 4323, 9507, 2200
10516, 7412, 1281, 1842, 25992, 22795, 1490, 22801, 81792
5737, 10631, 1277, 1490, 2706, 5159
83729, 8200, 7043, 3624
4313, 1295, 857, 5054, 51330, 57125, 7070, 7424, 3678, 1490, 2028, 7045, 4323, 2191
7431, 83729, 8200, 7043, 3624
1601, 27123, 51339, 3090, 7472, 6423
4313, 1277, 83716, 7043
27123, 51339, 3090, 6423
10848, 3861, 3868
10631, 1277, 5139, 857, 7472, 2200
4060, 1277, 83872, 1301, 7078, 7045
5737, 7292, 1956, 1489, 3082, 2252, 7472, 6423, 26585, 23529, 3356, 150, 57124, 9180, 7424, 1490, 5159
3671, 5919, 7424, 3082, 2162, 7078, 87, 5054, 7043
131578, 7070, 1634, 127435, 54829
7535, 10344, 942, 8741, 8742, 6347, 943, 6348, 944, 6352, 6351, 6357, 6355, 2209, 1521, 29851, 1520, 4689, [illegible]
3127, 941, 942, 940, 10673, 84433, 921, 29851, 151888, 5777, 5133, 959, 3113, 3115, 3117, 3118, 3119, 7409, [illegible]
7535, 10344, 3627, 2919, 7124, 6347, 943, 6348, 3683, 9450, 6352, 6351, 3965, 6357, 199, 6355, 414062, 84868, [illegible]
3113, 3127, 3111, 3115, 3112, 3117, 3118, 3119, 3109, 3108, 3120, 3123, 3122
10344, 3627, 151306, 84636, 2919, 6347, 6348, 6352, 6351, 1524, 6357, 2857, 6355, 414062, 5777, 7409, 5032, [illegible]
10859, 3820, 56253, 10870, 146722, 3903, 27180, 925, 3904, 84433, 3805, 131450, 3683, 342510, 2209, 3106, [illegible]
10344, 3627, 2919, 6347, 6348, 6846, 10563, 6352, 6351, 6376, 1524, 6375, 6357, 6373, 6355, 6372, 729230, [illegible]
3113, 959, 3115, 51561, 11151, 5588, 940, 10673, 916, 84433, 3600, 3718, 912, 6352, 6693, 3458, 6363, 199, [illegible]
10344, 3627, 2357, 2919, 6347, 6348, 6352, 3965, 728, 6376, 1524, 6357, 6373, 6355, 6372, 729230, 2833, 2920, [illegible]
3594, 3113, 10344, 6347, 6348, 6846, 6362, 115362, 6352, 3965, 6351, 6376, 6363, 6375, 6366, 6357, 199, 4261, [illegible]
3113, 3127, 3111, 3115, 3112, 3117, 3118, 3119, 972, 5641, 1520, 3109, 3108, 3120, 3123, 3122
4049, 2625, 931, 684, 940, 7124, 6347, 9450, 3458, 54209, 6480, 10148, 3569, 5452, 4068, 5699, 54210, 5133
10344, 6279, 1441, 7409, 6347, 2921, 6348, 6846, 6280, 6362, 6352, 6351, 3458, 728, 6376, 6375, 6357, 3689, [illegible]
10859, 3127, 3135, 6693, 10288, 84868, 3586, 3559, 5777, 3123, 1493, 11326, 50943
10344, 4049, 3627, 941, 942, 684, 3606, 6348, 944, 6280, 3600, 6352, 10563, 6351, 6375, 6357, 4050, 6355, [illegible]
719, 728, 6363, 51561, 6375, 6366, 1236, 2919, 2920, 2921, 972
3113, 3127, 3115, 3117, 3135, 3118, 3119, 925, 972, 3458, 1520, 3106, 3109, 2217, 3133, 3105, 30835, 3123, [illegible]
10859, 3627, 941, 942, 7124, 6347, 2672, 57817, 7133, 3394, 929, 1234, 3458, 1524, 7099, 10288, 84868, 4064, [illegible]
6352, 929, 7099, 3606, 7124, 6347, 3553, 23643, 6348, 5724, 3055
3662, 3113, 3127, 3115, 3117, 3135, 3118, 3119, 3394, 5724, 3458, 4261, 2209, 3106, 3134, 3133, 3120, 3105, [illegible]
7535, 64170, 2625, 11151, 684, 2268, 3004, 6280, 342510, 9450, 79626, 3106, 84868, 340205, 3133, 54210, [illegible]
3627, 2357, 5588, 2919, 2920, 6347, 6348, 409, 4283, 6352, 719, 728, 6376, 6363, 6366, 6357, 6355, 6372, [illegible]
7535, 3127, 2625, 940, 84433, 7454, 3458, 5699, 50943, 3113, 3115, 3117, 5588, 3118, 3119, 5705, 915, 5720, [illegible]
4332, 3135, 4688, 23643, 3805, 5551, 8302, 4283, 1234, 6693, 728, 10578, 1524, 23601, 653361, 10288, 729230, [illegible]
3662, 941, 5588, 942, 940, 916, 3553, 84433
929, 64170, 3458, 6363, 51561, 7099, 914, 6347, 23643, 6348, 3133, 5724
10344, 51561, 6279, 3606, 6347, 6348, 6846, 3600, 6280, 6362, 6352, 6351, 6376, 6375, 6357, 7099, 6368, 6355, [illegible]
10344, 6347, 6348, 6846, 6362, 6352, 6351, 6363, 6376, 6375, 6366, 6357, 6368, 6355, 414062, 3569
7535, 6693, 1236, 940, 916, 972, 1794

3113, 3594, 3115, 4049, 51561, 3606, 7124, 916, 3394, 929, 7099, 729230, 84868, 3553, 3105
10859, 3127, 3965, 6375, 2625, 7099, 84868, 3586, 3123, 50943
5790, 2625, 1441, 10990, 962, 5032, 9235, 7454, 972, 11025, 4283, 11024, 6376, 11027, 3106, 11262
10344, 2625, 6347, 57817, 6348, 6846, 6362, 6352, 6351, 6376, 6363, 6375, 6366, 6357, 6368, 6355, 414062, 35[illegible]
10344, 7124, 6347, 9290, 6348, 6846, 972, 6504, 6362, 409, 6352, 6351, 728, 3965, 6363, 6376, 6357, 1236, 637[illegible]
959, 3965, 2625, 5588, 940, 84868, 916, 3133, 50943
10344, 6347, 6348, 6846, 6362, 6352, 6351, 6363, 6376, 6375, 6366, 6357, 6368, 6355, 414062, 3569
10859, 3111, 10344, 3627, 3112, 3581, 4354, 2919, 8741, 8742, 6347, 943, 944, 84433, 3683, 9046, 6351, 6357, [illegible]
3662, 7535, 941, 942, 914, 916, 962, 84174, 925, 7454, 924, 50943
409, 3965, 7099, 84868, 3586, 2841, 6504, 50943
6693, 7099, 729230, 7096, 943, 1536
7535, 941, 940, 10148, 729230, 916
5551, 6363, 6366, 11151, 81704, 1794
6362, 10344, 6376, 6363, 6357, 6366, 6368, 6355, 414062, 6347, 6348, 6846
4283, 3627, 6375, 6373, 5473, 6372, 3569
64170, 4049, 728, 7124, 84868, 944, 2268, 3133, 3569, 3105, 4069
4049, 8742, 3004, 944, 6280, 83593, 9450, 3458, 728, 3553, 5777, 3559, 7185, 2833, 2999, 5133, 11040, 6279, 91[illegible]
6279, 57817, 3394, 6280, 23166, 10563, 3458, 6693, 10578, 7099, 6372, 5473, 7096, 3586
929, 3965, 7099, 3689, 23643, 3684
929, 6693, 7099, 356, 940, 407977, 8742, 916, 23643, 943, 7133, 921
3965, 6363, 51561, 7099, 3606, 7124, 51284, 3553
7535, 10859, 56253, 942, 3903, 10990, 924, 8320, 3458, 1520, 84868, 151888, 3133, 10462, 51744, 973, 23495, [illegible]
409, 23533, 6363, 3059, 6366, 2625, 1236, 3606, 940, 7124, 6348, 3569
6375, 2625, 84868, 11326, 50943
6352, 6376, 1524, 6347, 6348
10563, 374403, 6366, 1236, 8477
3627, 4049, 4129, 6279, 2919, 2920, 23643, 2921, 943, 8784, 6403, 10563, 4283, 3965, 728, 1236, 7099, 356, 63[illegible]
959, 3458, 6363, 51561, 3135, 1236, 7099, 3394
409, 7099, 7124, 84868, 3586, 6504, 50943
3113, 3115, 5588, 729230, 6347, 3932
6376, 6279, 3689, 5473, 6347, 6280
717, 718, 713, 712, 2219, 714
5551, 3002, 3001, 3004, 2999, 4069
1089, 929, 3689, 11151, 122618, 201294, 3683, 3394, 6504
3111, 914, 84433, 3600
7535, 915, 972, 1794
929, 7099, 356, 23643
3458, 7096, 3553, 5724
4283, 3627, 6373, 2833
7099, 7124, 51284, 51311
6363, 6366, 1236, 1794
3109, 3108, 3123, 3122
3820, 3627, 940, 10990, 3902, 6347, 27180, 925, 10563, 9046, 3458, 10261, 3559, 2833, 951, 914, 915, 23643, 91[illegible]
7099, 5641, 1520, 51284, 7096, 4064, 51311
929, 56253, 23601, 7124, 2268, 3586
11314, 4332, 3586, 23495, 1493
3627, 6373, 58191, 6348, 2833
3458, 7124, 3553, 7133, 348
7099, 7124, 84868, 51284, 3569
3127, 7099, 3606, 10148, 3123
2357, 914, 51744, 962, 3683, 3684, 972, 6403, 9046, 719, 728, 6693, 9056, 3689, 5777, 3932, 54210
3662, 3458, 10538, 3586, 3394, 3569, 10077

929, 64170, 7099, 497189, 7124, 23643, 51311
929, 7099, 51284, 7096, 23643, 51311
3594, 51561, 6375, 3106, 3133, 3105
3135, 4689, 4688, 5705, 5720, 5721, 653361, 2209, 3106, 3134, 3133, 1536, 3105, 5699
657, 22853, 26524, 91, 23043, 90, 9262, 55589, 22858, 1385, 3717, 7042, 9113, 904, 57448, 2534, 7046, 10114, 1[illegible]
5717, 80114, 4040, 5708, 9113, 26524, 5706, 10197, 2487, 10213, 5711, 9861, 5700, 5701, 5716
5717, 5708, 5706, 10197, 5887, 10213, 5711, 9575, 9861, 5700, 5701, 55175, 5716
5717, 5708, 5706, 10197, 10213, 5711, 9861, 5700, 5701, 5716, 5966
3570, 1270, 3572, 1271, 3977
219333, 5717, 7494, 158880, 26133, 5706, 10213, 5701
5717, 5708, 5706, 10197, 10213, 5711, 3312, 9861, 5700, 5701, 5716
23228, 5334, 26524, 5577, 23043, 5332, 323, 64759, 3815, 9262, 22858, 3717, 2977, 5576, 2534, 2982, 4649, 70[illegible]
5717, 85458, 4040, 5708, 5706, 10197, 10213, 5711, 9861, 5700, 5701, 5716
5717, 5708, 5706, 10197, 10213, 5711, 9861, 5700, 5701, 5716
5717, 5708, 5706, 10197, 10213, 5711, 9861, 5700, 5701, 5716
5717, 5708, 5706, 10197, 10213, 5711, 9861, 5700, 5701, 5716
5717, 5708, 5706, 10197, 10213, 5711, 9861, 5700, 5701, 5716
5717, 5708, 10082, 5706, 10197, 10213, 5711, 9861, 5700, 5701, 5716
659, 4208, 7494, 6297, 2033, 9575, 1385, 5978, 4802, 4800, 2957, 904, 2113, 80714, 5966
22853, 91, 90, 7046, 10114
659, 4208, 657, 3570, 3572, 90, 338773, 4921
5717, 5708, 5706, 2033, 10197, 2534, 10213, 5711, 9861, 5700, 5701, 5716
7253, 4208, 55816, 43, 1271, 2033, 91851, 54510, 7903
659, 7042, 657, 90, 23414
8642, 9113, 26524, 79633
5717, 4208, 5708, 5156, 5706, 10213, 3815, 5711, 9861, 5700, 5701, 5716, 3717, 55816, 4254, 10197, 2534
5717, 5708, 5706, 50489, 10197, 10213, 5711, 9861, 5700, 5701, 5716, 948
3306, 3320, 3312, 3326, 3308, 7057
3688, 9113, 26524, 91, 90
659, 7042, 90, 23414, 7046
659, 7042, 657, 91, 90, 7046, 57551
5717, 5708, 5706, 10197, 10213, 5711, 9861, 5700, 5701, 5716
146057, 22853, 26524, 7046, 127933, 10114
146057, 22858, 51762, 79809
8646, 2534, 5592, 10395
4208, 7586, 9392, 2033, 91, 323, 65986, 9575, 1385, 55206, 10499, 5978, 55252, 158880, 4802, 4800, 4801, 295[illegible]
9113, 26524, 904, 57448, 3980
4802, 3320, 51661, 3312, 3326, 821
657, 3688, 91, 90, 9871, 23414, 7046
22853, 5156, 91, 23043, 9262, 3815, 127933, 4921, 3717
4208, 4040, 90, 5994, 9575, 1385, 5978, 1389, 4800, 3454, 4801, 2113, 7046, 10746, 80714
10197, 90, 57448, 7046, 7057
7042, 85458, 10395, 93663, 9475
659, 445815, 91, 90, 11217
659, 8646, 657, 90
7253, 9113, 26524, 2690
10550, 5978, 3688, 8633, 9113, 26524, 2487, 323, 133746, 1385, 1545, 10687
5717, 5708, 5706, 10197, 10213, 5711, 9861, 5700, 5701, 5716
55252, 7494, 1389, 2487, 1385
3688, 2033, 22858, 1875, 3312
4208, 4040, 2487
659, 7042, 657

657, 1832, 7046
3320, 9113, 3312
657, 5887, 23414
659, 657, 90
5706, 1385, 5700, 5701
3320, 2033, 3312, 3326
7042, 4208, 7494, 1270
85458, 6801, 4040, 23043, 2487, 8840
659, 4208, 657, 6546, 90, 338773
7042, 4208, 657, 4040, 7046
287, 4040, 8642, 23043, 51735
3430, 10410, 8519, 3136, 6772, 4599, 8638, 5696, 4938, 4939, 3433, 3429, 3437, 6672, 3455, 3107, 3659, 3669, [illegible]
10346, 64135, 10410, 8519, 3592, 4599, 8638, 3460, 4938, 4939, 10561, 3433, 3437, 3455, 3669, 23586, 3665, [illegible]
10346, 10410, 8519, 2633, 6772, 4599, 8638, 5371, 10964, 60489, 4938, 4939, 3433, 3437, 3455, 3659, 200315, [illegible]
10346, 3136, 2633, 6772, 6737, 8638, 5371, 3460, 4938, 4939, 6672, 9111, 3107, 3659, 567, 3665, 4940
4938, 10410, 8519, 4599, 200315, 3669, 8638, 60489, 91543, 4940
5719, 26270, 55008, 5709, 6737, 5704, 5710, 5702, 5713, 5714, 5698, 5696
811, 3136, 3107, 6891, 6892, 567, 3077, 6890, 3140
5719, 3136, 5709, 3107, 5704, 5710, 5702, 567, 5713, 5714, 5698, 5696
5719, 115908, 5709, 5704, 5710, 5702, 5713, 5714, 5698, 5696
5719, 5709, 5704, 5710, 5702, 5713, 5714, 5698, 5696
5719, 5709, 5704, 5710, 5702, 5713, 5714, 5698, 5696
5719, 5709, 5704, 5710, 5702, 5713, 5714, 5698, 5696
5719, 5709, 5704, 5710, 5702, 5713, 5714, 5698, 5696
5719, 5709, 5704, 5710, 5702, 2205, 5713, 5714, 4067, 5698, 5696
5719, 5709, 5704, 5710, 5702, 5371, 5713, 5714, 5698, 5696
5719, 5709, 5704, 5710, 5702, 5713, 5714, 5698, 5696
5719, 5709, 5704, 5710, 5702, 5713, 5714, 5698, 5696
5719, 5709, 5704, 5710, 5702, 5713, 5714, 4067, 5698, 5696
5719, 5709, 5704, 5710, 5702, 5713, 5714, 5698, 5696
5719, 115908, 5709, 5704, 5710, 5702, 5713, 5714, 5698, 5696
10410, 8519, 6772, 3455
6891, 6892, 567, 6890
64135, 3659, 23586, 3665
811, 7051, 6737, 2323
5719, 5709, 6772, 5704, 5710, 5702, 5713, 5714, 4982, 8995, 5698, 5696
10410, 6672, 6737
6772, 9111, 3455, 6776, 3601
64135, 6672, 5371
64135, 23586, 3665
10410, 8519, 3455
6891, 567, 6890

List Total	Pop Hits	Pop Total	Fold Enrich	Bonferroni	Benjamini	FDR
164	11	1786	5.940133	8.78E-01	8.78E-01	2.79E+00
164	12	1786	5.445122	9.65E-01	8.12E-01	4.40E+00
164	6	1786	7.260163	1.00E+00	9.92E-01	1.79E+01
164	67	1786	2.113032	1.00E+00	9.91E-01	2.23E+01
164	31	1786	2.810386	1	9.89E-01	2.61E+01
164	7	1786	6.222997	1	9.82E-01	27.67188
164	42	1786	2.333624	1	0.996451504	41.23431
164	35	1786	2.489199	1	0.995091783	43.59545
164	15	1786	3.630081	1	0.996277161	49.22114
164	9	1786	4.840108	1	0.993874466	49.63879
164	29	1786	2.62868	1	0.991382743	50.53979
164	22	1786	2.970067	1	0.988527749	51.41096
164	4	1786	8.167683	1	0.98422303	51.62359
274	14	1786	3.259124	0.999999934	0.999999934	18.34604
274	9	1786	3.621249	1	1	44.91391
274	6	1786	4.345499	1	1	56.43022
274	14	1786	2.793535	1	0.999999957	56.4539
274	14	1786	2.793535	1	0.999999957	56.4539
274	14	1786	2.793535	1	0.999999957	56.4539
127	69	1786	4.280041	1.51E-05	1.51E-05	2.13E-05
127	11	1786	7.670723	0.453095607	0.260470154	0.8461
127	73	1786	2.697012	0.770884849	0.388094153	2.053352
127	4	1786	14.06299	0.773976081	0.310493126	2.072083
127	26	1786	4.327075	0.83213221	0.300168519	2.481375
127	27	1786	4.166812	0.896718883	0.315032203	3.146025
127	9	1786	7.812773	0.923890608	0.307841646	3.561446
127	9	1786	7.812773	0.923890608	0.307841646	3.561446
127	10	1786	7.031496	0.982795007	0.398193584	5.559544
127	47	1786	2.992126	0.993867802	0.432220678	6.921428
127	17	1786	4.963409	0.996765771	0.436392087	7.756099
127	17	1786	4.963409	0.996765771	0.436392087	7.756099
127	11	1786	6.392269	0.997627683	0.422729527	8.157748
127	6	1786	9.375328	0.998775086	0.428070994	9.008542
127	25	1786	3.937638	0.999308433	0.428639352	9.737995
127	7	1786	8.035996	0.999985683	0.549194896	14.53379
127	3	1786	14.06299	0.999999912	0.661362716	20.44267
127	3	1786	14.06299	0.999999912	0.661362716	20.44267
127	4	1786	10.54724	1	0.857645633	35.54274
127	4	1786	10.54724	1	0.857645633	35.54274
127	4	1786	10.54724	1	0.857645633	35.54274
127	4	1786	10.54724	1	0.857645633	35.54274
127	26	1786	3.245306	1	0.886739944	40.62763
127	26	1786	3.245306	1	0.886739944	40.62763
127	18	1786	3.906387	1	0.883225054	41.97306
127	12	1786	4.687664	1	0.944964568	53.96401
127	12	1786	4.687664	1	0.944964568	53.96401
181	21	1786	6.578269	8.90E-06	8.90E-06	1.18E-05
181	73	1786	3.514418	9.82E-06	4.91E-06	1.30E-05
181	32	1786	4.625345	7.77E-04	2.59E-04	0.001028
181	13	1786	6.072248	0.110724801	0.028910977	0.155034
181	138	1786	2.002082	0.335177484	0.078402806	0.538302

181	37	1786	3.200239	0.554726477	0.126148146	1.064022
181	28	1786	3.524073	0.744826773	0.177260861	1.789629
181	36	1786	3.01504	0.921536084	0.272498744	3.309087
181	7	1786	7.048145	0.965366778	0.31178928	4.348978
181	4	1786	9.867403	0.989801901	0.367803739	5.882747
181	4	1786	9.867403	0.989801901	0.367803739	5.882747
181	13	1786	4.554186	0.999645477	0.514341151	9.971412
181	14	1786	4.228887	0.999988587	0.612637375	13.96987
181	25	1786	3.157569	0.999990161	0.588050691	14.13849
181	32	1786	2.775207	0.999999282	0.635962436	17.05953
181	15	1786	3.946961	0.999999853	0.649591485	18.77728
181	6	1786	6.578269	0.999999997	0.709644236	23.01884
181	69	1786	2.002082	1	0.716701372	24.68498
181	11	1786	4.485183	1	0.723085661	26.33122
181	17	1786	3.482613	1	0.765724165	30.55071
181	7	1786	5.638516	1	0.800611062	34.71443
181	7	1786	5.638516	1	0.800611062	34.71443
181	3	1786	9.867403	1	0.812915334	37.21191
181	18	1786	3.289134	1	0.79857603	37.25463
181	18	1786	3.289134	1	0.79857603	37.25463
181	101	1786	1.66085	1	0.892727901	49.28085
181	40	1786	2.220166	1	0.88653034	49.87046
181	14	1786	3.524073	1	0.893067606	52.23834
358	242	1786	2.19E+00	2.06E-16	2.06E-16	1.94E-16
358	43	1786	3.36E+00	1.50E-07	7.52E-08	1.42E-07
358	192	1786	1.90E+00	2.95E-06	9.85E-07	2.79E-06
358	13	1786	4.988827	6.73E-05	1.68E-05	6.34E-05
358	142	1786	1.967425	9.85E-05	1.97E-05	9.29E-05
358	91	1786	2.247713	1.43E-04	2.38E-05	1.35E-04
358	62	1786	2.494413	5.25E-04	7.50E-05	4.95E-04
358	41	1786	2.79861	0.002372205	2.97E-04	0.00224
358	69	1786	2.313659	0.002610203	2.90E-04	0.002465
358	37	1786	2.831496	0.006336266	6.35E-04	0.005994
358	24	1786	3.325885	0.009963603	9.10E-04	0.009442
358	30	1786	2.993296	0.015127591	0.001269459	0.014373
358	49	1786	2.443507	0.026071657	0.002030056	0.024908
358	18	1786	3.603042	0.035189235	0.002555536	0.033774
358	91	1786	1.973602	0.039525854	0.002684937	0.038021
358	14	1786	3.919792	0.073147541	0.004736304	0.071603
358	37	1786	2.56183	0.121859845	0.007614921	0.122462
358	47	1786	2.335196	0.145733823	0.008712516	0.148418
358	15	1786	3.658473	0.171083405	0.009826962	0.176777
358	46	1786	2.277508	0.3093488	0.018335833	0.348402
358	131	1786	1.675636	0.32754065	0.018718478	0.373481
358	40	1786	2.369693	0.3677225	0.020621984	0.431347
358	69	1786	1.95215	0.509442063	0.030491207	0.669339
358	39	1786	2.302535	0.661149203	0.044089964	1.015281
358	10	1786	3.991061	0.775584251	0.058019021	1.399147
358	21	1786	2.850758	0.800660661	0.060144105	1.509255
358	51	1786	2.054223	0.84843335	0.067493244	1.763384
358	34	1786	2.347683	0.859077255	0.067590977	1.83081
358	8	1786	4.365223	0.883535835	0.071461886	2.007116

358	31	1786	2.413948	0.886247185	0.069894801	2.02888
358	16	1786	3.118017	0.918880454	0.077831039	2.340734
358	35	1786	2.280607	0.940149243	0.084236296	2.620348
358	42	1786	2.138069	0.94922835	0.086357271	2.771298
358	76	1786	1.772346	0.978355991	0.106613585	3.549863
358	14	1786	3.207103	0.983906317	0.111286895	3.81897
358	37	1786	2.157331	0.995826795	0.14118042	5.035364
358	264	1786	1.341692	0.997185316	0.146770882	5.387381
358	24	1786	2.494413	0.998321093	0.154771013	5.847247
358	12	1786	3.325885	0.998884211	0.15996578	6.209297
358	7	1786	4.276137	0.999741467	0.186583613	7.49369
358	7	1786	4.276137	0.999741467	0.186583613	7.49369
358	7	1786	4.276137	0.999741467	0.186583613	7.49369
358	25	1786	2.394637	0.999918454	0.205162926	8.494745
358	10	1786	3.492179	0.999986433	0.234215824	10.02933
358	23	1786	2.385961	0.999999787	0.30043635	13.48698
358	88	1786	1.587354	0.999999974	0.327824392	15.19491
358	34	1786	2.054223	0.999999989	0.334849042	15.88777
358	8	1786	3.74162	0.999999991	0.330999435	16.00055
358	27	1786	2.217256	0.999999993	0.329474994	16.23388
358	14	1786	2.850758	0.999999999	0.346365185	17.5071
358	89	1786	1.569519	0.999999999	0.342681615	17.62357
358	28	1786	2.138069	1	0.400541191	21.43708
358	6	1786	4.157356	1	0.449349132	24.94415
358	6	1786	4.157356	1	0.449349132	24.94415
358	6	1786	4.157356	1	0.449349132	24.94415
358	67	1786	1.638122	1	0.443408746	24.97153
358	15	1786	2.660708	1	0.452535794	25.99894
358	12	1786	2.910149	1	0.472407156	27.79046
358	9	1786	3.325885	1	0.471004512	28.12564
358	9	1786	3.325885	1	0.471004512	28.12564
358	9	1786	3.325885	1	0.471004512	28.12564
358	9	1786	3.325885	1	0.471004512	28.12564
358	19	1786	2.363128	1	0.52015134	32.14152
358	4	1786	4.988827	1	0.578968257	37.18355
358	4	1786	4.988827	1	0.578968257	37.18355
358	4	1786	4.988827	1	0.578968257	37.18355
358	4	1786	4.988827	1	0.578968257	37.18355
358	4	1786	4.988827	1	0.578968257	37.18355
358	4	1786	4.988827	1	0.578968257	37.18355
358	4	1786	4.988827	1	0.578968257	37.18355
358	4	1786	4.988827	1	0.578968257	37.18355
358	95	1786	1.470391	1	0.575273626	37.39543
358	13	1786	2.686291	1	0.598542127	39.81534
358	10	1786	2.993296	1	0.626912349	42.75467
358	7	1786	3.563448	1	0.628599048	43.43204
358	7	1786	3.563448	1	0.628599048	43.43204
358	7	1786	3.563448	1	0.628599048	43.43204
358	7	1786	3.563448	1	0.628599048	43.43204
358	7	1786	3.563448	1	0.628599048	43.43204
358	51	1786	1.662942	1	0.639185129	44.89741
358	14	1786	2.494413	1	0.716155655	52.67472

358	14	1786	2.494413	1	0.716155655	52.67472
358	11	1786	2.721178	1	0.762184925	57.96968
358	11	1786	2.721178	1	0.762184925	57.96968
358	41	1786	1.703502	1	0.757547216	58.0413
218	61	1786	2.95473	0.005311335	0.005311335	0.00557
218	41	1786	2.997315	0.2442855	0.130681589	0.292544
218	33	1786	3.227412	0.344226517	0.131203724	0.440373
218	27	1786	3.337751	0.693791641	0.25611764	1.230288
218	5	1786	8.192661	0.785694099	0.265136248	1.598282
218	15	1786	4.369419	0.788307964	0.227999787	1.610912
218	28	1786	3.218545	0.807620638	0.209800181	1.709314
218	64	1786	2.304186	0.814831634	0.190073052	1.748584
218	33	1786	2.979149	0.833603929	0.1806672	1.858378
218	24	1786	3.413609	0.862235526	0.179811409	2.052026
218	24	1786	3.413609	0.862235526	0.179811409	2.052026
218	24	1786	3.413609	0.862235526	0.179811409	2.052026
218	24	1786	3.413609	0.862235526	0.179811409	2.052026
218	29	1786	3.107561	0.894895319	0.185189624	2.328859
218	52	1786	2.363267	0.983197681	0.288599604	4.184111
218	6	1786	6.827217	0.984768236	0.275211065	4.282413
218	19	1786	3.449541	0.999639778	0.432402134	7.958846
218	39	1786	2.520819	0.999663484	0.413231183	8.024362
218	24	1786	3.072248	0.999830968	0.418903841	8.684415
218	7	1786	5.8519	0.999856657	0.405838543	8.841733
218	4	1786	8.192661	0.9999683	0.437582791	10.26922
218	69	1786	2.018482	0.999973623	0.425867837	10.44157
218	41	1786	2.397852	0.999994437	0.453908755	11.88773
218	12	1786	4.09633	0.999999847	0.52629778	15.13626
218	8	1786	5.120413	0.999999895	0.518193355	15.46757
218	8	1786	5.120413	0.999999895	0.518193355	15.46757
218	17	1786	3.373448	0.999999993	0.558300434	17.84662
218	33	1786	2.482624	1	0.600348882	20.56591
218	13	1786	3.781228	1	0.602637771	21.44239
218	5	1786	6.554128	1	0.597773545	21.93923
218	5	1786	6.554128	1	0.597773545	21.93923
218	94	1786	1.743119	1	0.5868576	22.09239
218	9	1786	4.551478	1	0.608818428	24.03442
218	14	1786	3.51114	1	0.67405742	28.82662
218	19	1786	3.018349	1	0.673697244	29.63194
218	30	1786	2.457798	1	0.692431407	31.77217
218	66	1786	1.861968	1	0.693154889	32.66154
218	10	1786	4.09633	1	0.701239192	34.09872
218	10	1786	4.09633	1	0.701239192	34.09872
218	10	1786	4.09633	1	0.701239192	34.09872
218	6	1786	5.461774	1	0.72109798	36.49876
218	6	1786	5.461774	1	0.72109798	36.49876
218	49	1786	2.006366	1	0.737278701	38.69736
218	38	1786	2.155963	1	0.781357775	43.58787
218	11	1786	3.723937	1	0.786402718	44.97722
218	11	1786	3.723937	1	0.786402718	44.97722
218	3	1786	8.192661	1	0.825294542	50.01557
218	3	1786	8.192661	1	0.825294542	50.01557

218	3	1786	8.192661	1	0.825294542	50.01557
218	3	1786	8.192661	1	0.825294542	50.01557
218	3	1786	8.192661	1	0.825294542	50.01557
218	3	1786	8.192661	1	0.825294542	50.01557
218	7	1786	4.68152	1	0.833152024	51.83224
218	7	1786	4.68152	1	0.833152024	51.83224
218	7	1786	4.68152	1	0.833152024	51.83224
218	17	1786	2.891527	1	0.847327679	54.45
218	17	1786	2.891527	1	0.847327679	54.45
218	12	1786	3.413609	1	0.851655498	55.88414
218	12	1786	3.413609	1	0.851655498	55.88414
139	39	1786	6.91865	2.65E-10	2.65E-10	4.62E-10
139	49	1786	4.982235	4.29E-06	2.14E-06	7.48E-06
139	64	1786	4.216052	1.43E-05	4.76E-06	2.49E-05
139	46	1786	4.748514	8.21E-05	2.05E-05	1.43E-04
139	16	1786	8.030576	4.89E-04	9.77E-05	8.52E-04
139	33	1786	4.672335	0.015484578	0.002597573	0.027209
139	22	1786	5.256377	0.110685178	0.016618081	0.20434
139	41	1786	3.76066	0.137721303	0.018351613	0.25805
139	29	1786	4.430662	0.163320732	0.019617737	0.310454
139	24	1786	4.818345	0.210297292	0.02333334	0.410852
139	24	1786	4.818345	0.210297292	0.02333334	0.410852
139	24	1786	4.818345	0.210297292	0.02333334	0.410852
139	24	1786	4.818345	0.210297292	0.02333334	0.410852
139	38	1786	3.719424	0.298145699	0.031672086	0.615439
139	33	1786	3.893612	0.408501256	0.042814409	0.911459
139	27	1786	4.282974	0.442135096	0.043902387	1.012561
139	28	1786	4.13001	0.534456858	0.053146361	1.324341
139	39	1786	3.294595	0.855158538	0.120856991	3.313013
139	33	1786	3.504251	0.913907611	0.142102707	4.186128
139	41	1786	3.133883	0.938943881	0.151655388	4.758531
139	6	1786	8.565947	0.998892533	0.31483343	11.19031
139	7	1786	7.34224	0.999987238	0.447393459	17.84028
139	7	1786	7.34224	0.999987238	0.447393459	17.84028
139	7	1786	7.34224	0.999987238	0.447393459	17.84028
139	69	1786	2.234595	0.999997803	0.478695371	20.32256
139	3	1786	12.84892	0.999999752	0.515300332	23.295
139	14	1786	4.5889	0.999999945	0.532307569	25.28838
139	4	1786	9.636691	1	0.717895517	39.80237
139	4	1786	9.636691	1	0.717895517	39.80237
139	4	1786	9.636691	1	0.717895517	39.80237
139	4	1786	9.636691	1	0.717895517	39.80237
