# Supplementary material for: Analysis of Immune Gene Expression Subtypes Reveals Osteosarcoma Immune Heterogeneity
Source: J Oncol. 2021 Mar 1;2021:6649412. doi: 10.1155/2021/6649412 (PMC7939746; doi:10.1155/2021/6649412)
Supplement: Supplementary Materials — Supplementary Figure S1. Consensus matrix for immune-related gene modules derived from consensus clustering analysis. Supplementary Figure S2. Expression feature of immune subtypes in training TARGET cohort. Supplementary Figure S3. The validation of immune subtypes in the independent GEO cohort. Supplementary Figure S4. The intracluster heterogeneity revealed by the immune landscape analysis and different subcluster molecular characteristics of patients in immune subtypes 1 and 4 are shown. Supplementary Table S1. The bulk RNA-seq data retrieved from the OS-project. Supplementary Table S2. The collected 1989 immune-related genes. Supplementary Table S3. The immune subtype annotation at the patient level in the TARGET cohort. Supplementary Table S4. The immune-related gene modules feature. Supplementary Table S5. Functional enrichment analysis of gene modules. Supplementary Table S6. The immune-related molecular features of the TARGET patients. Supplementary Table S7. Correlation of average gene module scores between molecular characteristics and immune subtypes. [file 6649412.f1.zip › 6649412.f1/Supplementary Table S6.pdf]

features	TARGET.40	TARGET.40	TARGET.40	TARGET.40	TARGET.40	TARGET.40	TARGET.40	TARGET.40
Adipocytes	0	0.0011	0.0025	0.0013	0	0	0	0.0128
Astrocytes	0	0	0	0	0	0	0	0
B-cells	0	0	0	0	0.0044	0	0	0
Basophils	0.3163	0.0143	0.0376	0.0182	0.0067	0.0012	0	0
CD4+ T-ce	0	0	0	0	0	0	0	0
CD4+ Tcm	0.006	0	0.0067	0	0	0	0.0057	0.0057
CD4+ Tem	0.0025	0.0532	0.0054	0.0194	0.0108	0.0011	0.0101	0.0048
CD4+ mer	0	0	0	0.001	0.0043	0	0	0
CD4+ naiv	0	0	0	0	0	0	0	0
CD8+ T-ce	0	0	0	7.00E-04	0.0042	0	0	0.0052
CD8+ Tcm	0	0	0	0.0234	0	0	0	0
CD8+ Tem	0	0	0	0	0	0	0	0
CD8+ naiv	0	0.0096	4.00E-04	0.0076	0.011	0	0	0
CLP	0.0035	0.0161	0	0.0122	0.0303	0.0155	0.0128	0.0056
CMP	0	7.00E-04	0	0	0	0.0019	8.00E-04	0.0016
Chondrocy	0	0.0251	0.023	0.041	0.1086	0.1082	0.039	0.1896
Class-switc	0.0179	0	0.0114	0	0.0143	0	0.0182	0
DC	0	0.0082	1.00E-04	3.00E-04	0	0.0131	0	0
Endothelial	0	0.059	0.0645	0.0151	0.0116	0.0207	0.1748	0.0613
Eosinophils	0	0	8.00E-04	0	0	0	0.0073	0
Epithelial c	0.0085	0.0259	0.0166	0.002	0.0249	0.0167	0.0012	0.0243
Erythrocyte	0	0	0	0	0	0	0	0
Fibroblasts	0.0276	0.0912	0.1644	0.1501	7.00E-04	0.181	0.1288	0.2184
GMP	0	0	0	0	0	0	0	0
HSC	0.187	0.1706	0.155	0.0718	0.0038	0.1158	0.1399	0.1451
Hepatocyte	0	2.00E-04	1.00E-04	0	0	2.00E-04	0	0
Keratinocy	0	0	0	0	0.004	0	0	0
MEP	0	0.0341	0.0471	0.0333	0.0695	0.0411	0.0088	0.0329
MPP	0	0	0	0	0	0	0	0
MSC	0.2853	0.0424	0.0502	0.0107	0.0684	0.2089	0.2509	0.0029
Macropha	0	0.0823	0.0095	0.043	0.0151	0	0.0199	0.026
Macropha	0	0.0554	0.0111	0.0433	0.0185	0.0091	0.011	0.0327
Macropha	0.0057	0.0228	8.00E-04	0.023	0	0	0.0353	0.0154
Mast cells	0	0.013	0.0071	0	0	0.0023	0	0
Megakary	0	0.0027	0.0013	4.00E-04	4.00E-04	0.002	0.0045	9.00E-04
Melanocyt	0.0078	0.0024	0.0012	0.0073	0.007	0.0067	0.0046	0.0103
Memory B	0	0	0	0	0.0125	0	0	0
Mesangial	0	0.0082	0.0145	0	0	0	0	0.0408
Monocytes	0	0.058	0	0.0175	0.0081	0.0259	0.0182	0.0132
Myocytes	0.0199	0.0246	1.00E-04	0.1788	0.1294	0.0067	0	0.0193
NK cells	0	0	0	0	0	0	0	0
NKT	0.0331	0.0328	0.039	0.0152	0.0423	0.0943	0.0405	0.1175
Neurons	0.013	0	0.0068	0.0019	3.00E-04	9.00E-04	0.0019	0.002
Neutrophil	0	0	0	0	2.00E-04	0.0017	0	0
Osteoblast	0	0.0413	0	0.0532	0.0486	0.0218	0	0
Pericytes	0	0.008	0.0713	0	0	0.0387	0.022	0
Plasma cell	0.0123	0.0277	0.0199	0.0127	0.0103	0.0206	0.0039	0.0036
Platelets	0	0	0	0	0	0	0	0
Preadipocy	0	0	0	0	0.0833	0	0	0
Sebocytes	0	0	0	0	0.008	4.00E-04	0	0
Skeletal mu	0	0.0036	0	0.1318	0.0567	0	0	0

Smooth mu	0	0.0988	0.1183	0.2181	0.1683	0.1684	0.1559	0.1642
Tgd cells	0	0	0	0	0	0	0	0
Th1 cells	0	0.0014	0.0244	0.0128	0.0263	0.004	0.0082	0
Th2 cells	0.0836	0.0194	0.0096	0	0.0709	0.0194	0.0281	0.0272
Tregs	0	0	0	0.0093	0	0.0033	0	0.001
aDC	0.0404	0.2182	0.1479	0.2178	0.1085	0.2046	0.197	0.2007
cDC	0	0.0032	0.0095	0.0011	0.0108	0.0458	0.055	0.0114
iDC	0.1732	0.2147	0	0.0018	0	0.1858	0	0.0855
ly Endothel	0	0.0285	0.0483	0.0105	0.0059	0.0082	0.0921	0.0319
mv Endoth	0	0.0295	0.031	0.0027	2.00E-04	0.0065	0.0778	0.0363
naive B-ce	0	0	0	0	0	0	0	0
pDC	0.0506	0.0194	0	0.0151	0.0345	0	0	0.0074
pro B-cells	0	0	0	0	1.00E-04	0	0	0
ImmuneSc	0	0.1077	0.0117	0.041	0.0213	0.0287	0.0303	0.0296
StromaSco	0.0138	0.0756	0.1157	0.0832	0.0061	0.1008	0.1518	0.1463
Microenvir	0.0138	0.1833	0.1274	0.1242	0.0275	0.1295	0.1821	0.1758

TARGET.4C	TARGET.4C	TARGET.4C	TARGET.4C	TARGET.4C	TARGET.4C	TARGET.4C	TARGET.4C	TARGET.4C
0	0	8.00E-04	0	0	0	0	0	0
0	0	0	0	0.0344	0	0	0	0
0.0199	0	0	0	0	0.0223	0	0	0.0212
0.2117	0.0636	0	0.0909	0.0997	0.0171	0.093	0	0.1566
0	0	0	0	0	0	0	0	0
0.0341	0.0078	0	0	0.0023	0	0	0	0.0215
0	0.0558	0	0.0729	0	0.0263	0.0037	0	0
0	0	0	0	0	0.0059	0	0	0
0	0	0	0	0	0	0	0	0
0	0	0	0	0	7.00E-04	0	0	0
0	0	0	0	0	0.0124	0	0	0
0	0	0	0	0	0	0	0	0
0	0	0	0	0	0.0126	0	0	0
0.0172	0	0.0279	0	0.0046	0.0227	0	0.0398	0.0259
0	0	0.0037	0	0.0015	0	0.0013	0.0222	0
0	0.0277	0.1393	0.0118	0.0017	0	0.0781	0.1371	0
0.0059	6.00E-04	0	0	0.005	0.0085	0	0.0025	0.0118
0	0.0026	0	0.0042	0	0.0011	0	0	0
0.0287	0.1446	0.0479	0.252	0	0.1305	0.0594	0	0.0334
0	0.0038	0	0.0069	0	0	0	0	0
0	0.0126	0.0084	0.01	0.0034	0.0043	0.0029	0.0087	0.0172
0	0	0	0	0	0	0	0	0
0	0.1095	0.1967	0.0905	0.0776	0.0477	0.1594	0.2175	0.0971
0	0	0	0	0	0.0082	0	0.0486	0
0.0123	0.1341	0.1933	0.275	0.0305	0.1334	0.1213	0.1396	0.0179
0	0	0	0.0046	0	0	0.0012	0	0
0	0	0	0	0	0	0	0	0
0	0.026	0.0196	0.0515	0.0269	0.0055	0.0382	0.0099	0.0071
0	0	0	0	0	0	0	0	0
0.1296	0.2834	0.1421	0	0.0835	0.0705	0.2412	0.1038	0.3288
0.0118	0.0581	0	0.0772	0.031	0.0583	0.0344	0	0.0446
0.0107	0.0514	0	0.0612	0.0375	0.0473	0.021	0.0045	0.0264
0.0147	0.0496	0	0.0485	0.0028	0.0237	0.0382	0.0071	0.0239
0	0.0059	0	0.0032	6.00E-04	0.0021	8.00E-04	0	0.0165
0	9.00E-04	0.001	0	0	9.00E-04	0	0	0
0.0124	0.0142	0.0137	0.0059	0.0126	0.0058	0.0163	0.0257	0.008
0	0	0	0	0	0.0023	0	0	0
0	0	0	0.0012	0.0056	0	0	0	0
0	0.0408	0	0.0361	0.0056	0.0311	8.00E-04	0	0.0166
0.0323	0.0087	0.1579	0.0373	0.021	0.0155	0.0227	0.0091	0.0249
0	0	0	0	0	0	0	0	0
0.0549	0.0521	0.1653	0.1309	0.0945	0.0374	0.0757	0.1378	0.0916
0.004	2.00E-04	0.0038	9.00E-04	0.0053	2.00E-04	0	0.0074	0.0021
0	0	0	0.0021	0	0	8.00E-04	0	0
0.004	0	0.0221	0	0.0362	0.0572	0.0244	0	0.028
0	0	0	0.0498	0	0	0.0208	0	0.0775
0.025	0	0.0045	0	0	0.0203	0.0024	0.0314	0.0101
0	0	0	0	0	0	0	0	0
0	0	0	0.0478	0	0.0066	0	0	0
0	0	0	0	0	0	0	0.0024	0
0.0069	0.0021	0.1707	0.0141	0	0.0029	0.0051	0	0

0.1586	0	0.0999	0.1024	0.0789	0.1198	0.0345	0.148	0
0	0	0	0	0.0027	0.0103	0	0	0
0	0.0188	0.0127	0.0019	0.0155	0	0.0115	0	0.0315
0	0	0.0249	0.0768	0.0148	0.0451	0.0611	0.0034	0
0	0	0	0	0	0.0044	0	0.0165	0.0065
0.1007	0.2866	0.0592	0.3	0.0864	0.2653	0.1044	0.1366	0.1899
0	0.0079	0	0.026	0	0.0049	0	0.0141	0.0054
0	0.0851	0	0.0583	0	0.0074	0	0	0
0.0116	0.1051	0.0262	0.1678	0	0.0614	0.0268	0	0.0291
0.0038	0.0931	0.02	0.1421	0	0.0614	0.0253	0	0.0215
0.0018	0	0	0	0	0.0032	0	0	0
0.0017	0.0126	0.0146	0.0036	0	0.0142	0.0242	0	5.00E-04
0	0	0	0	0	0	0	0	0
0.0211	0.0741	0	0.0865	0.0248	0.0771	0.0245	0	0.0659
0.0144	0.127	0.1227	1.71E-01	0.0388	0.0891	0.1094	0.1088	0.0652
0.0355	0.2012	0.1227	0.2577	0.0636	0.1662	0.1339	0.1088	0.1312

TARGET.4C	TARGET.4C	TARGET.4C	TARGET.4C	TARGET.4C	TARGET.4C	TARGET.4C	TARGET.4C	TARGET.4C
0	0	0	0	0	0	0	0.0013	0
0	0	0	0	0	0	0	0	0
0.0064	0	0	0	0	0	0	0	0
0	0.317	0	0	0.0274	0.0433	0.0863	0.3387	0.1336
0	0	0	0	0	0	0	0	0
0	0.001	0.0016	0	0.0177	0	0	0	0.0082
0.0122	0.0282	3.00E-04	0.0088	0	0.0296	0.0583	0.0155	0
0	0	0	0.0072	0	0	0	0	0.0084
0.0079	0	0	0	0	0	0	0	0
1.00E-04	0	0	0	0	0	0.0105	0	0
0.0202	0	0	0	0	0	0.082	0	0
0	0	0	0	0	0	0	0	0
0	0	0	0.0042	0.0085	0.0063	0.0176	0	0
0.0065	0	0.0263	0.0307	0.0194	0.0143	0.0141	0	7.00E-04
0.006	0	0.0056	0	0.0105	0	0.002	0	0
0	0	0	0.0051	0.0134	0	0.0607	0	0
0.00E+00	0	0	0.0021	0	0.0124	0.0144	0.0309	0.01
0	0	0	0	0	0	2.00E-04	0	0
0.0212	0.0644	0.1956	0.0171	0.116	0.0799	0.1309	0.0147	0.0554
0	0	0	0	0	0	0	0	0
0.0152	7.00E-04	0.0086	0.0271	0.0056	0.0145	0.0074	0	0.0012
0	0	0	0	0	0	0	0	0
0	0	0	0.1416	0.052	0.0769	0.0828	0.0262	0.1254
0.0094	0	0.0184	0	0.004	0	0	0	0
0.1312	0	0.2119	0.1647	0.2699	0.0356	0.256	0.0083	0.0738
0	0.0029	0	0	0.0014	0	0	0	0
0	0	0	0	0	0	0	0	0
0	0.033	0.0213	0.0274	0.0143	0.0502	0.0288	0.0671	0.0016
0	0	0	0	0	0	0	0	0
0.4682	3.67E-01	0.3369	0.3946	0.1639	0.267	0.4162	0.1382	0.0418
0.0437	0.0377	0.0169	0	0.0372	0.0702	0.0699	0.0437	6.00E-04
0.0249	0.0217	0.0228	0	0.0253	0.0391	0.0632	0.0459	0.0109
0.0034	0.0097	0.0126	0	0.0408	0.0144	0.0235	0.0193	0.004
0.0072	0.0036	0	0	0	0.0013	0	0.0023	0.0032
0.0037	0	0.0018	0.0043	0.001	0	0	0	0
0.0114	0.0119	0.0201	0.008	0.0115	0.0158	0.0052	0.0064	0.005
0	0.0076	0	0	0	0	0	0.0047	0
0	0	0	0	0	0	0	0	0
0.0091	0	0	0	0.0238	0.0314	0.0241	0	0
0.0175	0.0221	0.0371	0.025	0.0189	0.0202	0.307	0.0184	0.012
0	0	0	0	0	0	0	0	0
0.0495	0.0572	0.1191	0.0295	5.00E-04	0.0782	0.0422	0.0742	0.0072
0.0013	0	0.0061	0.0043	0.0014	1.00E-04	0.0062	0.0024	0.0015
0	0	0	0	1.00E-04	0	0	0	0
0	0	0.0219	0.0041	0	0	0	0.0088	0.0731
0	0.0172	0	0	0	0	0	0	0
0.0604	0.0127	0.0084	0.0188	0	0.0048	0	0.0014	0.0086
0	0	0	0	0	0	0	0	0
0	0	0	0	0	0	0	0	0.019
0	0	0	7.00E-04	0	0	0	0	0
0	0	0	0	0.0185	0.0047	0.2073	0.0047	0.0013

0.1436	0	0	0.0906	0.1252	0.0424	0	0.1141	0.1391
0.004	0	0	0.0013	1.00E-04	0	0	0	0
0	0.0323	0	0.0146	0	0.0116	0.0168	0.0785	0
0	0.0055	0.0358	0.0308	0.0128	0.0373	0.0739	0.0406	0.1298
0.0156	0	0.0084	0	0	0	0	0	0
0.1093	0.0454	0.1577	0.0232	0.1881	0.1279	0.3142	0.1646	0.0559
0.0733	0	0.0158	0.0017	0.0035	0.0095	0.0088	0	0
0	0.0505	0	0	0.0071	0.025	0.0985	0.0209	0.0215
0.0088	0.0382	0.0903	0.009	0.0645	0.0483	0.065	0.0076	0.0251
0	0.0487	0.0906	0.004	0.0504	0.0449	0.0903	0.0161	0.0263
0	0.0064	0	0	0	0	0	0	0
0	0.0012	0.0046	0	0	0.0363	0	0	0.0032
0.00E+00	0.0067	0	0	0	0	0	0.013	0
0.0443	0.0275	0.0113	0	0.0407	0.0686	0.0698	0.0307	0.0025
0.0106	0.0322	0.0978	0.0794	0.084	0.0784	0.1068	0.0211	0.0904
0.0549	0.0597	0.1091	0.0794	0.1247	0.147	0.1766	0.0518	0.0929

TARGET.4C	TARGET.4C	TARGET.4C	TARGET.4C	TARGET.4C	TARGET.4C	TARGET.4C	TARGET.4C	TARGET.4C
0	0	0	0	0	0	0	0	0
0	0	0	0	0	0	0	0	0
0	4.00E-04	0	0	0	0	0	0.0081	0
0.1259	0.0716	0.0029	0.0101	0.0296	0.047	0.0665	0.1121	3.00E-04
0	0	0	0	0	0	0	0	0
0.0161	0	0	0	0	0	0	4.00E-04	0
0.0364	0.0444	0.1074	0	0	0.0273	0.0316	0.0209	0.0239
0	0	0	0.0022	0	0	0	0	0.0018
0	0	0	0	0	0	0	0	0
0	0	0	0	0	0	0	0	0
0	0	9.00E-04	0	9.00E-04	0	0	0	0
0	0	0.0091	0	0	0	0	0	0
0.0011	0	0.0034	0.0035	0.0052	0	0	0.0039	0.0112
0.0087	0	0.0129	0.0153	0.0146	0	0.0093	0.0146	0.0119
0.0096	0.0025	2.00E-04	0	0.0081	0.0032	0	0	0
0.0054	0	0	0	0.0398	0.0367	0	0	0
0.0012	0.001	0.0027	0	0	0.0025	0	0.0041	7.00E-04
0.0072	0	0	0	0	0	0	0	0
0.1518	0.0214	0.1378	0.0736	0.0237	0.0746	0.0447	0.0119	0.1495
0	0	0	0.0046	0	0	0	0	0.0039
0.0078	0.0194	0.0045	0	0.0011	0.0039	0.0099	0.0025	0.0166
0	0	0	0	0	0	0	0	0
0.1513	0	0.0857	0.1849	0.2139	0.0932	0.0659	0	0.0588
0	0	0	0	0	0.06	0	0	6.00E-04
0.2381	0	0.0914	0.1161	0.1955	0.1672	0.0334	0	0.199
0.0036	0	0	0	0	4.00E-04	2.00E-04	0	0.0014
0	0	0	0	0	0	0	0	0
0.0636	0.0388	0.0574	0.0134	0.0223	0.0436	0.0281	0.1251	0.0324
0	0	0	0	0	0	0	0	0
0.1597	0.5325	0.2353	0.4129	0.1478	0.2723	0.2572	0.583	0.0956
0.0407	0.046	0.0232	0	0.0198	0.0498	0.0422	0.001	0.0447
0.0368	0.0526	0.0442	0	0.0196	0.0412	0.0287	0.008	0.0389
0.0235	0.0259	0.0162	0.0041	0.0053	0.0298	0.0074	0.0078	0.0099
0.0019	0.0012	0	0	0.0012	0	0.0031	3.00E-04	0.0025
0	0	5.00E-04	0.0022	0	0	0	0	0.0041
0.0035	0.011	0.0106	0.0057	0.0141	0.0087	0.0156	0.007	0.0087
0	0.0163	0	0	0	0	0	0.005	0
0.0491	0	0	0	0	0	0	0	0.005
0.0526	0.0342	0.0177	0	0	0.0313	0	0	0.0258
0.0106	0	0.0126	0.0057	0.0114	0.0097	0.0183	0.0489	0.0232
0	0	0	0	0	0	0	0	0
0.0518	0.1128	0.1053	0.0115	0.0326	0.1735	0.1016	0.0775	0.077
0.0027	4.00E-04	0	0.0011	0.0018	0.0021	0	0.0032	0.0026
8.00E-04	0	0	6.00E-04	0	0.0026	0	0	6.00E-04
0.0274	0	0.0829	0	0.0669	0.0439	0.0442	0.0025	0
0.0333	0.0195	0.0336	0.0242	0	0.012	0.0023	0	0.0089
0	0.006	0.0106	0.0039	0.0041	0.0196	0.006	0.0065	0.0171
0	0	0	0	0	0	0	0	0
0	0	0	0	0	0	0	0	0.0033
0	0	0	0	0	0	0	0	0
0.0014	0	0.0016	0	0	0	9.00E-04	0	0.0134

0.0306	0	0	0.2667	0.2052	0.0103	0.0616	0	0.0333
0	0	0	0	0	0	0	0	0
0.0574	0.0646	0.0616	0	0	0.0397	0.0333	0.0877	0.0134
0.0055	0.0264	0.0511	0.138	0.0847	0	0.0159	0.1147	0.0072
0	0	0	0	0	0	0	0	0.0082
0.1767	0.1787	0.195	0.0994	0.1551	0.1949	0.0442	0.1025	0.1441
0.0315	0	0.0075	0.0081	0.0082	0	0	0	1.00E-04
0.3365	0	0	0	0	0	0	0	0
0.0819	0.013	0.0977	0.0448	0.0091	0.0318	0.0219	0.0107	0.0824
0.1265	0.0283	0.0948	0.0301	0.013	0.0459	0.0317	0.0143	0.0681
0	0	0	0	0	0	0	0.0015	0
0	0.0019	0	0	0	0	0.0094	0.0531	0
0	0	0	0	0	0	0	0.0089	0
0.0688	0.0545	0.0273	0.0035	0.014	0.0558	0.0302	0.0063	0.0517
0.1516	0.0107	0.1118	0.1292	0.1188	0.0839	0.0553	0.006	0.1042
0.2204	0.0652	0.139	0.1327	0.1328	0.1397	0.0855	0.0122	0.1558

TARGET.4(	TARGET.4(	TARGET.4(	TARGET.4(	TARGET.4(	TARGET.4(	TARGET.4(	TARGET.4(	TARGET.4(
0	0	0	0.0012	0	0	0	0	8.00E-04
0	0	0	0	0	0	0	0	0.0059
0	0	0	0	0	0	0	0	0.0061
0.0013	0.0315	0.0095	0	0	0	0	0.007	0.153
0	0	0	0	0	0	0	0	0
0	0.0067	0	0	0	0.029	0.0033	0	0.0283
0	0.0253	0.0218	0.039	0	0.0208	1.00E-04	0	0.0712
0	0	0	0	0	0	0	0	0
0	0	0	0	0	0	0.0026	0	0
0	0	0	0	0	0.0041	0	0.0095	0
0	0	0	0.0013	0	9.00E-04	0	0	0
0	0	0	0	0	0	0	0	0
0.0263	0.0065	0	0.0208	0.0025	0.0105	0	0.0096	0
0.0361	0	0.0025	0.0138	0.0078	0.0034	0.0011	0.0113	0
0	0	0	0	4.00E-04	0.001	0.0033	0	0
0.0216	0.0263	0.0147	0.0792	0.0646	0.0387	0.1104	0.0229	0
0.0033	0	0	0	0.0026	0	0.0076	0.0062	0.0042
0.0075	0.0066	0	0	0	0	0	0	0
0	0.0621	0.0756	0.0485	0.0319	0.0611	0.0302	0.015	0.1191
0	0	0	0	0	0.0016	0	0	0
0.0051	0.013	0.0246	0.0217	0.0033	0.0085	0.0091	0.0021	0.0091
0	0	0	0	0	0	0	0	0
0.1312	0.1719	0.0955	0.1645	0.2565	0.133	0.0885	0.2382	0
0	0	0	0	0.0027	0	0	0	0
0.1207	0.149	1.44E-01	0.2167	0.2124	0.1092	0.1088	0.1433	0.0616
0	0.0016	0	0	0	0	0	0	0
0.0021	0	0	0	0	0	0	0	0
0.0534	0.0656	0.0346	0.0542	0.0106	0.0319	0.0219	0.0299	0.0226
0	0	0	0	0	0	0	0	0
0.1367	0.0435	0.3123	0.3012	0.23	0.3015	0.3514	0.1909	0.2011
0	0.0507	0.0439	0.0617	0.0133	0	0.0173	0.0253	0.0832
0	0.0265	0.0282	0.0412	0.0155	0.0023	0.0362	0.0253	0.0616
0.0017	0.0412	0.0279	0.0081	0.0061	0	0.0163	0.0298	0.0505
0	0	0	0	0	0.003	2.00E-04	0	0.0028
0	0.0011	0	2.00E-04	9.00E-04	0.001	0.0017	3.00E-04	0
0.0048	0.0085	0.0156	0.0066	0.0132	0.0049	0.0174	0.0064	0.002
0	0	0	0	0	0	0	0	0
0	0.0143	0	0	0	0	0	0	0.0078
0	0.0039	0.0065	0.0079	0.0109	0	0.0062	0.0075	0.0371
0.0094	0.224	0.0137	0.0424	0.0126	0.0039	0.0056	0.01	0.0013
0	0	0	0	0	0	0	0	0
0.0253	0.0996	0.1048	0.0154	0.044	0.0516	0.1764	0.0436	0.1166
0.0027	0	0.0014	0.0039	0.0032	0.0018	0.0011	1.00E-04	0.0021
7.00E-04	0	0.0014	0	0	0	0.0015	2.00E-04	0
0.0769	0.0763	0.0571	0	0	0.039	0.0288	0.0102	0.02
0	0.0288	0	0	0	0.0286	0.0085	0.1065	0.0065
0.0032	0.017	0	5.00E-04	0.0158	0.0209	0.0079	1.00E-04	5.00E-04
0	0	0	0	0	0	0	0	0
0	0	0	0	0	0	0	0	0
0	0	0	0	0	0	0.0014	0	0
0	0.2388	4.00E-04	6.00E-04	0	0	0	0	0

0.1759	0	0.0522	0.1036	0.2423	0.0372	0	0.2535	0
0	0	0	0	0	0	0	0	0
0.0355	0.0589	0.0128	0	0	0.0263	0.0057	0	0.0271
0.1139	0	0.0241	0.0204	0.023	0	0	0.05	0
0	0	0	0	0	0	0	0	0
0.0619	0.1479	0.1291	0.1286	0.1209	0.0631	0.1401	0.193	0.2322
0.0343	0.0028	0	0.0288	0.0019	0.0062	0	0.0176	0.0013
0.1398	0.2154	0	0.0789	0	0	0	0	0.1434
0	0.0376	0.0444	0.0284	0.011	0.0385	0.0047	0.0066	0.0781
0	0.0439	0.0383	0.0252	0.0096	0.0455	0.0124	0.0087	0.0825
0	0	0	0	0	0	0	0	0
0.0189	0.0791	0	0.056	0	0	0	0.0079	0.0029
0	0	0	0	0	0	0	0	0
0.0055	0.0408	0.0345	0.0464	0.0161	0.0058	0.0168	0.0283	0.0861
0.0656	0.117	0.0856	0.1071	0.1442	0.097	0.0594	0.1266	0.06
0.0711	0.1578	0.1201	0.1535	0.1603	0.1028	0.0762	0.1549	0.1461

TARGET.40	TARGET.40	TARGET.40	TARGET.40	TARGET.40	TARGET.40	TARGET.40	TARGET.40	TARGET.40
0	0	0	0	0	0	0	0.0059	0
0	0	0	0	0	0	0	0	0
0	0	0.004	0	0.0017	0.0097	0.0056	0	0
0.0087	0	0.1008	0.01	0.0215	0.275	0.1373	0.0936	0.0439
0	0	0	0	0	0	0	0	0
0	0	0	0	0.0023	0	0	0	0
0.0437	0.0074	0.0371	0.0567	0.0219	0	0.068	0.0446	0.0619
0	0	0	0.0052	0.0087	0.0085	3.00E-04	0	0
0	0	0	0	0	0	0	0	0
0	0	0	0	0.0085	0	0	0	0
0.0071	0.0039	0	0	0	0	0.0052	3.00E-04	0
0	0	0	0	0	0	0	0	0
0.0126	0.0185	0	0	0.0017	0	0.0021	0.0011	0
0.0306	0.0199	0.0097	0.0176	0.0517	0.046	0.0299	0.0048	0.0095
0.0037	0.0048	0	0	0	0	0	0.0066	0
0.0268	0.0436	0	0	0	0	0	0	0
0.0088	0.0018	0.0029	0.0158	0.0072	0.0298	0.0049	0	0.0055
0.0029	0	0	1.00E-04	0	0	0	0.0076	0
0.0962	0.062	0.0079	0.1158	0.0128	0.0645	0.0806	0.0972	0.1613
0	0	0	0.0139	0	0	0	0	0
0.0148	0.0064	0.0103	0.0132	0.007	0.0053	6.60E-03	0	0.0099
0	0	0	0	0	0	0	0	0
0.0592	0.0912	0	0.0756	0	0	0	0.067	0
0.0013	0	0	0	0	0	0	0	0
0.165	2.24E-01	0	0.1109	0.002	0.1833	0.0149	0.1258	0.0973
0	0	0.0015	0	0	5.00E-04	0	0	0
0	0	0	0	0	0	0	0	0
0.0614	0.0663	0.0729	0.0326	0.0668	0.0507	0.0512	0.0286	0.0524
0	0	0	0	0	0	0	0	0
0.1899	0.2635	0.1689	0.1397	0.0075	0.0729	0.4673	0	0.2973
0.1159	0.0796	0.0502	0.1172	0.0136	0	0.0177	0.0794	0.0452
0.0701	0.056	0.0533	0.0606	0.009	0.0043	0.0106	0.0667	0.0398
0.0509	0.0265	0.0126	0.0422	0	0.0108	0.0111	0.0661	0.0153
0.003	0	0.0048	0.0043	0.0049	0	0	0.009	0
0.0057	0.0019	0	0.0014	0.0015	0.0076	0	0.0023	0.0015
0.0131	0.0112	0.0059	0.0077	0.0054	0.0094	0.0204	0.0243	0.0194
0	0	0	0	0.005	0	0.005	0	0
0	0	0	0	0	0	0	3.00E-04	0
0.0444	0.0389	0.0303	0.054	0.004	0	0	0.0363	0.0292
0.0128	0.0142	0.0066	0.018	0.0319	0.0156	0.0406	0.023	0.0245
0	0	0	0	0	0	0	0	0
0.0343	0.018	0.0417	0.04	0.0265	0.0163	0.052	0.0938	0.0752
0	7.00E-04	0.0012	0.0019	0.0024	0.0216	7.00E-04	0	0.0019
0	0	0	0	0	0	0	0	0
0.0239	0.0328	0.0555	0.0042	0.0499	0	0	0.0459	0
0	0	7.00E-04	0	0	0	0	0	0
9.00E-04	0.004	0	0	0.0099	0.0037	8.00E-04	0	0.0125
0	0	0	0	0	0	0	0	0
0	0	0	0	0.0406	0	0	0	0
0	0	0	0	0	0	0	0	0
0	0	0.001	0	0.0082	0.0034	0.0026	0	0.0035

0.1192	0.1642	0.0346	0.0679	0.2798	0	0	0.1928	0.0854
0	0	0	0	0	0	0	0	0
0.0212	0.005	0.0727	0	0.0059	0.0178	0.0288	0	0
0.0673	0.1127	0.0112	0	0.1286	0	0.0588	0.0358	0.0396
0	0	0.0018	0	0	0	0	0.0087	0
0.2802	0.2294	0.1486	0.2401	0.0582	0.0716	0.141	0.2131	0.231
0.0244	0.0437	0	0.0132	0.0015	0.0016	0	0.0399	0.0221
0.077	0.007	0	0.1118	0	0	0	0.1745	0
0.0593	0.0315	0	0.0552	0.0064	0.0344	0.0508	0.0398	0.0966
0.0514	0.0299	0.0075	0.0566	0.0021	0.0224	0.0486	0.0455	0.0798
0	0	0	0	0.001	0	0.0024	0	0
0.0043	0	0.0038	0.0141	0.0147	0.0486	0	0.0031	0.0035
0	0	0	0	0.0088	0	0	0	0
0.1108	0.079	0.0595	0.1263	0.0218	0.0065	0.0155	0.0882	0.0496
0.0777	0.0766	0.004	0.0957	0.0064	0.0322	0.0403	0.085	0.0806
0.1885	0.1556	0.0635	0.222	0.0282	0.0387	0.0558	0.1733	0.1302

TARGET.4C	TARGET.4C	TARGET.4C	TARGET.4C	TARGET.4C	TARGET.4C	TARGET.4C	TARGET.4C	TARGET.4C
0	0	0	0.0021	0	0	0	0	0
0	0	0	0	0	0	0	0	0
0	0	0.007	0	0	0	0	0.0167	0
0	0	0.0408	0.1611	0.0086	0	0.0045	0.0232	0.01
0	0	0	0	0	0	0	0	0
0	0	0	0	0	0	0	0	0
0	0.0664	0.0901	0	0.0091	0	0	0.0473	0
0	0.0146	0.0126	0	0.0145	0.0106	0	0.009	0
0	0	0	0	0	0	0	0	0
0	0	0	0	0	0	0	0	0
0	0.0248	0	0	0.0079	0.0019	0	0.0116	0.0017
0	0	0	0	0	0	0	0	0
0.0013	8.00E-04	0.0035	0	0.0013	0.001	0.0254	0.0035	0
0.0177	0.0157	0.0295	0.0191	0.0189	0.028	0.0309	0.0146	0.0065
0.0018	0.0158	0	0	0	0	0	0	1.00E-04
0	0	0	0.0665	0	0	0.0083	0.0647	0.1441
0.0076	0	0.0043	0.0174	0	0	0	0.0287	0
0	0.0034	0	0	0	0	0	0	0
0.0499	0.1329	0.0229	0.0311	0.0539	0.1061	0.0184	0.0469	0.0515
0	0	0	0	2.00E-04	0	0	0	0
0.0077	0.0079	0.0095	0.0057	0.0035	0.0108	0	3.00E-04	0.0049
0	0	0	0	0	0	6.00E-04	0	0
0.0592	0.0616	0.044	0.2365	0.1247	0.0684	0	0.2087	0.1522
0.0046	0.0359	0	0	0.0028	0	0	0.0102	0.0325
0.1718	0.1309	0.0051	0.0594	0.0429	0.1685	0.2578	0.0357	0.2502
0	0	0	5.00E-04	0	0	0	0	0
0	0	0	0	0	0	0	0	0
0.0168	0.0397	0.0238	0	0.0128	0.0499	0.0431	0.0209	0.0132
0	0	0	0	0	0	0	0	0
0.0939	0.1611	0.0697	0.1851	0.0402	0.0384	0	0.1222	0.3123
0.0119	0.0449	0.0496	0	0.0467	0.0683	0	0.1357	0.0221
0.0152	0.0329	0.0078	0.0029	0.0218	0.0406	0	0.0765	0.021
0.0076	0.0172	0.0183	0.0054	0.0055	0.0355	0	0.0485	0.0032
0	0	0.0103	2.00E-04	0.0041	0	0.0015	0.0112	0
0.0021	0.0028	5.00E-04	0	0	2.00E-04	0.0015	0.0012	5.00E-04
0.013	0.0126	0.0111	0.0148	0.015	0.017	0	0.0081	0.0089
0	0	0	0	0	0	0	0.0053	0
0	0	0	0	0	0	0	0	0
0	0.0267	0	0	0.006	0.0338	0	0.0788	0.0057
0.0404	0.0612	0.0215	0.0074	0.0245	0.028	0.4191	0.0055	0.0023
0	0	0	0	0	0	0	0	0
0.0307	0.0384	0.0515	0.0333	0.0671	0.0325	0.0351	0.0238	0.0204
0.0013	1.00E-04	0.0027	0.0048	8.00E-04	0.0012	0.0359	8.00E-04	0.0042
0	0	0	0	3.00E-04	0	0.0057	0	0.0013
0.0471	0.0523	0.0532	0	0.0662	0.0307	0	0	0
0	0	0	0	0	0	0	0.0424	0.0138
0.017	0.0074	0.0138	0	0.0176	0	0	0.0011	0
0	0	0	0	0	0	0	0	0
0	0	0.0186	0	0.0078	0	0	0	0
0	0	0	0	0	0	0	0	0
0.0093	0.0305	3.00E-04	0	6.00E-04	0.006	0.3439	0	0

0.2161	0.1866	0.3019	0.0862	0.2503	0.241	0.2174	0.2923	0.1498
0	0	0.0166	0	0	0.0059	0	0	0
0	0	0	0	0	0	8.00E-04	0	0
0.1059	0.0815	0.0258	0.0158	0.0936	0.0585	0.0231	0.0708	0.0011
0.0165	0	0	0.0099	0	0	0	0	0
0.1226	0.1774	0	0.0793	0.031	0.1783	0	0.2802	0.1509
0.0096	0.0252	0	0.0024	0.0365	0	0	0.0356	0.018
0	0.1098	0	0	0.0035	0	0.0191	0	0
0.027	0.0715	0.0107	0.0171	0.0189	0.0414	0.0053	0.0164	0.0251
0.0167	0.0675	0.009	0.0045	0.0166	0.0421	0.0033	0.021	0.0216
0	0	2.00E-04	0	0	0	0	0	0
0	0	0.0131	0	5.00E-04	0.0122	0	0.0062	0
0	0	0.0047	0	0	0	0	0	0
0.0079	0.05	0.0446	1.00E-04	0.0382	0.0681	0.0048	0.1616	0.0194
0.0546	0.0972	0.0334	0.1348	0.0893	0.0872	0.0092	0.1278	0.1018
0.0625	0.1472	0.0781	0.135	0.1275	0.1553	0.014	0.2894	0.1212

TARGET.4(	TARGET.4(	TARGET.4(	TARGET.4(	TARGET.4(	TARGET.4(	TARGET.4(	TARGET.4(	TARGET.4(
0	0	0	0	0.0101	0	0	0	0
0	0	0	0	0	0	0	0	0
8.00E-04	0	0.0029	0.0284	0.0193	0	0	0	0
0.0278	0.0215	0.0478	0.1882	0.0055	0.0916	0	0.1943	0.0361
0	0	0	0	0	0	0	0	0
0	0	0	0	0	0	0	0.0115	0.0098
0.0817	0.038	0.0237	0.1398	0.0734	0	0	0.0243	0.0597
0.0019	0.0098	0.0246	0	2.00E-04	0.0019	0	0	0.0067
0	0	0	0	0	0	0	0	0
0	0	0	0.1231	0.1329	0	0	0	0
0.0039	0	0	0.1786	0.1639	0	0	0.0032	0
0	0	0	0.0838	0.0372	0	0	0	0
0	0.0144	0.0068	0.0423	0.0447	0.0156	0.0201	0.0276	0
0.0071	0.0122	0.0389	0.0101	0.0082	0.0349	0.0059	0.0038	0.018
0	0	0	0	0	0.0012	0.0054	0.0076	0
0	0	0.0459	0	0.0328	0.0047	0	0	0.0034
0.0124	0.0016	0.0073	0.0169	0.0317	0.0045	0	0.0306	0
0.0012	0.0012	0	9.00E-04	0.0209	0	0	0.0288	0
0.051	0.1296	0	0.0048	0.1567	0.011	0.1104	0.0535	0
0	0	0	0	0	0	0	0	0
0.0076	0.0112	7.00E-04	0.0089	0.0072	1.00E-04	0.0058	0.0025	0.0052
0	0	0	0	0	6.00E-04	0	0	0
0	0	0.1297	0.0103	0.1253	0.1259	0.2319	0	0.1054
0	0.0014	0	0	0.0136	0	0.0639	0	0
0.0619	0.0945	0.0136	0	0.2669	0.0434	0.3682	0.1354	0.0461
0	0	0	0	0	0	0	0	0
0	0	0	0	0	0	0	0	0
0.0425	0	0.0187	0.0423	0.0052	0.0328	0.0261	0.0618	0.0315
0	0	0	0	0	0	0	0	0
0.1608	0.3186	0	0.2217	0.0513	0.0175	0.1566	0.558	0.2207
0.0759	0.063	0.0036	0.0548	0.1412	0.0102	0.013	0.0255	0
0.0654	0.0608	0	0.0589	0.0946	0.0112	0.016	0.0191	0.0013
0.0398	0.0156	0	0.0269	0.057	0.0148	0.0252	0.0163	0
9.00E-04	0	0.0042	9.00E-04	0	6.00E-04	0	0	1.00E-04
1.00E-04	0.0047	0	0	0.0095	0	0.0067	0	0
0.0167	0.0102	0.0036	0.0089	0.0071	0.0013	0.0193	0.0024	0.0174
0	0	5.00E-04	0.0079	0.0032	0	0	0	0
0	0	0.0108	0	0	0	0	0	0
0.0249	0.0362	0	0.0186	0.0647	0.0028	0	0.0051	0
0.014	0.0222	0.0666	0.0262	0.2398	0.3467	0.0141	0.038	0.0093
0	0	0	0	0	0	0	0	0
0.0491	0.0152	0.0818	0.0894	0.083	0.0515	0.0311	0.0548	0.0147
6.00E-04	0.0033	0.0019	0.0012	0.0013	0.0013	0.0019	0	0.0049
0	0	0	0	0	3.00E-04	0	0	0
0.0194	0	0.0241	0	0	0.0316	0.0069	0	0.0431
0	0.0055	0	0	0	0	0	0	0
0.0132	0.0075	0.0067	0.0014	0.0096	0.0092	0.0054	0	0.0078
0	0	0	0	0	0.0175	0	0	0
0.0031	0	0	0	0	0.0213	0	0	0
0	0	0	0	0	0	0	0	0
0.0042	0.0027	0.0199	0	0.3877	0.2681	0.0059	0.0094	0

0.1285	0.0897	0.1957	0.0476	0.0712	0.1845	0.0686	0.013	0.1914
0	0	0.0076	0	3.00E-04	0	0	0	0
0.0208	0	0	0.039	0.0027	0	0.0073	0.0677	0.0256
0.0614	0.0625	0.0493	0.1347	0.0459	0.0633	0.014	0.0064	0.0327
0	0.0124	0.0037	0	0	0	0	0	0
0.2764	0.2512	0.0191	0.3199	0.3796	0.1053	0.1066	0.3296	0.0208
0.0103	0.0212	0.0098	0	0.0897	0	0.0076	0.0164	0.003
0	0	0.0013	0.0084	0.2371	0.0434	0	0.5044	0
0.0241	0.0529	0	0.0027	0.0842	0.0057	0.0554	0.0373	0
0.0276	0.053	0	0.0064	0.076	0.0076	0.0605	0.0491	0
0	0	0.0022	0	0	0	0	0	0
0.0072	0.0476	0.0017	0.1288	0.1042	0	0	0	0
0	0	0	0	0	0.0092	0	0	0.0027
0.0691	0.0669	0.0071	0.1511	0.2527	0.0093	0.0087	0.0396	1.00E-04
0.0255	0.0648	0.0648	0.0076	0.1461	0.0685	0.1712	0.0268	0.0527
0.0946	0.1317	0.072	0.1587	0.3987	0.0777	0.1798	0.0663	0.0528

TARGET.40	TARGET.40	TARGET.40	TARGET.40	TARGET.40	TARGET.40	TARGET.40	TARGET.40	TARGET.40
0	0	0	0.0028	0	0	0	0	0
0	0	0	0	0	0	0	0	0
0	0	0	0	0	0	0	0	0
0.183	0.0189	0.0105	0.0221	0.2477	0.0606	0.0669	0.0557	0.0434
0	0	0	0	0	0	0	0	0
0	0.0025	0.0185	0	0.0143	0	0	0	0.0027
0.03	0.0298	0.044	3.00E-04	0.0385	0.0143	0.0248	0.1241	0
0.0083	0	0	0	0	0	0	0.0021	0
0	0	0	0	0	0	0	0	0
0	0	0	0	0	0	0	0	0
0	0	0	0	0	0	0	0.0123	0
0	0	0	0	0	0	0	0	0
0	0	0.0127	0	0	0	0	0.0024	0
0.0209	0.0129	0.0046	0.009	0	0.0334	0.0054	0.0148	0.021
0	0	0.0026	0	0	0	0	0.0033	0.006
0	0	0	0.035	0	0.0266	0	0	0.1736
7.00E-04	0	0.0062	0	0.0096	0	0.0076	0.0049	0
0	9.00E-04	0	0	0	0	0	0.0011	0
0.1324	0.0474	0.0843	0.018	0.05	0.0177	0.2075	0.0802	0.0034
0	0	0	0	0	0	0.0028	0	0.0019
0.0029	0.0102	0.0069	0.0152	0.0058	0.0029	0.0056	0.0107	0.0039
0	0	0	0	0	0	0	0	0
0.0472	0.0587	0.0246	0.181	0	0.3061	0.0553	0	0.116
0	0	0.0055	0	0	0	0	0	0
0.0702	0.1294	0.076	0.2457	0.0524	0.1149	0.162	0.031	0.1177
0.0038	0	0	0	0.0044	0.0012	0.001	0	7.00E-04
0	0	0	0	0	0	0	0	0
0.0192	0.0185	0.0236	0.0321	0	0.0288	0.0181	0.0516	0.0072
0	0	0	0	0	0	0	0.0031	0
0.1152	0.0416	0.1053	0.1271	0.2507	0.2642	0.1474	0.346	0.0422
0.1058	0.0236	0.0254	0.06	0	0.0352	0.0823	0.0505	0
0.0675	0.0312	0.0244	0.0277	0.0301	0.0348	0.0492	0.0591	5.00E-04
0.0439	0	0.0105	0.0236	0.0021	0.018	0.0456	0.0214	0
0.0027	0.0045	0	7.00E-04	0.0032	0.0042	0.0023	0	1.00E-04
0	0	0.0028	0	0	0	5.00E-04	0	0
0.0072	0.0049	0.0077	0.0097	0.0172	0.0166	0.0081	0.0174	0.0112
0	0	0	0	0.0055	0	0	3.00E-04	0
0	0	0	0	0	0	0	0	0
0.05	0.0111	0.0123	0	0	0.0166	0.0441	0.0112	0
0.0253	0.0169	0.1718	0.0188	0.0753	0.0272	0.0467	0.0304	0.0108
0	0	0	0	0	0	0	0	0
0.0267	0.1405	0.0822	0.0538	0.0986	0.1238	0.101	0.2254	0.0455
0.0021	6.00E-04	0.0014	0	0.0094	0.0024	0.001	6.00E-04	0.0021
0	4.00E-04	3.00E-04	0	0	0	0	0	0
0.027	0.1118	0.0014	0	0.0065	0	0.0302	0	0.0286
0.004	0	0	0	0.0198	0.023	0	0	0.0123
5.00E-04	0.0219	0.0084	0	0.003	0.0046	0	0.0293	0.0107
0	0	0	0	0	0	0	0	0
0	0	0	0	0	0	0.0076	0	0
0	0	0	0	0	0	0	0	0
0.0107	0.003	0.1785	3.00E-04	0.0395	0	0.0171	0.0116	0

0.1389	0.1647	0.0647	0.1468	0	0.0664	0	0	0.1014
0	0	0	0.0058	0	0	0	0	0
0.007	0	0.0235	0	0.0132	0	0.011	0.113	0.0181
0.0748	0.0672	0.0419	0.0236	0	0.0776	0	0	0.0468
0	0.01	0	0	0	0.0052	0	0.0152	0
0.2868	0.1802	0.1546	0.0739	0.0954	0.1738	0.2235	0.2887	0.0423
0.0078	0.0357	0.0385	4.00E-04	0	0	0.0942	0.0053	0
0.0397	0	0	0.0147	0	0	0.023	0	0
0.069	0.0199	0.0403	0.0096	0.0151	0	0.1242	0.0573	0
0.0632	0.0244	0.029	0.0048	0.03	0.0036	0.1182	0.0515	0
0	0	0	0	0.0016	0	0	0	0
0.0818	0	0	0.0516	0	0.0023	2.00E-04	0.07	0
0	0	0	0	0.0031	0	0	0	0
0.1057	0.027	0.0253	0.0405	0.0021	0.0373	0.0877	0.0419	0.0013
0.0898	0.053	0.0544	0.1009	0.025	0.1619	0.1314	0.0401	0.0597
0.1955	0.08	0.0798	0.1414	0.0271	0.1992	0.2191	0.082	0.061

TARGET.4C	TARGET.4C	TARGET.4C	TARGET.4C	TARGET.4C	TARGET.4C	TARGET.40.PAVECB
0	0.0016	0.0019	0	0	0	0
0	0	0	0	0	0	0
0	0	0	0	0	0.0275	0
0.2527	0	0.1076	0.007	0	0.118	0
0	0	0	8.00E-04	0	0	0
0	0	0.0465	0	0	0.0113	0
0.0041	0.0116	0.0785	0	0.0299	0.0035	0
0	0	0.0196	0	0	0.0245	4.00E-04
0	0	0	0	0	0	0
0	0	0	0.0052	0	0	0
0	0	0	0.0106	0	0	0
0	0	0	0	0	0	0
0	0	0	0.0056	0	0	0
0.0269	0.0343	0.0481	0.0391	0.0201	0.0383	0.0065
0	0	0	0	0.0011	0.0038	0.0141
0	0.0121	0.0692	0.0345	0	0	0.1176
0	0	0	0.0125	0	0.0466	0.001
7.00E-04	0	0	0	0.0023	9.00E-04	0
0.0337	0.0403	0.076	0	0.1061	0.0445	0.0372
0	0	0	0	0.0011	0	0
0	0.005	0	4.00E-04	0.0096	0.0021	0.0016
0	0	0	0	0	0	0
0	0.0996	0.2161	0.1376	0.0844	0	0.2402
0	0	0	0	0	0	0.0053
0.0977	0.2101	0.1681	0.088	0.2885	0.0487	0.2502
0.002	0	0.0041	0	0	0	0
0	0	0	0	0	0	0
8.00E-04	0.0104	0.0219	0.0499	0.0463	0.0105	0
0	0	0	0	0	0	0
0.1901	0.2725	0.0013	0.2183	0.2718	0.3806	0.2158
0.0071	0.0074	0	0	0.0795	0.0356	0.0177
0.0072	0.0056	0	0	0.0555	0.0198	0.0094
0.0028	0	0	0	0.0261	0.0137	0.006
0	0	0	0.0013	0	0	0
0	0	0.0016	0	0.0047	2.00E-04	3.00E-04
0.007	0.0168	0.005	0.0118	0.0135	0.0034	0.005
0	0	0	0	0	0	0
0	0	0	0	0	0	0.0035
0	0	0	0	0.0215	0.032	0
0.0243	0.0284	0.0242	0.0126	0.0039	0.0457	0.0142
0	0	0	0	0	0	0
0.0404	0.1836	0.0998	0	0.0315	0.0109	0.0068
0.0046	0.001	0.009	0.0027	0	0.0027	0.0059
0	0	0	0	0	0	0
0	0	0	0	0.0148	0	0
0.0076	0	0.0707	0	0.0559	0	0
0.0094	0.0097	0.0036	0.0201	0.0092	0.0324	0.0181
0	0	0	0	0	0	0
0	0	0.0132	0	0	0	0
0	0	0	0	2.00E-04	0	0
0.0074	0.0043	0.0106	0	0	0.0094	0

0.1273	0.0523	0.0624	0.1617	0.0616	0.0507	0.165
0	0	0	0	0	0	0
0	0.0134	0.0027	0.0207	0.0315	0	0
0.0649	0	0.0584	0.1227	0.0148	0.034	0.0088
0	0	0	0	0	0.009	0
0.1967	0	0.0323	0	0.2576	0.1937	0.1697
0.0081	0	0	2.00E-04	0.0173	0.0113	0.0093
0	0.0295	0.0027	0.067	0.0825	0.1228	0.0041
0.0178	0.0141	0.044	0	0.0508	0.0335	0.0217
0.0076	0.0076	0.0303	0	0.0465	0.0119	0.0155
0	0	0	0	0	0.0039	0
0.0065	0	0	0.0261	0	0	0
0	0	0	0	0	0	0
0.0052	0.0049	0	0.0049	0.0696	0.064	0.0118
0.0168	0.0707	0.147	0.0688	0.0952	0.0222	0.1387
0.022	0.0757	0.147	0.0737	0.1648	0.0862	0.1505
