# Supplementary material for: Analysis of Immune Gene Expression Subtypes Reveals Osteosarcoma Immune Heterogeneity
Source: J Oncol. 2021 Mar 1;2021:6649412. doi: 10.1155/2021/6649412 (PMC7939746; doi:10.1155/2021/6649412)
Supplement: Supplementary Materials — Supplementary Figure S1. Consensus matrix for immune-related gene modules derived from consensus clustering analysis. Supplementary Figure S2. Expression feature of immune subtypes in training TARGET cohort. Supplementary Figure S3. The validation of immune subtypes in the independent GEO cohort. Supplementary Figure S4. The intracluster heterogeneity revealed by the immune landscape analysis and different subcluster molecular characteristics of patients in immune subtypes 1 and 4 are shown. Supplementary Table S1. The bulk RNA-seq data retrieved from the OS-project. Supplementary Table S2. The collected 1989 immune-related genes. Supplementary Table S3. The immune subtype annotation at the patient level in the TARGET cohort. Supplementary Table S4. The immune-related gene modules feature. Supplementary Table S5. Functional enrichment analysis of gene modules. Supplementary Table S6. The immune-related molecular features of the TARGET patients. Supplementary Table S7. Correlation of average gene module scores between molecular characteristics and immune subtypes. [file 6649412.f1.zip › 6649412.f1/Supplementary Table S7.pdf]

immCells	pvalue	FDR
Adipocytes	5.43E-01	6.56E-01
Astrocytes	5.99E-01	6.56E-01
B-cells	0.400369778	0.550508445
Basophils	1.65E-03	7.78E-03
CD4+ T-cells	2.24E-01	4.48E-01
CD4+ Tcm	4.16E-01	5.61E-01
CD4+ Tem	8.98E-04	4.56E-03
CD4+ memory T-cells	3.82E-01	5.48E-01
CD4+ naive T-cells	6.06E-01	6.56E-01
CD8+ T-cells	2.99E-01	5.19E-01
CD8+ Tcm	0.328669182	0.531987215
CD8+ Tem	0.332429779	0.531987215
CD8+ naive T-cells	0.195746348	0.41675029
CLP	6.03E-03	2.34E-02
CMP	9.60E-02	2.62E-01
Chondrocytes	5.03E-05	3.69E-04
Class-switched memory B-cells	1.27E-01	2.99E-01
DC	3.11E-02	1.03E-01
Endothelial cells	1.12E-04	6.71E-04
Eosinophils	1.41E-01	3.21E-01
Epithelial cells	1.91E-02	7.01E-02
Erythrocytes	3.47E-01	5.32E-01
Fibroblasts	1.65E-09	2.18E-08
GMP	6.99E-01	7.33E-01
HSC	2.22E-01	4.48E-01
Hepatocytes	4.29E-01	5.66E-01
Keratinocytes	5.84E-01	6.56E-01
MEP	0.711812361	0.734056497
MPP	0.354985193	0.53247779
MSC	0.344855126	0.531987215
Macrophages	6.58E-12	2.00E-10
Macrophages M1	1.29E-10	2.13E-09
Macrophages M2	2.13E-08	2.34E-07
Mast cells	0.022019766	0.076489714
Megakaryocytes	0.506643174	0.630914142
Melanocytes	0.277065205	0.494224419
Memory B-cells	6.69E-01	7.13E-01
Mesangial cells	2.60E-01	4.94E-01
Monocytes	2.48E-12	1.64E-10
Myocytes	6.02E-01	6.56E-01
NK cells	NA	NA
NKT	4.86E-01	6.17E-01
Neurons	4.14E-02	0.130230956
Neutrophils	3.80E-01	5.48E-01
Osteoblast	1.22E-01	2.98E-01
Pericytes	4.59E-01	5.94E-01
Plasma cells	3.18E-01	0.531987215
Platelets	2.68E-01	4.94E-01
Preadipocytes	8.45E-01	8.58E-01
Sebocytes	5.84E-01	6.56E-01
Skeletal muscle	8.92E-01	8.92E-01

Smooth muscle	2.75E-01	4.94E-01
Tgd cells	1.19E-01	2.98E-01
Th1 cells	0.559635012	0.655818607
Th2 cells	1.83E-01	4.03E-01
Tregs	5.90E-01	6.56E-01
aDC	1.19E-07	1.12E-06
cDC	0.392966854	0.550508445
iDC	0.001958455	0.008617202
ly Endothelial cells	0.000422785	0.002325319
mv Endothelial cells	9.43E-05	0.000622165
naive B-cells	0.099141401	0.261733298
pDC	0.077455629	0.232366888
pro B-cells	0.08913913	0.255790547
ImmuneScore	9.09E-12	2.00E-10
StromaScore	0.003802298	0.01568448
MicroenvironmentScore	7.57E-07	6.25E-06
