# Supplementary figures and images for: Analysis of Immune Gene Expression Subtypes Reveals Osteosarcoma Immune Heterogeneity
Source: J Oncol. 2021 Mar 1;2021:6649412. doi: 10.1155/2021/6649412 (PMC7939746; doi:10.1155/2021/6649412)

consensus matrix k=7

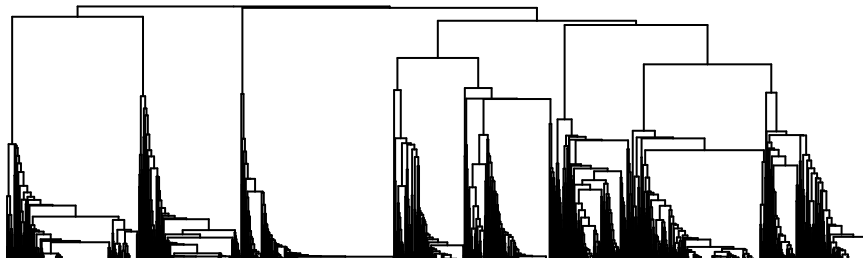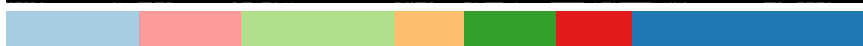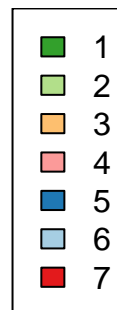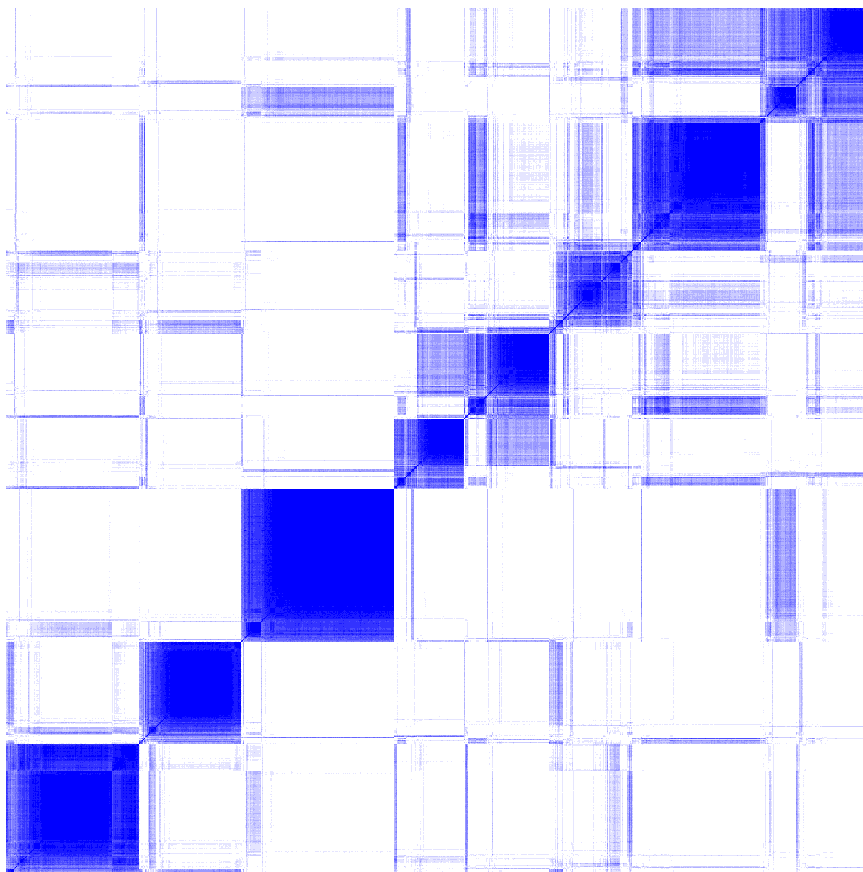

Supplement: Supplementary Materials — Supplementary Figure S1. Consensus matrix for immune-related gene modules derived from consensus clustering analysis. Supplementary Figure S2. Expression feature of immune subtypes in training TARGET cohort. Supplementary Figure S3. The validation of immune subtypes in the independent GEO cohort. Supplementary Figure S4. The intracluster heterogeneity revealed by the immune landscape analysis and different subcluster molecular characteristics of patients in immune subtypes 1 and 4 are shown. Supplementary Table S1. The bulk RNA-seq data retrieved from the OS-project. Supplementary Table S2. The collected 1989 immune-related genes. Supplementary Table S3. The immune subtype annotation at the patient level in the TARGET cohort. Supplementary Table S4. The immune-related gene modules feature. Supplementary Table S5. Functional enrichment analysis of gene modules. Supplementary Table S6. The immune-related molecular features of the TARGET patients. Supplementary Table S7. Correlation of average gene module scores between molecular characteristics and immune subtypes. [file 6649412.f1.zip › 6649412.f1/Supplementary Figure S1.pdf]

## TCGA\_OS\_IS

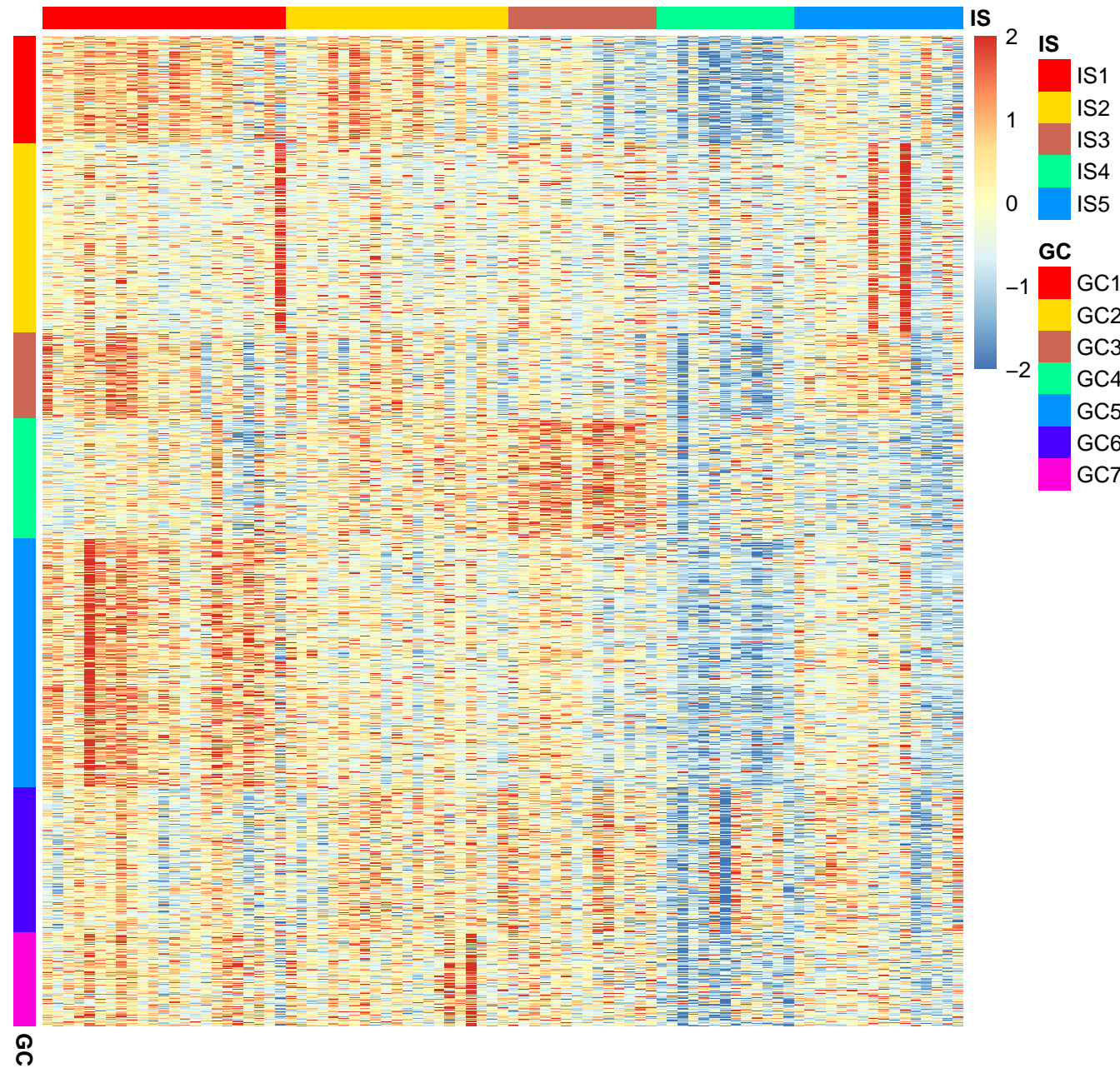

Supplement: Supplementary Materials — Supplementary Figure S1. Consensus matrix for immune-related gene modules derived from consensus clustering analysis. Supplementary Figure S2. Expression feature of immune subtypes in training TARGET cohort. Supplementary Figure S3. The validation of immune subtypes in the independent GEO cohort. Supplementary Figure S4. The intracluster heterogeneity revealed by the immune landscape analysis and different subcluster molecular characteristics of patients in immune subtypes 1 and 4 are shown. Supplementary Table S1. The bulk RNA-seq data retrieved from the OS-project. Supplementary Table S2. The collected 1989 immune-related genes. Supplementary Table S3. The immune subtype annotation at the patient level in the TARGET cohort. Supplementary Table S4. The immune-related gene modules feature. Supplementary Table S5. Functional enrichment analysis of gene modules. Supplementary Table S6. The immune-related molecular features of the TARGET patients. Supplementary Table S7. Correlation of average gene module scores between molecular characteristics and immune subtypes. [file 6649412.f1.zip › 6649412.f1/Supplementary Figure S2.pdf]

GEO\_OS\_IS

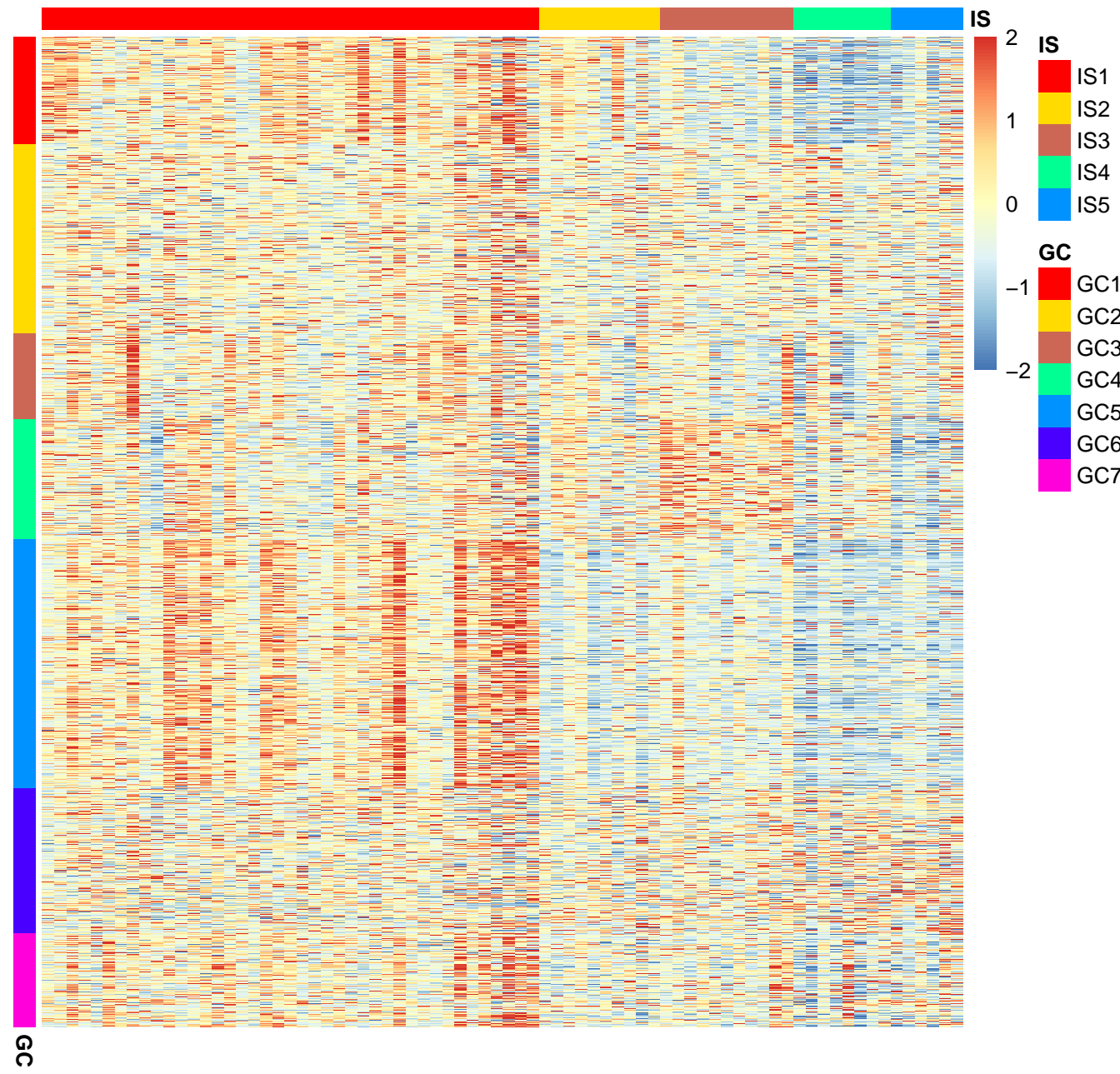

Supplement: Supplementary Materials — Supplementary Figure S1. Consensus matrix for immune-related gene modules derived from consensus clustering analysis. Supplementary Figure S2. Expression feature of immune subtypes in training TARGET cohort. Supplementary Figure S3. The validation of immune subtypes in the independent GEO cohort. Supplementary Figure S4. The intracluster heterogeneity revealed by the immune landscape analysis and different subcluster molecular characteristics of patients in immune subtypes 1 and 4 are shown. Supplementary Table S1. The bulk RNA-seq data retrieved from the OS-project. Supplementary Table S2. The collected 1989 immune-related genes. Supplementary Table S3. The immune subtype annotation at the patient level in the TARGET cohort. Supplementary Table S4. The immune-related gene modules feature. Supplementary Table S5. Functional enrichment analysis of gene modules. Supplementary Table S6. The immune-related molecular features of the TARGET patients. Supplementary Table S7. Correlation of average gene module scores between molecular characteristics and immune subtypes. [file 6649412.f1.zip › 6649412.f1/Supplementary Figure S3.pdf]

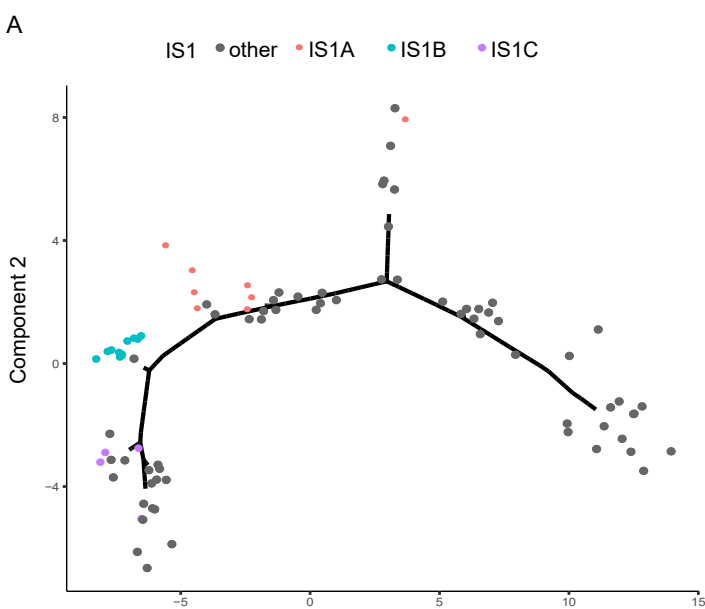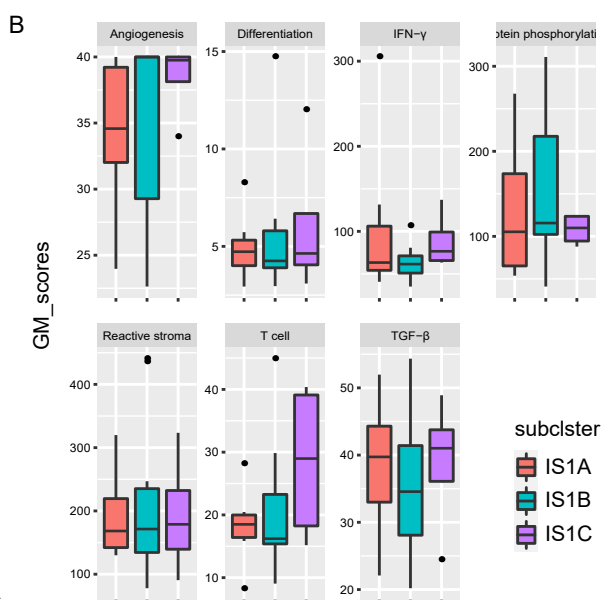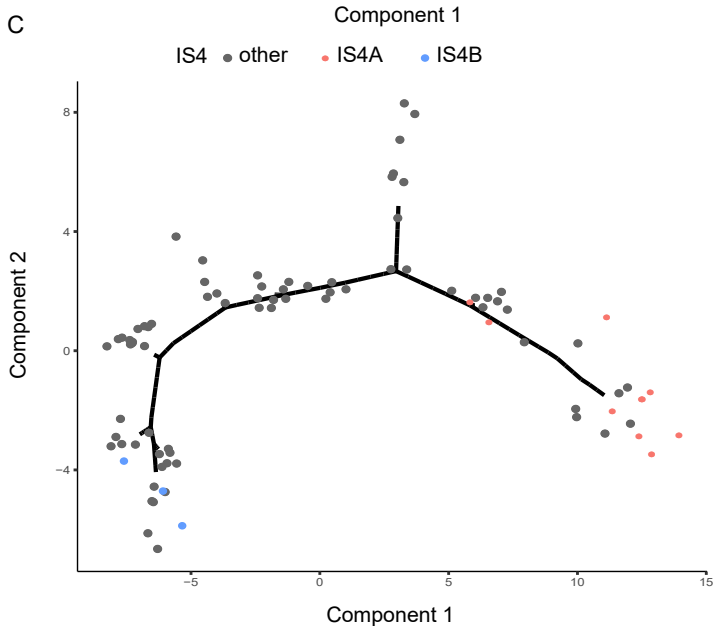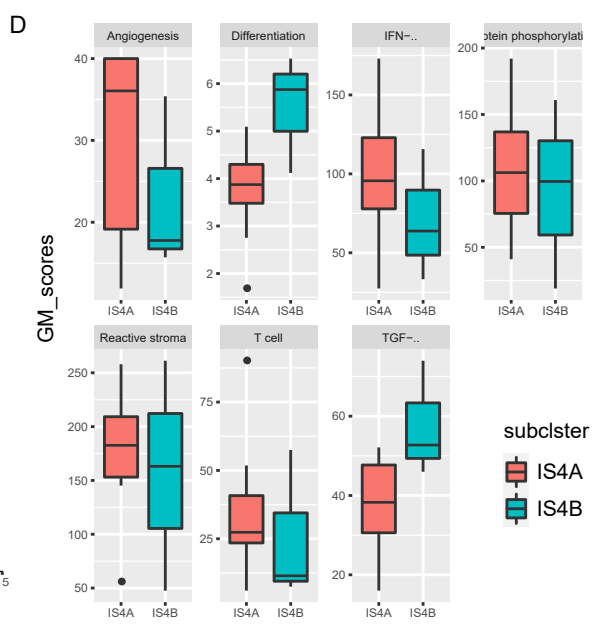

Supplement: Supplementary Materials — Supplementary Figure S1. Consensus matrix for immune-related gene modules derived from consensus clustering analysis. Supplementary Figure S2. Expression feature of immune subtypes in training TARGET cohort. Supplementary Figure S3. The validation of immune subtypes in the independent GEO cohort. Supplementary Figure S4. The intracluster heterogeneity revealed by the immune landscape analysis and different subcluster molecular characteristics of patients in immune subtypes 1 and 4 are shown. Supplementary Table S1. The bulk RNA-seq data retrieved from the OS-project. Supplementary Table S2. The collected 1989 immune-related genes. Supplementary Table S3. The immune subtype annotation at the patient level in the TARGET cohort. Supplementary Table S4. The immune-related gene modules feature. Supplementary Table S5. Functional enrichment analysis of gene modules. Supplementary Table S6. The immune-related molecular features of the TARGET patients. Supplementary Table S7. Correlation of average gene module scores between molecular characteristics and immune subtypes. [file 6649412.f1.zip › 6649412.f1/Supplementary Figure S4.pdf]
